# *PFMG2025*–integrating genomic medicine into the national healthcare system in France

**DOI:** 10.1016/j.lanepe.2024.101183

**Published:** 2025-01-06

**Authors:** PFMG2025 contributors, Caroline Abadie, Aldja Abderrahmane, Ouarda Abdous, Carine Abel, Oanez Ackermann, Cécile Acquaviva, Flavie Ader, Salma Adham, Dalila Adjaoud, Alexandra Afenjar, Nathalie Aladjidi, Anne-Sophie Alary, Frédérique Albarel, Sabrina Albert, Lise Allard, Ingrid Allix, Violaine Alunni, Inês F. Amado, Cyril Amouroux, Nicolas André, Chloé Angelini, Mathieu Anheim, Ignacio Antolin Sanfelliz, Thomas Aparicio, Chloé Arfeuille, Jean-Benoît Arlet, Lionel Arnaud, Pauline Arnaud, Guilhem Arnold, Tania Attie-Bitach, Marion Aubert-Mucca, Isabelle Audo, Marie-Pierre Audrezet, Maxime Auroux, Céline Auzanneau, Xavier Ayrignac, Ibrahima Ba, Anne Bachelot, Delphine Bacq, Séverine Bacrot, Brigitte Bader-Meunier, Sarah Baer, Stéphanie Baert-Desurmont, Laurence Bal-Theoleyre, Ralyath Balogoun, Philippe Baltzinger, Guillaume Banneau, Claire Bar, Audrey Barbet, Giulia Barcia, Laure Barjhoux, Anne Barlier, Vincent Barlogis, Marc Barritault, Magalie Barth, Aurore Barthod-Malat, Peggy Baudouin-Cornu, Geneviève Baujat, Amandine Baurand, Jacques-Olivier Bay, Michèle Beau-Faller, Jean-Christophe Beaudoin, Rémi Bellance, Christine Bellanné-Chantelot, Carine Bellera, Alexandre Belot, Raihane Ben Abdeljelil, Rihab Ben Sghaier, Joy Benadiba, Stéphanie Benard, Claire Beneteau, Karelle Benistan, Fouzia Benkerdou, Mehdi Benkirane, Jean-François Benoist, Patrick R. Benusiglio, Claire-Marine Bérat, Camille Bergès, Anne Bergougnoux, Maureen Bernadach, Emilien Bernard, Valérie Bernard, Virginie Bernard, Dounia Beroug, Aurélie Berrard, Jérôme Bertherat, Pascaline Berthet, Clotilde Berthier, Aurélia Bertholet-Thomas, Jean-Philippe Bertocchio, François Bertucci, Céline Besse, Elsa Besse-Pinot, Didier Bessis, Pauline Beuvain, Stéphane Bezieau, Marie Bidart, Ivan Bièche, Margaux Biehler, Thierry Bienvenu, Frédéric Bilan, Clarisse Billon, Christine Binquet, Elise Bismuth, Varoona Bizaoui, Pierre Blanc, Hélène Blanché, Jean-Yves Blay, Adrien Bloch, Gilles Bloch, Agnès Bloch-Zupan, Béatrice Bocquet, Morgane Boedec, Catherine Boileau, Maureen Boissinot, Anne Boland, Pierre-Adrien Bolze, Valérie Bonadona, Julia Bonastre, Nathalie Bonello-Palot, Adeline-Alice Bonnard, Raphaël Borie, Damien Botsen, Mohamed Bouattour, Marion Bouctot, Natacha Bouhours-Nouet, Jérôme Bouligand, Ahmed Bouras, Thomas Bourgeron, Jean-Louis Bourges, Emmanuelle Bourrat, Guilaine Boursier, Guilhem Bousquet, Philippe-Jean Bousquet, Simon Boussion, Lucile Boutaud, Julian Boutin, Patrice Bouvagnet, Claire Bouvattier, Sandrine Boyault, Aude Brac de la Perriere, Mehdi Brahmi, Valentine Brard, Mathilde Brasseur, Nadège Brazzalotto, Dominique Brémond-Gignac, Audrey Briand-Suleau, Claire Briet, Pierre-Paul Bringuier, Céline Bris, Elise Brischoux-Boucher, Karine Brochard, Martin Broly, Laura Brosseau, Ange-Line Bruel, Perrine Brunelle, Virginie Bubien, Bruno Buecher, Alexandre Buffet, Adrien Buisson, Lydie Burglen, Cyril Burin Des Roziers, Nelly Burnichon, Tiffany Busa, Mathilde Cabart, Sara Cabet, Charlotte Caille-Benigni, Claire Caillot, Christophe Calvin, Anne Cambon-Thomsen, Claude Cances, Alexandre Cantan, Liana Carausu, Aurélia Carbasse, Cédric Carbonneil, Bertrand Cariou, Olivier Caron, Sylvain Carras, Stéphanie Cartalat, Kévin Cassinari, Martin Castelle, Laurent Castéra, Frédéric Castinetti, Julie Catteau, Roseline Caumes, Aurélien Caux, Mathias Cavaillé, Hélène Cavé, Aurélie Caye-Eude, Cécile Cazeneuve, Tristan Celse, Noémie Celton, Camille Cenni, Jasmin Cévost, Rania Chaabna, Brigitte Chabrol, Ilyas Challet, Clélia Chalumeau, Pascal Chambon, Albain Chansavang, Jean-Baptiste Chanson, Sébastien Chapelant, Fabienne Charbit-Henrion, Perrine Charles, Maëlle Charpie, Sybil Charrière, Philippe Charron, Nicolas Chassaing, Nicolas Chatron, Boris Chaumette, Catherine Chaussain, Annabelle Chaussenot, David Cheillan, Olivier Chenavier, Bertrand Chesneau, Louise-Marie Chevalier, Christine Chomienne, Cécile Chougnet, Sophie Christin-Maitre, Marine Chuet, Emmanuelle Clappier, Johanna Clet, Mélanie Cloteau, Thomas Cluzeau, Guillaume Cogan, Benjamin Cogné, Alicia Cohen, Camille Cohen, Odile Cohen-Haguenauer, Martine Cohen-Solal, Chrystelle Colas, Estelle Colin, Corinne Collet, Delphine Collin-Chavagnac, Éloïse Colliou, Marie-Agnès Collonge-Rame, Maxime Colmard, Stéphanie Coopman, Lucie Coppin, Elodie Coquan, Valérie Cormier-Daire, Nadège Corradini, Carole Corsini, Mireille Cossée, Thibault Coste, Sophie Cotteret, Rachel Cottet, Christine Coubes, Florence Coulet, Nathalie Couque, Philippe Couratier, Marie Courbebaisse, Olivier Courbette, Cécile Courdier, Juliette Coursimault, Thomas Courtin, Lucien Courtois, Fabienne Coury, Laure Coutos-Thévenot, Charles Coutton, Isabelle Creveaux, Etienne Crickx, Louise Crivelli, Marc Cuggia, Laurence Cuisset, Hubert Curcio, Aurore Curie, Veronica Cusin, Noémie Da Costa, Lionel Da Cruz, Éric Dahlen, Antoine Dardenne, Benjamin Dauriat, Nell Dausse, Alix De Becdelièvre, Florence De Fraipont, Élisa De La Cruz, Thibault De la Motte Rouge, Pascale De Lonlay, Sandrine De Montgolfier, Antoine De Pauw, Aurélien De Reyniès, Jean-Madeleine De Sainte Agathe, Marie De Tayrac, Anne-Sophie Defachelles, Michaël Degaud, Caroline Deiller, Éric Delabesse, Leslie Delachaux, Andrée Delahaye-Duriez, Brigitte Delemer, Jean-François Deleuze, Hélène Delhomelle, Christelle Delmas, Capucine Delnatte, Catherine Delorme, Richard Delorme, Bénédicte Demeer, Caroline Demilly, Philippe Denizeau, Isabelle Denjoy, Anne-Sophie Denommé-Pichon, Christel Depienne, Nicolas Derive, Flora Dervillé, Vincent Des Portes, Isabelle Desguerre, Béatrice Desnous, Camille Desseignes, Françoise Devillard, Manjula Deville, Nelly Dewulf-Pasz, Claire-Marie Dhaenens, Klaus Dietrich, Anne Dieux, Mody Diop, Emmanuel Disse, Samir Djaber, Christine Do Cao, Hélène Dollfus, Louis Domenach, Jean Donadieu, Bruno Donadille, Aurore Dougé, Hélène Dreyfus, Séverine Drunat, Danièle Dubois-Laforgue, Christèle Dubourg, Charlotte Dubucs, Jean-Christophe Dubus, Matthieu Duchmann, François Ducray, Marion Ducrotverdun, Florence Duffaud, Yannis Duffourd, William Dufour, Gwénaëlle Duhil de Bénazé, Yves Dulac, Olivier Dunand, Denis Dunoyer de Segonzac, Célia Dupain, Nicolas Duployez, Anaïs Dupré, Aurélien Dupré, Sophie Dupuis-Girod, Romain Duquet, Alice Durand, Benjamin Durand, Isabelle Durand-Zaleski, Xavier Durando, Alexandra Durr, Lauriane Eberst, Patrick Edery, Matthieu Egloff, Salima El Chehadeh, Laïla El Khattabi, Camille Engel, Mathilde Entresangle, Hélène Espérou, Florence Esselin, Pascaline Etancelin, Clémence Evrevin, Claire Ewenczyk, Alain Eychene, Thomas Eychenne, Andra Ezaru, Vincent Fabry, Laurence Faivre, Marie Faoucher, Clémentine Faure, Julien Fauré, Anne-Laure Fauret-Amsellem, Eva Feigerlova, François Feillet, Laurène Fenwarth, Claude Férec, Patricia Fergelot, Anthony Ferrari, Carole Ferraro-Peyret, Jean-Paul Feugeas, Claire Fieschi, Alice Fievet, Marc Fila, Rémi Fillatre, Mathilde Filser, Bertrand Fin, Mathieu Fiore, Nelly Firmin, Pascale Flandrin-Gresta, Aude Flechon, Benjamin Fournier, Cécile Fragny, Marie-Céline Francois-Heude, Bruno Francou, Thierry Frébourg, Véronique Fressart, Mathilde Frétigny, Benoit Funalot, Mathieu Fusaro, Pauline Gaignard, Estelle Gandjbakhch, Benjamin Ganne, Aurore Garde, Vincent Gatinois, Céline Gaucher, Léa Gaudillat, Philippe Gaulard, Lucas Gauthier, Mathilde Gay-Bellile, Damien Geneste, David Geneviève, Emmanuelle Genin, Sandrine Genoux, Birgit Geoerger, Véronique Geoffroy, Mathieu Georget, Bénédicte Gérard, Witold Gertych, Souad Gherbi Halem, Karima Ghorab, Romane Gille, Charlène Gillet, Marion Gillibert-Yvert, Olivier Gilly, Anne-Paule Gimenez-Roqueplo, Sophie Giraud, Barbara Girerd, François Girodon, Olga Glazunova, Delphine Gobert, Cyril Goizet, Zeynep Gokce-Samar, Lisa Golmard, Carlos Gomez-Roca, Emmanuel Gonzales, Magali Gorce, Marie-Clémence Gorenstein, Kévin Gorrichon, Frédéric Gottrand, Laetitia Gouas, Stéphanie Gourdon, Pierre Gourdy, Aurélie Gouronc, Claire Goursaud, Gaëlle Gousse, Evan Gouy, Odile Goze-Martineau, Diane Gozlan, David Grabli, Margaux Gras, Maude Grelet, Laetitia Gressin, Nathalie Grivel, Sarah Grotto, Virginie Grouthier, Solange Grunenwald, Olivier Grunewald, Paul Gueguen, Cécile Guérin, Anne-Marie Guerrot, Stéphanie Guey, Nathalie Guffon, Agnès Guichet, Romain Guièze, Marine Guillaud-Bataille, Francis Guillemin, Erell Guillerm, Yann Guillermin, Virginie Guillet-Pichon, Isabelle Guillou, Rosine Guimbaud, Anne Guimier, Claire Guissart, Eric Guittet, Nathalie Guy, Alice Hadchouel, Hamza Hadj Abdallah, Smail Hadj-Rabia, Samy Hadjadj, Mehdi Hage-Sleiman, Corinne Haioun, Sara Halawi, Abderaouf Hamza, Perrine Hanau, Nadine Hanna, Radu Harbuz, Gaëlle Hardy, Carine Hauspie, Sandrine Hayette, Jean-Michel Heard, Maël Heiblig, Solveig Heide, Laurence Heidet, Marcia Henry, Véronique Hentgen, Bénédicte Héron, Delphine Héron, Dominique Hervé, Anthony Herzig, Pierre Hirsch, Antoine Hommais, Jérôme Honnorat, Edgar Horta, Claude Houdayer, Pascal Houillier, Sarah Huet, Jean-Pierre Hugot, Yoann Huguenin, Marc Humbert, Marie-Laure Humbert-Asensio, Laure Huot, Norbert Ifrah, Frédéric Illouz, Apolline Imbard, Marion Imbert-Bouteille, Nicolas Isambert, Bertrand Isidor, Antoine Italiano, Raphaël Itzykson, Sylvie Jaillard, Yvan Jamilloux, Alexandre Janin, Louis Januel, Cécile Javelot-Jacquelin, Médéric Jeanne, Guillaume Jedraszak, Isabelle Jéru, Xavier Jeunemaitre, Éric Jeziorski, Florence Jobic, Philippe Joly, Laurence Jonard, Guillaume Jondeau, Natalie Jones, Jean-Marie Jouannic, Anne Jouinot, Pierre-Simon Jouk, Yohann Jourdy, Kévin Jousselin, Anne Jouvenceau, Charlotte Jubert, Sophie Julia, Anne-Laure Jurquet, Aurélien Juven, Maud Kamal, Pascal Kantapareddy, Elsa Kaphan, Lucie Karayan-Tapon, Edwige Kasper, Lara Kerbellec, Boris Keren, Emmanuel Khalifa, Philippe Khau Van Kien, Sihem Kheddouci, Caroline Kientz, Rathana Kim, Antjie Knapke, Michel Koenig, Isabelle Kone, Marina Konyukh, Raphaël Kormann, Manoëlle Kossorotoff, Paul Kuentz, Florence Kyndt, Anaïs L'Haridon, Philippe Labrune, Marilyn Lackmy, Didier Lacombe, Ludovic Lacroix, Fanny Laffargue, Ghizlene Lahlou, Yec'han Laizet, Laetitia Lambert, Jérôme Lamoril, Audrey Lamouroux, Émilie Landais, Samuel Landman, Elise Landry, Hélène Lapillonne, Anne-Sophie Lapointe, Lise Larcher, Pierre Lardeux, Laetitia Largeaud, Etienne Larger, Louis Larrouquere, Hélène Lasolle, Eulalie Lasseaux, Xenia Latypova, Tiphany Laurens, Camille Laurent, Pierre Laurent-Puig, Géraldine Lautrette, Thomas Lauvray, Benoît Lavallart, Cécile Lavenu-Bombled, Noémie Laverdure, Yannick Le Bris, Catherine Le Chalony, Lauriane Le Collen, Nathalie Le Du, Gaëlle Le Folgoc, Gerald Le Gac, Jessica Le Gall, Edouard Le Guillou, Xavier Le Guillou, Gwenaël Le Guyader, Maryannick Le Ray, Olivia Le Saux, Christophe Le Tourneau, Benjamin Lebecque, Loïc Lebellec, Élise Lebigot, Pierre Leblond, Nicolas Leboulanger, Laure Lebras, Anne-Sophie Lebre, Louis Lebreton, François Lecoquierre, Mathilde Lefebvre, Marine Legendre, Camille Leglise, Clémentine Legrand, Daphné Lehalle, Catherine Lejeune, Christine Lemaitre, Raphaël Leman, Mathis Lepage, Alban Lermine, Karen Leroy, Gaëtan Lesca, Marion Lesieur-Sebellin, Mélanie Letexier, Franck Lethimonnier, Lucie Levaillant, Jonathan Levy, Yves Levy, Pascale Lévy, Ludovic Lhermitte, Agnès Linglart, Clément Lionnet, Doriane Livon, Laurence Lode, Magalie Lodin, Jonathan Lopez, Maureen Lopez, Alain Lortholary, Malek Louha, Camille Louvrier, Thomas E. Ludwig, Auriane Luvet, Stanislas Lyonnet, Caroline Makowski, Valérie Malan, Martial Mallaret, Delphine Mallet, Stéphanie Mallet, Marion Malphettes, Nathalie Manaud, Pierre Mancini, Sylvain Manfredi, Sylvie Manouvrier, Luke Mansard, Sandrine Mansard, Lamisse Mansour-Hendili, Ludovic Mansuy, Julien Maquet, Ambroise Marçais, Alice Marceau-Renaut, Perrine Marec-Berard, Cécilia Marelli, Gaëlle Marenne, Isabelle Marey, Jennifer Margier, Henri Margot, Guillaume Marie, Victor Marin, Laetitia Marisa, Sandrine Marlin, Émeline Marquant, Valentine Marquet, Luisa Marsili, Amaury Martin, Laetitia Martinerie, Anna Maruani, Pauline Marzin, Christophe Massard, Emmanuelle Masson, Flavie Mathieu, Marion Mathieu, Simone Mathoulin-Pelissier, Flore Matthieu, Mathilde Mauras, Aurélien Maureille, Benoit Mazel, Mary Mazeres, Anne Mc Leer, Isabelle Melki, Rita Menassa, Aurélie Méneret, Julie Menjard, Anne Mercier, Elodie Merieau, Marie-Sophie Merlin, Jean-Philippe Merlio, Cécile Meslier, Laurent Mesnard, Sandrine Mestre-Godin, Corinne Metay, Sandrine Meunier, Pierre Meyer, Vincent Meyer, Vincent Michaud, Laurence Michel-Calemard, Cyril Mignot, Marguerite Miguet, Gilles Millat, Tristan Mirault, Albane Miron de l'Espinay, Micheline Misrahi, Clémence Molac, Arnaud Molin, Julie Mondet, Faustine Monin, Pauline Monin, Audrey Monneur, Sophie Monnot, David Montani, Elodie Morel, Godelieve Morel, Valérie Morel, Jessica Moretta, Fanny Morice-Picard, Lucie Morillon, Carole Morin, Marie-Emmanuelle Morin-Meschin, Philippe Morlat, Despina Moshous, Emmanuelle Mouret-Fourme, Alice Moussy, Sébastien Moutton, Kévin Mouzat, Romane Muletier, Jean Muller, Marie Muller, Aleksandra Nadaj-Pakleza, Pierre Naibo, Sophie Nambot, Nadia Nathan, Caroline Nava, Juliette Nectoux, Jeanne Netter, Florent Neumann, Julien Neveu, Zoé Nevière, Laetitia Nguyen, Tanguy Niclass, Gaël Nicolas, Laury Nicolas, Massih Ningarhari, Catherine Nogues, Cécile Novello, Frédérique Nowak, Sylvie Odent, Marie-Françoise Odou, Robert Olaso, Sarah Otmani, Caroline Ovaert, Laurence Pacot, Mélanie Pages, Catherine Paillard, Aurélien Palmyre, Eleni Panagiotakaki, Myriam Pannard, Anne Paoletti, Maria T. Papadopoulou, Matthildi Papathanasiou, Véronique Paquis, Béatrice Parfait, Camille Paris, Clara Paris, Françoise Paris, Éric Pasmant, Marlène Pasquet, Marie Passet, Cédric Pastoret, Olivier Patat, Léa Patay, Antoine Paul, Céline Pebrel-Richard, Cristina Peduto, Regis Peffault de Latour, Antoine Pegat, Annick Pelletier, Valérie Pelletier, Fanny Pellisson, Perrine Pennamen, Alessandra Pennisi, Victor Pereira, Julie Pernin-Grandjean, Julien Péron, Alexandre Perrier, Lionel Perrier, Laurence Perrin, Isabelle Perthus, Arnaud Petit, Audrey Petit, Florence Petit, François Petit, Yuliya Petrov, Hugo Peyre, Christophe Philippe, Juliette Piard, Élise Pierre-Noël, Gaëlle Pierron, Clément Pimouguet, Véronique Pingault, Stéphane Pinson, Emmanuelle Pion, Élise Pisan, Julie Plaisancié, Marc Planes, Pauline Planté-Bordeneuve, William Plas, Morgane Plutino, Ludivine Poignie, Vianney Poinsignon, Marilyne Poirée, Nicolas Pons, Bénédicte Pontier, Valérie Porquet-Bordes, Camille Porteret, Delphine Potier, Louis Potier, Damien Pouessel, Laura Poujade, Marie Preau, Claude Preudhomme, Fabienne Prieur, Vincent Probst, Vincent Procaccio, Caroline Prot-Bertoye, Delphine Prunier, Jacques Puechberty, Mathilde Pujalte, Leila Qebibo, Sylvia Quemener-Redon, Isabelle Quérée, Susana Quijano-Roy, Nicolas Quirin, Caroline Racine, Sandra Raimbault, Judith Raimbourg, Marine Rajaoba, Thomas Rambaud, Carole Ramirez, Francis Ramond, Kara Ranguin, Antonio Rausell, Jean-Marie Ravel, Claudia Ravelli, Gerald Raverot, Isabelle Ray-Coquard, Caroline Raynal, Patricia Réant, Vinciane Rebours, Richard Redon, Yves Réguerre, Philippe Reix, Cécile Renard, Mathilde Renaud, John Rendu, Céline Renoux, Romain Rey, Rachel Reynaud, Lucie Rhinan, Florence Riant, Florence Riccardi, Pascale Richard, Agathe Ricou, Vincent Rigalleau, Marlène Rio, Axelle Rivière, Patrick Robelin, Marion Robert, Thomas Robert, Barbara Rohmer, Pauline Romanet, Arnaud Romoli, Sophie Rondeau, Caroline Rooryck, Bertrand Roquelaure, Jérémie Rosain, Massimiliano Rossi, Sylvie Rossignol, Anya Rothenbuhler, Nadège Rouel, Marine Rouillon, Etienne Rouleau, Anne-Françoise Roux, Nathalie Roux-Buisson, Cécile Rouzier, Emmanuel Roze, Lyse Ruaud, Valentin Ruault, Claire Ruysschaert, Esma Saada, Samira Saadi - Ait El Mkadem, Thoueiba Saandi, Niki Sabour, Sabrina Sacconi, Raphaël Saffroy, Hana Safraou, Virginie Saillour, Aude Saint Pierre, Cécile Saint-Martin, Pierre Saintigny, Gaëlle Salaun, David Salgado, Laurence Salle, Didier Samuel, Florian Sandron, Damien Sanlaville, Laure Sapey-Triomphe, Sabine Sarnacki, Elisabeth Sarrazin, Catherine Sarret, Véronique Satre, Pascale Saugier-Veber, Paul Saultier, Laure Saumet, Claude Scarpelli, Élise Schaefer, Nicolas Scheyer, Isabelle Schiff, Manuel Schiff, Gudrun Schleiermacher, Nicolas Schleinitz, Caroline Schluth-Bolard, Anouck Schneider, Bertrand Schwartz, Jean-Marc Sebaoun, Margaux Serey-Gaut, Hassan Serrier, Aude Servais, Marine Serveaux-Dancer, Nicolas Sevenet, Antoine Seyve, Alicia Sibony-Cohen, Flore Sicre De Fontbrune, Sabine Sigaudy, François Sigaux, Fatoumata Simaga, Victor Simmet, Pauline Simon, Sophie Simon, François Sirveaux, Thomas Smol, Guilhem Solé, Gwendoline Soler, Pauline Solignac, Marie-Hélène Soriani, Isabelle Soubeyran, Jean Soulier, Laurent Spelle, Brian Sperelakis-Beedham, Marco Spinazzi, Marta Spodenkiewicz, Anne Spraul, Barbara Squiban, Arunya Srikaran, Julie Steffann, Anamaria Stetco, Radka Stoeva, Tanya Stojkovic, Dominique Stoppa-Lyonnet, Felipe Suarez, Pierre Sujobert, Juliette Svahn, Minh-Chau Ta, Anne-Claude Tabet, Gaëlle Tachon, Matthias Tallegas, Anne Tallet, Pierre-Edmond Tambourin, Julie Tandonnet, Véronique Tardy, Emmanuelle Tavernier, Dimitri Tchernitchko, Marie-Hélène Teillon-Berranger, Julie Tenenbaum, Jordan Teoli, Marine Tessarech, Benoit Tessoulin, Mylène Tharreau, Christel Thauvin-Robinet, Nathalie Theou-Anton, Julien Thevenon, Anne Thomas, Laure Thomas, Quentin Thomas, Cécile Thomas-Teinturier, Nathalie Tieulie, Julie Tinat, Camille Tlemsani, Sylvie Tondeur, Lucie Tosca, Diego Tosi, David Tougeron, Philippe Touraine, Elisabeth Tournier-Lasserve, Annick Toutain, Frédéric Tran Mau-Them, Christine Tranchant, Olivier Trédan, Aurélien Trimouille, Jean-Noël Trochu, Vincent Tronel, Cécile Trouba, Marie-Élise Truchetet, Nathalène Truffaux, Amel Tsalamlal, Edouard Turlotte, Violette Turon, Maud Tusseau, Nancy Uhrhammer, Christel Vaché, Stéphanie Valence, Thibaud Valentin, René Valero, Sophie Valleix, Marion Vallet, Hélène Vanacker, Pierre Vande-Perre, Yves Vandenbrouck, Clémence Vanlerberghe, Rosa Vargas-Poussou, Camille Vatier, Vincent Vauchel, Dominique Vaur, Lourdes Velo-Suarez, Laurence Venat, Gabriella Vera, Camille Verebi, Célia Verley, Loïc Verlingue, Christophe Verny, Lauren Veronese, Nelly Verotte, Benjamin Verret, Yoann Vial, François Vialard, Alain Viari, Marie Vidailhet, Dominique Vidaud, Michel Vidaud, Stéphane Vignes, Clothilde Vigouroux, Corinne Vigouroux, Laurent Villard, Laurent Villeneuve, Patricia Villié, Marie-Charlotte Villy, Lara Vinauger, Armelle Vinceneux, Anne Vincenot, Christine Vinciguerra, Antonio Vitobello, Patrick Vourc'h, Aurore Vozy, Marie-Laure Vuillaume-Winter, Sandrine Vuillaumier-Barrot, Karim Wahbi, Cédrick Wallet, Thomas Walter, Ulrike Walther-Louvier, Sarah Watson, Anne-Christine Waymel, Sara Weinhard, Nicolas Wiart, Camille Wicker, Marjolaine Willems, Thomas Wirth, Justine Wourms, Antoine Wyrebski, Kévin Yauy, Mohamad Zaidan, Ariane Zaloszyc, Hélène Zattara, Christophe Zawadzki, Alban Ziegler

**Keywords:** Genomic medicine, PFMG2025, French genomic medicine initiative, Rare diseases, Cancer predisposition, Cancers, Genome sequencing

## Abstract

Integrating genomic medicine into healthcare systems is a health policy challenge that requires continuously transferring scientific advances into clinics and ensuring equal access for patients. France was one of the first countries to integrate genome sequencing into clinical practice at a nationwide level, with the ambition to provide more accurate diagnostics and personalized treatments. Since 2016, the French government has invested €239M in the 2025 French Genomic Medicine Initiative (PFMG2025) which has so far focused on patients with rare diseases (RD), cancer genetic predisposition (CGP) and cancers. PFMG2025 has addressed numerous challenges to set up an operational organizational framework. As of December the 31st 2023, 12,737 results were returned to prescribers for RD/CGP patients (median delivery time: 202 days, diagnostic yield: 30.6%) and 3109 for cancer patients (median delivery time: 45 days). PFMG2025’s future priorities encompass ensuring economic sustainability, strengthening links with research, empowering patients and practitioners, and fostering collaborations with European partners.

**Funding:**

As of December the 31st 2023, €239M have been invested by the French government.

## Introduction

Several genomic characterization programs at a population level, funded either exclusively through public grants or a mix of public and private funding, were set up from the 2010s with the aim of identifying the genetic determinants of human diseases by sequencing healthy participants or clinical cohorts.^1^, ^2^, ^3^, ^4^, ^5^ Although most of these programs considered the integration of personalized medicine into their healthcare system as a goal, only a minority of them have achieved this so far (UK, Sweden, Denmark).^2^ The clinical aims are to provide more accurate and timely diagnostics, strengthen prevention and improve patient outcome by the development of targeted treatments. In 2015, the French National Alliance for Life Sciences and Health (Aviesan) was commissioned by the French government to launch in 2016 the 2025 French Genomic Medicine Initiative (*Plan France Médecine Génomique 2025–PFMG2025*). The ambitions of this initiative were to integrate genomic medicine into the healthcare system within a research-care continuum, by ensuring the transfer of scientific advances to the clinic, and to provide fair access to innovation for all patients nationwide.^6^ Whereas several national genomics programs were initially based on large translational research programs with secondary transfer to patient care, its original approach was to directly implement genomic medicine in clinical practice and to make healthcare data available for research purposes. *PFMG2025* revolved therefore around four main objectives: (i) implementing genome sequencing (GS) in clinical practice, (ii) providing therapeutic benefits for patients through a comprehensive exploration of diseases, (iii) developing the capacity to handle massive datasets in the routine and research settings, and (iv) addressing ethical and socio-economic challenges. France has opted for GS rather than exome sequencing (ES) because it is a more comprehensive approach in the clinical as well as in the research setting, and gradual reduction in costs has made it more affordable. This initiative encompassed specific infrastructures: (i) a reference center for innovation, assessment, and transfer (*CRefIX*); (ii) a network of GS clinical laboratories (*FMGlabs*) and prescribers capable of phenotyping, sampling, sequencing, providing clinical interpretation and returning results of thousands of genomes per year; and (iii) a national facility for secure data storage and intensive calculation (*Collecteur Analyseur de Données–CAD*). The first years of the initiative focused on patients with rare diseases/cancer genetic predisposition (RD/CGP) and cancers (liquid and solid tumors), with the project of expanding to more common diseases, such as complex multifactorial diseases.

In the present article, we outline the proactive planning and implementation of GS in clinical practice highlighting the key elements of feasibility and accessibility of such a national initiative for French citizens, as well as the timelines of their achievement, and we present the main deliverables and the expected upcoming challenges.

## Establishing the framework for genomic medicine implementation

**Fig. 1:**
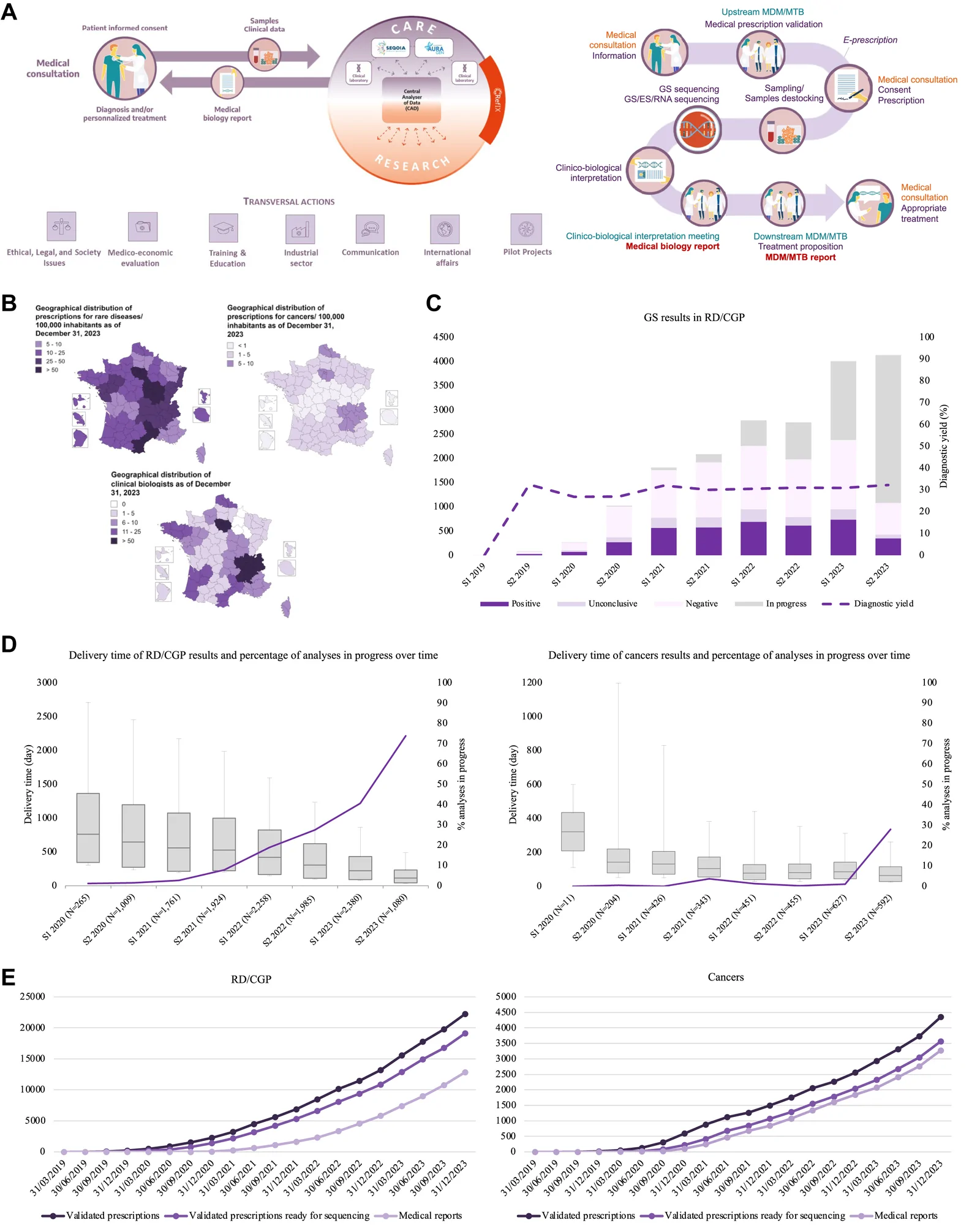
**Major hallmarks of the framework for genomic medicine in France.** (A) *PFMG2025* organization: Overview of the *PFMG2025* initiative in a research-care continuum (left). The interactions between the 3 main specific infrastructures are illustrated, with diagnostic reports sent from the two first *FMGlabs* (AURAGEN and SeqOIA) to patients for diagnosis and/or personalized treatment, data transfer to the national facility for secure data storage and intensive calculation (*Collecteur Analyseur de Données–CAD*) for research with the technical support of the reference center for innovation, assessment, and transfer (*CRefIX*). Several working groups dedicated to ethical, legal and society issues, medico-economic evaluation, training and education, industries, communication, international affairs were set up. Four pilot research projects were launched, in research settings. The genomic healthcare pathway from prescription to delivery of the result to the patient (right). The genomic healthcare pathway has various successive stages: an initial medical consultation to inform the patient, an upstream multidisciplinary meeting (MDM) for rare diseases and cancer genetic predisposition (RD/CGP) or multidisciplinary tumor board (MTB) for oncology to validate the medical prescription, a medical consultation to collect the patient’s consent and to perform an electronic prescription, sample preparation and dispatch to *FMGlabs*, exome/genome/transcriptome sequencing, bioinformatics analysis, clinico-biological interpretation with the drafting of the report sent to the prescribers, a medical consultation to report the results to the patient. As an option, a clinico-biological interpretation support meeting and/or a downstream MDM can be set up to discuss complex cases before drawing-up the diagnostic report and/or the treatment proposal, respectively. (B) Geographical distribution of prescriptions dated 12/31/2023/100,000 inhabitants for RD/CGP (top left) and for cancers (top right), as well as geographical distribution of biologists for RD/CGP and cancers dated 12/31/2023 (bottom). (C) Genome sequencing (GS) results in RD/CGP: histogram showing the number of “complete” RD/CGP prescriptions per semester, with a breakdown of positive diagnoses (purple), inconclusive results (mauve), negative results (light mauve) and analyses in progress (grey), as well as the diagnostic yield (purple dotted line) with little change between 31.6% for the prescriptions performed in 2021 (94.6% completeness), 30.8% for the prescriptions performed in 2022 (76.8% completeness), and 31.3% for the prescriptions performed in 2023 (42.5% completeness). (D) Delivery time between receiving the prescription at *FMGlabs* and returning the report to the prescribers (grey box), as well as percentage of analyses in progress (purple line), by semester, from 01/31/2020 to 12/31/2023: progressive decrease in the median time between the 1st semester of 2020 and the 2nd semester of 2023 for the 12,737 returned results in RD/CGP (left) and for the 3109 returned results in cancers (right). (E) Prescription and medical reporting activities from 03/31/2019 to 12/31/2023 for RD/CGP (left), with genomic prescriptions validated in MDM (dark purple), “complete” prescriptions with samples received by *FMGlabs* (purple) and medical reports (mauve), and for cancers (right), with genomic prescriptions validated in MTB (dark purple), “complete” prescriptions with samples received by *FMGlabs* (purple) and medical reports (mauve).

To successfully carry out *PFMG2025*, notably the provision of nationwide access to genomic medicine in a research-care continuum, the French government invested massively in setting up high-performance facilities, in the development of specific tools and in drafting guidelines (Fig. 1A, Appendix p6). The project was coordinated by working groups based on a strong national framework, structured for many years in the fields of RD, CGP and oncology (Appendix p7), and composed of experts in genetic diagnostics, ethics, legal affairs, policy makers, representatives of national health and research institutions, and patients’ associations. Furthermore, four pilot projects were launched within the framework of the initiative (Appendix p8).

The Ministry of Health (MoH) launched a national call for projects to create the first two *FMGlabs* for clinical GS with possible public-private partnerships, while the French Health Technology Assessment Agency (*Haute Autorité de Santé–HAS*) required that a genomic analysis be prescribed according to well-defined clinical criteria selected through successive calls for proposals open to health professionals. These clinical ‘pre-indications’ are subjected to a medico-economic analysis to determine which will eventually be covered by the French Health Insurance System and become ‘clinical indications’. To ensure patient access to genomic medicine, a multidisciplinary genomic healthcare pathway was first structured with several stages, in particular the introduction of new e-prescription softwares,^7^ and the setting-up of upstream and downstream multidisciplinary meetings (MDM) for RD/CGP or multidisciplinary tumor boards (MTB) for cancers (Fig. 1A, Appendix pp9-10). Based on initial feedback, additional specific measures were taken to facilitate genomic healthcare pathways, from patient information to clinical reports to the prescribers. In parallel, information sheets and consent forms were drafted, and the organization had to comply with French legal constraints on genetic diagnosis (Appendix p11). Secondary use of data for research was included in the consent forms in compliance with General Data Protection Regulation (GDPR).

For individuals with well-defined clinical criteria of a ‘pre-indication’ validated by upstream MDM/MTB, *PFMG2025* proposed two main settings and associated strategies, (i) germline analyses in RD and CGP (RD/CGP) and (ii) tumoral analyses in cancers. For RD/CGP, short-read GS was proposed, preferably including the sequencing of the proband with other family members (trio-based or duo-based with an unaffected related was favored). For cancers, GS, ES and RNAseq were proposed from frozen patient tumor tissues in addition to germline GS with the aim of detecting actionable somatic variants.

## Retrospective study of the 18,926 consecutive prescriptions in RD/CGP and 3367 in cancers as of 12/31/2023

Data available in *FMGlabs’* information systems on 12/31/2023, including prescription date, report date, diagnosis status, and delivery time, were extracted. The delivery time corresponded to the timelapse between reception and validation for sequencing of the ‘complete’ prescription file (i.e., with samples and consent forms) by the *FMGlab* to the moment the diagnostic report was sent to the prescriber. For RD/CGP, information regarding how the analyses were conducted in the proband’s family (number of individuals concomitantly sequenced), results of previous tests (standard chromosomal analysis, array comparative genomic hybridization, single gene testing, panel, and ES) and the details of identified variants were collected from 04/01/2019 to 06/30/2021. For cancers, the tumoral primary location and histotype, and the details of identified variants were collected from 04/01/2019 to 06/30/2022.

## Successful implementation of the national genomic medicine network

The MoH selected two laureates from 12 proposals for the creation of *FMGlabs* for clinical GS. *CRefIX* and *FMGlabs* agreed on common protocols (Appendix pp12-16). Seventy pre-indications (62 for RD/CGP and 8 for cancers) were selected, with 17,380 prescriptions *a priori* estimated annually for RD/CGP patients (17,230 for RD and 150 for CGP), and 12,300 for cancers (Appendix pp17-19) according to estimates provided by pre-indications’ referents. National guidelines were drawn to ensure optimal prescriptions and standardize medical practices. For each pre-indication, a flowchart was designed, defining the eligibility criteria for GS, together with required preliminary tests (URL). In addition to informed consent forms, 16 information sheets for different levels of understanding were written and translated into several languages (Appendix p11).

*FMGlabs* processed prescriptions from two territories with an equivalent population. In total, 120 thematic upstream MDMs and 26 MTBs were created (Appendix pp9-10). As this organization quickly proved to be time-consuming in RD, a national network of 24 local non-thematic MDMs, coordinated by clinical geneticists, was subsequently created. In 2023, local non-thematic MDMs were widely used to validate 48.3% of prescriptions. Overall, 71.4% of prescriptions were validated by local MDMs, either thematic or non-thematic, and 28.6% of the prescriptions by the 120 thematic non-local MDMs.

To date, 1823 clinicians from all over the country have gradually created their prescriber account, 1161 (63.7%) have made at least one prescription and 75/1161 (6.5%) have been responsible for 69.4% and 42.4% of the prescriptions for RD/CGP and cancers, respectively. In order to increase prescriptions, *PFMG2025* created a network of 51 new health professionals (referred to as genomic pathway managers) to assist and monitor genomic prescriptions, and to train prescribers to use electronic prescription tools. GS prescriptions for RD/CGP were progressively conducted throughout the territory, while they remained concentrated in a few regions for cancer patients (Fig. 1B; Appendix p20). For both *FMGlabs*, sequencing was performed on site and bioinformatics analyses were conducted with their local teams.

For clinical interpretation of variants, *FMGlabs* called on experts from any French public hospital or cancer center, who were involved in *PFMG2025* through a partnership agreement drafted by the MoH to ensure compliance with French laws on medical biology. To date, biological interpretation is carried out by 310 clinical biologists (molecular geneticists or biologists) across the country (Fig. 1B): 21/310 (6.8%) wrote 54.6% and 40.4% of the reports for RD/CGP and cancers, respectively. Based on this organization, the number of prescriptions and genetic testing reports has steadily increased since 2019 (Fig. 1E).

As of December the 31st 2023, €239M have been invested by the government.

## A causal diagnosis reached in 30.6% of patients with RD/CGP

As of December the 31st 2023, 22,259 prescriptions electronically validated in the e-prescription tools after MDM were filled in all pre-indications (MDM had so confirmed that the prescriptions met the eligibility criteria). Among these, *FMGlabs* received a total of 18,926 (85%) ‘complete’ prescription files for deceased fetuses, children or adults. This number increased slowly from 2019 onwards and accelerated after 2021, rising from 3896 in 2021 to 8136 in 2023 (46.8% of expected annual complete prescriptions in 2023). In 2020, the increase was partially curbed by the Covid-19 epidemic, which led to a complete lockdown period in France from 03/17/2020 to 05/11/2020, with the closure of a number of clinical centers during this period, and of both *FMGlabs* from the 17th of March to early June 2020. Malformations and neurodevelopmental (MND) disorders were by far the most represented subgroup of pre-indications, composed of pre-indications with some overlapping clinical characteristics (65.5%; 12,399/18,926), followed by sensory disorders (8.9%; 1684/18,926), central nervous system (CNS) disorders (6.5%; 1236/18,926) and bone and joint diseases (4.4%; 831/18,926). MND disorders were nevertheless underrepresented because patients with intellectual disability were included in the DEFIDIAG protocol until the last inclusion in 2022 (Appendix p8). In 2023, 8136 ‘complete’ prescriptions were received, representing 46.8% of the 17,380 expected annually, with variations from one subgroup of pre-indications to another, with some exceeding expectations (131% for chronic kidney disease and 115.2% for neuromuscular diseases) and others still well below it (3.4% for fertility disorders and 11.4% for diabetes).

A total of 12,737 results were returned to prescribers, resulting in a completeness rate of 67.3% (number of results returned to prescribers among the 18,926 ‘complete’ prescription files) as of December the 31st 2023 (Fig. 1E; Appendix pp17-18,21–24). The number of results returned to the prescribers increased notably between 4200 in 2022 and 6890 in 2023 (+64%). The completeness rate appeared highly variable according to the subgroups of pre-indications (from 37.8% in rare lung diseases, 40.7% in endocrine disorders, to 88% in hematological diseases, and 95% in diabetes, p < 10^−3^) and was not correlated to the size of the subgroup of pre-indications (p = 0.702). The 6189 remaining ‘complete’ prescription files were awaiting a final report on 12/31/2023 (most of them sequenced but awaiting interpretation), with 79.7% belonging to 3 large subgroup requests (MND disorders, CNS disorders and sensory disorders).

Despite the large increase in complete prescriptions, the median delivery time between receiving the complete file at *FMGlabs* and returning the report to the prescribers (202 days with 67.3% of completeness) notably decreased over time (Fig. 1D), from 348 days in the 1st semester of 2021 (97.4% of completeness) to 73 days in the 2nd semester of 2023 (26.1% of completeness). However, this median of 73 days is provisional and underestimated since the completion rate is only 26% for this period. As of 12/31/2023 it also appears to be highly variable according to the subgroups of pre-indications and was not correlated with their size: from 163 days in MND disorders (69% of completeness), 182 days in cardiac diseases (79.3% of completeness) and 209 in neuromuscular diseases (45.1% of completeness) to 370 days in sensory disorders (59.5% of completeness), 408 in immunological and autoinflammatory diseases and 427 days in chronic kidney diseases (59.4% of completeness).

**Fig. 2:**
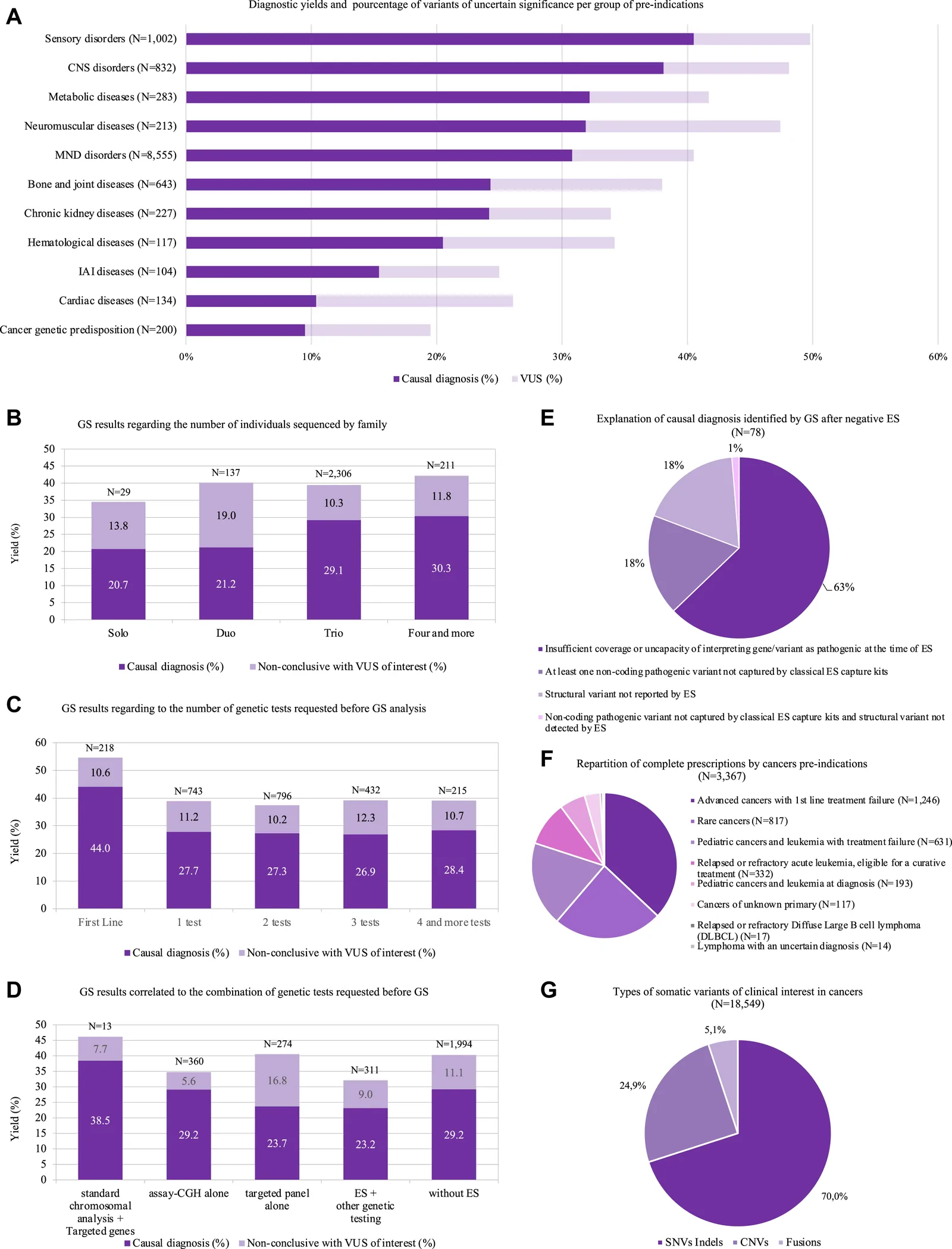
**Diagnostic yield in patients with rare diseases and cancer genetics (RD/CGP) and somatic variants in cancers.** (A) Diagnostic yields (purple) and VUS levels (mauve) presented per subgroup of RD/CGP pre-indications, after exclusion of subgroups with less than 100 patients; (CNS: central nervous system disorders, MND: malformations and/or neurodevelopmental disorders, IAI: immunological and autoinflammatory diseases). (B) Retrospective study of the first 2734 consecutive prescriptions for RD/CGP: Diagnostic yields (purple) and VUS levels (mauve) regarding the number of individuals sequenced in a family (solo, duo, trio and four and more). (C) Retrospective study of the first 2734 consecutive prescriptions for RD/CGP: Diagnostic yields (purple) and VUS levels (mauve) regarding the number of genetic tests requested before GS analysis (first-line, one, two, three and four and more). (D) Retrospective study of the first 2446/2734 consecutive prescriptions for RD/CGP: Diagnostic yields (purple) and VUS levels (mauve) after different combinations of genetic tests requested before GS analysis (standard chromosomal analysis and targeted genes, array comparative genomic hybridization (array-CGH) alone, targeted gene panel alone, exome sequencing with other genetic tests, and all other genetic tests without exome). (E) Retrospective study of the first 2734 consecutive prescriptions for RD/CGP: reasons why GS identified a causal diagnosis in 78 patients with negative ES. (F) Repartition of the 3367 complete prescriptions by cancers pre-indication from 04/01/2019 to 12/31/2023. (G) Types of the 18,549 somatic variants returned for discussing actionability and treatment proposition in the MTB identified in 1718 patients.

Overall, a causal diagnosis was reached in 3895/12,737 patients (30.6%) with little change over time, even in 2023 with a completeness rate of 42.5% (Fig. 1C). This was largely influenced by the diagnostic yield of MND disorders (30.8%), which represents 8555/12,737 (62.2%) of the results returned to prescribers. Indeed, it appeared highly variable across subgroups of pre-indications. The largest diagnostic yields were observed in rare skin disorders (46.3%), sensory disorders (40.5%), and CNS disorders (38.1%) (Fig. 2A). The diagnostic yield of MND disorders (30.8%) was probably underestimated since intellectual disability was underrepresented because of the DEFIDIAG study (Appendix p8).

Overall, a non-conclusive diagnosis with a variant of uncertain significance (VUS) was reached in 1289/12,737 (10.1%) patients.

**Fig. 3:**
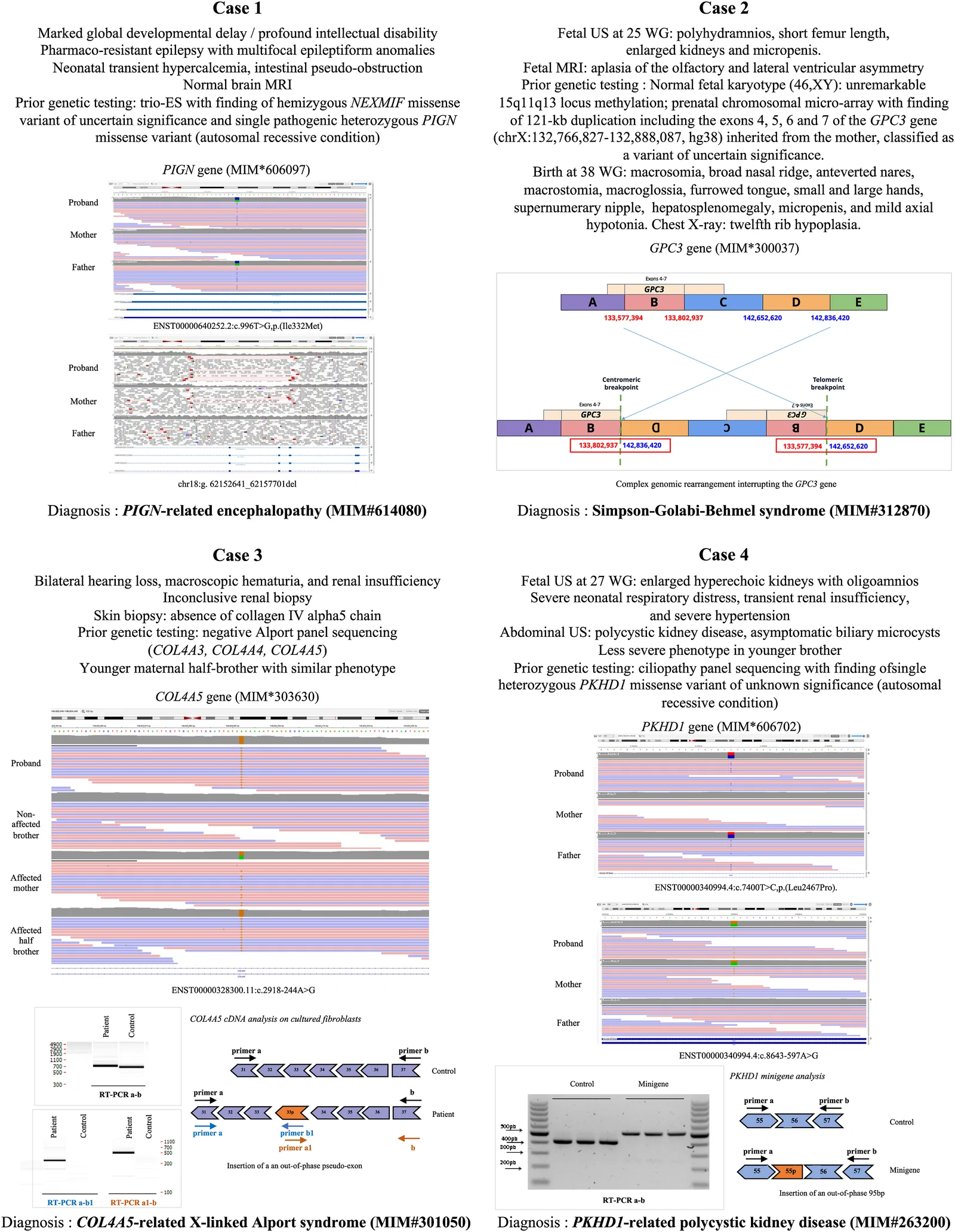
**Four clinical cases of interest in RD diagnosed by GS.** GS identified causative variant after a normal gene panel sequencing (Case 3) or after a heterozygous variant of unknown significance in genes with autosomal recessive condition identified by gene panel sequencing (Case 4) or exome sequencing (Case 1). GS also characterized a structural variant of unknown significance previously detected by array-CGH leading to its reclassification in causative variant (Case 2). Case 1: a diagnosis of *PIGN*-related encephalopathy (MIM#614080) secondary to compound heterozygous variants (missense and intragenic deletion) in *PIGN* (MIM∗606097); main clinical features and IGV capture of both variants in proband and unaffected parents. Case 2: a diagnosis of Simpson-Golabi-Behmel syndrome (MIM#312870) secondary to complex genomic rearrangement interrupting the *GPC3* gene (MIM∗300037); main clinical features and diagram explaining the rearrangement. Case 3: a diagnosis of X-linked Alport syndrome (MIM#301050) secondary to inherited deep intronic variant in *COL4A5* (MIM∗303630); main clinical features, IGV capture of the variant in proband, non-affected brother, affected mother, and affected half-brother, and RT-PCR results on patient cultured fibroblasts compared with control fibroblasts, revealed the formation of an out-of-phase pseudo-exon, using the splice acceptor site enhanced by the variant and the strongest of the preexisting donor sites with a total effect on splicing. Arrows indicate the primers used for RT-PCR located in exons 31 (primer a) and 37 (primer B), as well as in the inserted 33p predicted pseudo-exon (primers a1 and b1). Case 4: a diagnosis of autosomal recessive polycystic kidney disease (MIM#263200) secondary to heterozygous composite variants (missense and deep intronic variant) in *PKHD1* (MIM∗606702); main clinical features and IGV capture of both variants in proband and unaffected parents (ES: exome sequencing, MIM: Mendelian Inheritance in Man, MRI: Magnetic Resonance Imaging; US: ultrasound, WG: weeks of gestation, RT-PCR: Reverse Transcription Polymerase Chain Reaction).

For the first 2734 GS prescriptions, the diagnostic yield was higher in trio (29.1%) than solo (20.7%), although this difference was not statistically significant (p = 0.310) and the percentage of VUS returned to prescribers (10.3%–19%) was not correlated with the number of individuals sequenced in the family (p = 0.015) (Fig. 2B). For the first 2446/2734 GS prescriptions (89.5%) for which an analysis of the diagnostic strategy was carried out, GS was proposed at diverse time points in the clinical diagnostic pathway, from a first-line test (9.1%) to a novel test following years of diagnostic odyssey (27.1% with more than three negative genetic tests). Among the 2224 probands for which GS was not a first-tier genetic assessment, patients were previously assessed by standard chromosomal analysis (31.3%) array-CGH (69.4%), single gene sequencing (40%) targeted gene panels (52.3%) and/or exome sequencing (15.3%) (Fig. 3). A causal diagnosis was significantly more frequently reached when GS was used as a first-line diagnostic test (44%) rather than after one or multiple genetic tests (27.4%) (p < 10^−3^) (Fig. 2C). The diagnostic yield of GS appeared to be higher (29.2%) in patients with normal array-CGH than in patients with a negative targeted gene panel alone (23.7%) or with exome sequencing with other genetic testing (23.2%) (p = 0.143) (Fig. 2D).

Among the 340 patients with negative ES results, GS identified a causal diagnosis in 78/330 patients (23.6%) (97.1% of completeness). For 49/78 patients (62.8%), causative variants were in the coding regions, but ES failed in identifying the causal variant because of either insufficient coverage or the gene/variant could not be interpreted as pathogenic at the time of ES (i.e., unrelated to OMIM rare diseases at the time of ES). Interestingly, for 29/78 patients, GS input was critical to reach a final diagnosis in patients with at least one non-coding pathogenic variant not captured by classical exome capture kits (18%) or with a structural variant not reported by ES (18%), or both together (1%) (Fig. 2E, Appendix pp27-30). Among the 15/78 patients (19.2%) with a structural variant not detected by ES, 11/15 had previously benefited from array comparative genomic hybridization (array-CGH) with normal results (8/11) or with VUS results (3/11).

## Identification of somatic variants returned for discussion in MTB in ∼90% of the prescriptions for cancer patients

As of December the 31st 2023, 4351 prescriptions were electronically validated after MTB in all sub-groups of pre-indications (MTB had so confirmed that the prescriptions met the eligibility criteria). Among these, *FMGlabs* received 3367 (77.4%) ‘complete’ prescription files in cancers for affected children or adults with a strong increase between 913 in 2022 and 1456 in 2023 (+59.5%). The number of complete prescriptions varied from one pre-indication to another. The most common pre-indications were advanced adult cancers with first-line treatment failure (37%), rare cancers (24.3%), and pediatric cancers and leukemia with treatment failure (18.7%) (Fig. 2F, Appendix pp19). In 2023, 1456 ‘complete’ prescriptions were received, well below the 12,300 expected annually (11.8%), except for the pre-indication relapsed or refractory acute leukemia, eligible for a curative treatment (Appendix p24).

A total of 3109 results were returned to the prescribers, resulting in a completeness rate of 92.3% on 12/31/2023 (Fig. 1E). This number increased significantly between 921 in 2022 and 1339 in 2023 (+45.4%). The completeness rate was constantly very high (96.3–100%) with few results in progress, except for the second semester of 2023 (72%) because of the delivery time. The median delivery time (45 days with 92.3% of completeness) notably decreased, from 61 days in the 1st semester of 2021 (100% of completeness) to 35 days in the 2nd semester of 2023 (72% of completeness). It also appeared highly variable according to the pre-indications (from 31 days in relapsed or refractory acute leukemia, eligible for a curative treatment to 80 in relapsed or refractory Diffuse Large B cell lymphoma (DLBCL), and 91 in lymphoma with an uncertain diagnosis) (p < 10^−3^) (Appendix p24).

For the first 1974 GS prescriptions in cancers, 1940 requests were complete resulting in 1945 GS, 1609 ES and 1534 RNAseq of frozen tumoral tissues performed in comparison to 1953 GS of normal tissues. For those tumors where the primary location was clearly indicated (80.6%), the most represented tumor topographies were tumors of the brain or eye (24.5%), digestive tract (12.4%), bone (10.3%), breast (9.1%), and female genital organs (7.2%), followed by blood malignancies (6.3%) and respiratory system and intrathoracic organs (6.3%). For those tumors where the morphological characteristics were clearly indicated (88.8%), the most represented were adenocarcinoma (33.1%), glioblastoma (5.5%), other glioma and sarcoma (17.5%), as well as leukemia (5.5%) (Appendix pp30-31).

For 1718/1940 patients (88.6%), 18,549 somatic variants of interest were returned in order to discuss actionability and treatment proposition in the downstream MTB (12,991 SNVs/Indels, 4613 CNVs and 945 gene fusions) (Fig. 2G). SNV/Indels, CNV and gene fusions of interest were reported to the prescribers in 90.2%, 62.1% and 25.7% of patients, respectively. The precise description of the landscape of somatic gene alterations goes beyond the present article.

Briefly, six genes with SNVs/Indels of interest e.g., inactivation of tumor suppressor genes (TSG) (*TP53*, *ATRX*, *NF1*) or activation of a proto-oncogene (*TERT*, *PIK3CA*, *KRAS*, …), and 8 genes with CNVs were detected in more than 5% of tumors analyzed (Appendix pp32-33). For CNVs (amplification of oncogenes or deletions of TSG), the most common large deletions involved 5 genes (*CDKN2A*, *CDKN2B*, *TP53*, *MTAP*, *RB1*) and the most common amplified gene was *EGFR*, a common proto-oncogene. A large diversity of fusion oncogenes was observed, primarily in one tumor, the three most common being: EWSR1::Fli1 (n = 13) observed in Ewing sarcomas, PAX3::FOXO1 (n = 12) in rhabdomyosarcomas, and ETV6::RUNX1 (n = 8) in childhood leukemias.

In 1589 samples where tumor mutational burden (TMB) was calculated, 31.7% patients had a TMB ranging from 0 to 1 mut/Mb and 4.3% had a TMB>10 mut/Mb, potentially qualifying for a treatment with immune checkpoint inhibitors. Actionable germline pathogenic variants of TSG, such as BRCA1/2 or TP53, were observed in 115/1718 (6.7%) patients.

## Discussion

France was one of the first countries to integrate GS directly into the healthcare system at a nationwide level, for its 67 million inhabitants.^6^ The implementation of a long-term national genomic medicine initiative raised major challenges to concomitantly ensure equal access to genomic analyses, medical benefits for patients, and economic sustainability.^7^

Equal access was promoted by providing GS free of charge for patients, guarantying nationwide coverage by dividing the mainland and overseas areas between *FMGlabs*, and deploying process standardization. This included the definition of a common genomic healthcare pathway and the diagnostic strategy for each pre-indication.^8^ Laboratory protocols followed recommendations made by *CrefIX* and international best practices, together with the drafting of common models of reports by clinical biologists. This strong but complex organization led to a slow implementation during the first three years. It was reinforced in 2020–2022 by the integration of the pre-indication with the largest RD patient group, namely ‘Intellectual disability’, the addition of which was delayed because patients were first included in the DEFIDIAG pilot project, and because of the Covid-19 pandemic, which led to several lockdowns with closures of clinical departments and *FMGlabs*. Specific actions were set up to facilitate the genomic testing pathway, such as the deployment of 24 local non-thematic MDMs in RD and the creation of a new dedicated function (genomic pathway managers). These actions notably improved the number of prescriptions between 2022 and 2023 (+47.3% for RD/CGP and +55.8% for cancers). Nevertheless, *FMGlabs* received GS prescriptions for only 11.8% of the annually expected cancer patients in 2023. Over a 4-year period, the tumors of around 3500 patients were analyzed, while 420,000 new patients are diagnosed with cancer each year, about half of whom will relapse after surgery and require medical treatment. It would be useful to rally clinicians working throughout the French territory, by removing obstacles linked to the use of frozen samples (cancer samples are frozen in <20% of patients nationwide, with strong disparities across territories and structures). *CRefIX* and *FMGlabs* evaluated the best biological and bioinformatics practices to optimize DNA and RNA processing from formalin-fixed paraffin-embedded tumor samples (FFPE) to maximize the number of eligible cancer patients. From November 2023, the analysis of FFPE biopsies is being progressively deployed in *PFMG2025* with regular evaluation. In the field of RD, the American College of Medical Genetics and Genomics (ACMG) recommended that GS should be used as a first- or second-tier test for patients with congenital and/or intellectual disability, who represent 1–2% of the population.^9^ Much remains to be done to scale-up this testing. The first challenge will be to increase the current number of 818 prescribers in RD/CGP and/or their prescription rates. Although clinical geneticists are the main prescribers for RD (71.4% of the prescriptions validated by local MDMs composed mainly of clinical geneticists), other types of clinicians need to be trained to integrate this approach into their practice.^10^ Indeed, with GS becoming accessible for multiple diseases in France, there is a need to improve the training of more medical doctors in genetics/genomics, as the management of prescription, data analysis, and delivery to patients still relies mainly on a small number of medical doctors with a specific training and experience in genetics. In addition, prescribing procedures could be further simplified by calling upon the MDMs exclusively for complex situations. However, it could only be envisioned as the time as the medico-economic evaluation of the pre-indications, as these MDMs guarantee rigorous compliance with the prescribing criteria required for this up-coming evaluation. *PFMG2025* was initially supported by substantial national resources (€239 millions as the end of 2023), mostly invested by the MoH before considering the reimbursement by the healthcare system after a national evaluation conducted by the *HAS*. After setting up *FMGlabs*, the MoH funded a medico-economic research program in order to provide a proof-of-concept of the cost-effectiveness of GS in healthcare. This issue must be addressed by other national genomic medical initiatives and constitutes an avenue for international collaborations.

For RD/CGP, GS prescription in clinical practices critically enhanced our national diagnostic capacities for returning clinically significant results to the families. For the first 2734 GS, *PFMG2025* exhibited a positive diagnostic yield of 28.7%, which is slightly higher than those observed in the 100,000 Genomes project (25.0%), likely due to a higher proportion of trios (85.5% vs 44%) and different preliminary genetic testing.^11^ It could be considered lower than expected with a trio approach,^12^ but most patients experienced a diagnostic odyssey with multiple preliminary genetic tests. Indeed, the diagnostic yield was significantly higher as a first-line diagnostic test (44%) than as at least a second-line genetic test (27.4%). Interestingly, GS identified a causal diagnosis in 23.6% of patients with a previous negative ES. GS has the advantage over ES of identifying structural and non-exonic variations, as recently demonstrated by the identification of sporadic variants in the non-coding spliceosomal snRNA gene *RNU4-2* as a frequent cause of syndromic neurodevelopmental disorders.^13^ As expected, the overall diagnostic yield (30.6% with 67.3% of completeness) also varied according to pre-indication subgroups (from 46.3% in rare skin disorders to 9.5% in CGP), reflecting not only differences in the proportions of genetic diagnoses between subgroups but also a large heterogeneity in clinical practices. Of note, 59.3% of the results were negative and 10.1% of them were non-conclusive with VUS. Various measures are essential to improve diagnostic yields, such as developing functional assays to classify VUS, performing data reinterpretation in a clinical setting at regular time intervals (every 2 years according to ACMG recommendations),^14^ strengthening efforts to better represent the genetic diversity of the population living in France in databases, allowing secondary use of patient data for research purposes and sharing data both at national and international levels (Appendix p34).^15^^,^^16^ For CGP, the forthcoming genomic characterization of the tumors and the polygenic score risk approaches should optimize the results.

For cancer patients, the identification of tumor genomic alterations, with oncogenic properties can serve as biomarkers to identify candidate patients for innovative therapies.^17^^,^^18^ Similarly, loss of tumor suppressor genes or defects in the mismatch repair gene pathways are now used to guide treatment decision in the first-line setting.^19^ *PFMG2025* demonstrates that GS/ES/RNAseq can be provided for this purpose on a nationwide basis, with at least one somatic variant of interest reported to the MTB to discuss actionability and treatment proposition in 88.6% of patients, albeit the level of improvement in treatment options is outside the scope of this article and will be reported in the future. GS/ES/RNAseq also provided information for patient diagnosis (e.g., for cancers of unknown primary origin and for genetic subtyping of cancers). Actionable germline pathogenic variants of TSG were reported in 6.7% of patients. In the future, it will be important to select candidate patients for such molecular characterizations based on the benefits for their healthcare pathway. It is likely that gene panels will be sufficient in daily practice for most patients, while for a subset, GS/ES/RNAseq will allow refinement of cancer classification and guide treatment. Moreover, ensuring a fair access of cancer patients to genomic-driven approved or experimental therapies is essential in order to optimize the clinical impact of GS/ES/RNAseq.

*PFMG2025* was set up to meet the needs of patients regardless of their age. Many prescriptions were for children as many RD manifest during childhood, meaning that the main pre-indications (intellectual disability and developmental abnormalities, malformation syndromes and dysmorphic syndromes without intellectual disability) are more abundant in children, and two cancer pre-indications mostly affect children (pediatric cancers and leukemia at diagnosis, and pediatric cancers and leukemia with treatment failure). This required adapting the consent forms to minors. Moreover, the two initial information sheets for RD/CGP and cancer patients were released in three versions for minors (classic, simplified and illustrated) and two for their parents (simplified and illustrated), according to their level of understanding.

One of the immediate challenges is to reduce the delivery time and increase the completeness (only 67.3% of the around 18,900 RD/CGP patients had received a diagnostic report on 12/31/2023).^20^^,^^21^ The delivery time for cancers (35 days in the 2nd semester of 2023 with 72% of completeness) was much shorter than for RD/CGP but still needs to be improved. The development of analytical tools for prioritizing variants, and a variant-centered database will improve the overall clinical and biological interpretation capacity, not only for RD/CGP patients but also for cancer patients.^22^ Such a knowledge database (*FMG-kb*) is being implemented within *CAD*. *FMGlabs* may also request the contribution of any qualified clinical biologists for data interpretation, leveraging their expertise. Nevertheless, 54.6% and 40.4% of reports for RD/CGP and cancers were made by only 6.8% of clinical biologists, indicating a need to refocus activities on GS rather on gene panels with a low diagnostic yield. The MoH has instructed the *HAS* to evaluate the cost-efficacy of all the gene panels used in France to regulate their use. Furthermore, the number of clinical biologists could be increased by revising the accreditation criteria, which are currently legally limited to medical doctors and pharmacists in France.

Many more challenges remain to be addressed: managing incidental findings considering the recent revision of the bioethics laws,^23^ assessing economic sustainability,^24^ anticipating the development of genome wide polygenic scores and scaling up to a larger spectrum of diseases. Genomic medicine is evolving rapidly in an international context, with the development of multi-omics technologies, paving the way for new applications,^25^^,^^26^ the drastic reduction of GS costs,^27^ its large-scale expansion^28^ such as in newborn screening,^29^ and the development of an increasing number of innovative personalized therapies. The current *FMGlabs*’s capacity will soon prove to be insufficient, and authorities are encouraged to take measures in the near future to increase the current capacity for genomic analysis in France. Concomitantly, a major effort is also required to improve the genomic-related health literacy and engagement of citizens.^30^

Seven years after its launch, *PFMG2025* has successfully integrated GS into the French healthcare system. Our national program has overcome numerous challenges to establish genomic medicine as a fair and sustainable service for the population. Our work emphasizes the critical need for precise coordination between healthcare and research institutions, engaging citizens, health professionals, researchers, policy makers and specialized industry. Furthermore, the alignment of multiple national genomic medicine initiatives across Europe into a collaborative public health initiative is poised to transform medical practice in the coming years, with *PFMG2025* playing a key role.

## Contributors

P. Blanc, J.Y. Blay, Y. Duffourd, F. Nowak, C. Thauvin-Robinet, J. Thevenon: data curation and formal analysis.

Y. Duffourd, C. Thauvin-Robinet: validation.

C. Binquet: methodology.

G. Nicolas, F. Nowak, C. Thauvin-Robinet, J. Thevenon: writing–original draft.

C. Binquet, P. Blanc, J.Y. Blay, C. Boileau, T. Bourgeron, P.J. Bousquet, E. Clappier, J.F. Deleuze, P. Laurent-Puig, F. Lethimonnier, S. Lyonnet, G. Nicolas, F. Nowak, S. Odent, P. Saintigny, F. Sigaux, D. Stoppa-Lyonnet, P. Sujobert, C. Thauvin-Robinet, J. Thevenon, M. Vidaud, L. Vinauger, C. Vinciguerra: writing-review & editing.

All contributors were involved in conceptualization, project administration, supervision, investigation, resources, software and/or funding acquisition.

## Data sharing statement

The molecular datasets presented in this study can be found in online repositories on the website: https://pfmg2025.fr/.

## Declaration of interests

*PFMG2025* leadership declare no conflict of interest. J. Y Blay has relationships with the INCa, the EU commission and the French National Research Agency (ANR) and has received research grant for the clinical trial Profiler 2 (not related) from the Roche company. P. Saintigny and has received grant and equipment, materials, drugs, medical writing, gifts or other services from the Roche, Roche Molecular Diagnostics, Astrazeneca, Novartis, Bristol Myer Squibb, Illumina, HTG Molecular Diagnostics, Inivita, Archer, Omicure, Smartcatch and ADMIR companies, as well as the BMS Foundation. P. Laurent-Puig is the President of the *Cancéropole Ile-de-France*, has stock options in the MethysDX company and has received consulting fees from the Biocartis, Amgen, Pierre Fabre and Servier companies, as well as the BMS Foundation.

## Appendix ASupplementary data

Translated abstract
