# Supplementary material for: PFMG2025–integrating genomic medicine into the national healthcare system in France
Source: Lancet Reg Health Eur. 2025 Jan 6;50:101183. doi: 10.1016/j.lanepe.2024.101183 (PMC11910791; doi:10.1016/j.lanepe.2024.101183)
Supplement: Translated Abstract [file mmc1.docx]

*Editors’ disclaimer: This translation in French was submitted by the authors and we reproduce it as supplied. It has not been peer reviewed. Our editorial processes have only been applied to the original abstract in English, which should serve as reference for this manuscript.*

**Translated abstract**

L'intégration de la médecine génomique dans un système national de santé constitue un défi de santé publique qui nécessite de transférer en continu les avancées scientifiques dans le soin et d'assurer une équité d'accès pour tous les patients. La France a été l'un des premiers pays à intégrer le séquençage du génome en pratique clinique à l'échelle nationale, avec l'ambition de poser des diagnostics plus précis et de fournir des traitements personnalisés aux patients. Depuis 2016, le gouvernement français a investi 239 millions d'euros dans le Plan France Médecine Génomique 2025 (PFMG2025), qui s’adresse pour le moment aux patients atteints de maladies rares, de cancers ou prédisposés génétiquement au cancer. Le PFMG2025 a relevé de nombreux défis pour mettre en place une organisation opérationnelle au niveau national. Au 31 décembre 2023, 12.737 résultats ont été rendus aux prescripteurs de patients atteints de maladies rares ou porteurs d’une prédisposition génétique au cancer (délai médian de rendu : 202 jours, rendement diagnostique : 30,6 %) et 3 109 résultats aux prescripteurs de patients atteints de cancers (délai médian de rendu : 45 jours). Les priorités du PFMG2025 sont maintenant d’assurer la viabilité économique, de renforcer les liens avec la recherche, de mieux impliquer les patients et les praticiens et d’encourager les collaborations avec les partenaires européens.
