# Supplementary material for: PFMG2025–integrating genomic medicine into the national healthcare system in France
Source: Lancet Reg Health Eur. 2025 Jan 6;50:101183. doi: 10.1016/j.lanepe.2024.101183 (PMC11910791; doi:10.1016/j.lanepe.2024.101183)
Supplement: Appendix [file mmc2.docx]

**Title: *PFMG2025* - Integrating genomic medicine into the national healthcare system in France**

**Supplementary appendix**

**Contents**

[*PFMG2025* contributors’group 2](#_Toc183599681)

[*PFMG2025* deployment timeline 6](#_Toc183599682)

[The French national framework in the fields of rare diseases, cancer genetic predisposition and cancers 7](#_Toc183599683)

[The *PFMG2025* research pilot projects 8](#_Toc183599684)

[Clinical ‘pre-indications’ selection, setting up of the genomic healthcare pathway 9](#_Toc183599685)

[Ethical, Legal, and Social Issues 11](#_Toc183599686)

[CRefIX (Centre de Référence, d’Innovation, d’Expertise et de Transfert) 12](#_Toc183599687)

[The first two *FMGlabs* (AURAGEN and SeqOIA) 13](#_Toc183599688)

[Detailed common protocols used by both *FMGlabs* 14](#_Toc183599689)

[Clinical pre-indications for genome sequencing in RD/CGP as part of the *PFMG2025* initiative 17](#_Toc183599690)

[Clinical pre-indications for GS/ES/RNAseq in cancers as part of the *PFMG2025* initiative 19](#_Toc183599691)

[Evolution of the number of prescribers and clinical biologists with geographical distribution of prescriptions and clinical biologists over time 20](#_Toc183599692)

[Detailed results provided by both *FMGlabs* 21](#_Toc183599693)

[Four clinical cases of interest in RD diagnosed by genome sequencing 25](#_Toc183599694)

[The 78 causal diagnoses identified after negative exome sequencing among the 2,734 first consecutive prescriptions for RD/CGP 27](#_Toc183599695)

[Tumor topography among the 1,940 complete first consecutive prescriptions for cancers 30](#_Toc183599696)

[Tumor morphological characteristics among the 1,940 complete first consecutive prescriptions for cancers 31](#_Toc183599697)

[List of the 40 genes most frequently mutated with somatic SNVs/Indels of interest returned to MTB for discussing actionability and treatment proposition among the 1,940 complete first consecutive prescriptions for cancers. 32](#_Toc183599698)

[List of the 41 genes most frequently implicated in somatic CNVs of interest returned to the MTB for discussing actionability and treatment proposition among the 1,940 complete first consecutive prescriptions for cancers. 33](#_Toc183599699)

[Secondary use of data for research and data sharing 34](#_Toc183599700)

[References 35](#_Toc183599701)

# *PFMG2025* contributors’group

**Contributors**

Caroline Abadie^1^, Aldja Abderrahmane^2^, Ouarda Abdous^3^, Carine Abel^4^, Oanez Ackermann^5^, Cécile Acquaviva^4,6^, Flavie Ader^5,7^, Salma Adham^8^, Dalila Adjaoud^9^, Alexandra Afenjar^5^, Nathalie Aladjidi^3^, Anne-Sophie Alary^10^, Frédérique Albarel^11^, Sabrina Albert^12^, Lise Allard^3^, Ingrid Allix^13^, Violaine Alunni^14^, Inês F. Amado^15^, Cyril Amouroux^8^, Nicolas André^11^, Chloé Angelini^3^, Mathieu Anheim^14^, Ignacio Antolin Sanfelliz^16^, Thomas Aparicio^5^, Chloé Arfeuille^5,7^, Jean-Benoît Arlet^5^, Lionel Arnaud^5,7^, Pauline Arnaud^5,7^, Guilhem Arnold^17^, Tania Attie-Bitach^5,7^, Marion Aubert-Mucca^18^, Isabelle Audo^19^, Marie-Pierre Audrezet^20^, Maxime Auroux^4^, Céline Auzanneau^12^, Xavier Ayrignac^8^, Ibrahima Ba^5,7^, Anne Bachelot^5^, Delphine Bacq^21^, Séverine Bacrot^22^, Brigitte Bader-Meunier^5^, Sarah Baer^14^, Stéphanie Baert-Desurmont^23^, Laurence Bal-Theoleyre^11^, Ralyath Balogoun^5,7^, Philippe Baltzinger^14^, Guillaume Banneau^18^, Claire Bar^3^, Audrey Barbet^24^, Giulia Barcia^5,7^, Laure Barjhoux^4,6^, Anne Barlier^11^, Vincent Barlogis^11^, Marc Barritault^4,6^, Magalie Barth^13^, Aurore Barthod-Malat^12^, Peggy Baudouin-Cornu^25^, Geneviève Baujat^5^, Amandine Baurand^26^, Jacques-Olivier Bay^2^, Michèle Beau-Faller^14^, Jean-Christophe Beaudoin^27^, Rémi Bellance^16^, Christine Bellanné-Chantelot^5,7^, Carine Bellera^12^, Alexandre Belot^4^, Raihane Ben Abdeljelil^28^, Rihab Ben Sghaier^15^, Joy Benadiba^29^, Stéphanie Benard^30^, Claire Beneteau^3^, Karelle Benistan^5^, Fouzia Benkerdou^5^, Mehdi Benkirane^8^, Jean-François Benoist^5,7^, Patrick R. Benusiglio^5^, Camille Bergès^3^, Anne Bergougnoux^8^, Maureen Bernadach^31^, Emilien Bernard^4^, Valérie Bernard^3^, Virginie Bernard^9,6^, Dounia Beroug^28^, Aurélie Berrard^23^, Jérôme Bertherat^5^, Pascaline Berthet^32^, Clotilde Berthier^33^, Aurélia Bertholet-Thomas^4^, Jean-Philippe Bertocchio^5^, François Bertucci^10^, Céline Besse^21^, Elsa Besse-Pinot^2^, Didier Bessis^8^, Pauline Beuvain^30^, Stéphane Bezieau^33^, Marie Bidart^6,9^, Ivan Bièche,^34^ Margaux Biehler^14^, Thierry Bienvenu^5,7^, Frédéric Bilan^35^, Clarisse Billon^5,7^, Christine Binquet^36^, Elise Bismuth^5^, Varoona Bizaoui^37^, Pierre Blanc^5,7^, Hélène Blanché^27^, Jean-Yves Blay^6,38^, Adrien Bloch^5^, Gilles Bloch^15^, Agnès Bloch-Zupan^14^, Béatrice Bocquet^8^, Morgane Boedec^14^, Catherine Boileau^5^, Maureen Boissinot^39^, Anne Boland^21^, Pierre-Adrien Bolze^4^, Valérie Bonadona^38^, Julia Bonastre^40,41^, Nathalie Bonello-Palot^11^, Adeline-Alice Bonnard^5,7^, Raphaël Borie^5^, Damien Botsen^42^, Mohamed Bouattour^5^, Marion Bouctot^35^, Natacha Bouhours-Nouet^13^, Jérôme Bouligand^5,7^, Ahmed Bouras^6,37^, Thomas Bourgeron^43^, Jean-Louis Bourges^5^, Emmanuelle Bourrat^5^, Guilaine Boursier^8^, Guilhem Bousquet^5^, Philippe-Jean Bousquet^44^, Simon Boussion^23^, Lucile Boutaud^5,7^, Julian Boutin^3^, Patrice Bouvagnet^16^, Claire Bouvattier^5^, Sandrine Boyault^6,38^, Aude Brac de la Perriere^4^, Mehdi Brahmi^38^, Valentine Brard^45^, Mathilde Brasseur^46^, Nadège Brazzalotto^3^, Dominique Brémond-Gignac^5^, Audrey Briand-Suleau^5,7^, Claire Briet^13^, Pierre-Paul Bringuier^4,6^, Céline Bris^13^, Elise Brischoux-Boucher^47^, Karine Brochard^18^, Martin Broly^8^, Laura Brosseau^8^, Ange-Line Bruel^25^, Perrine Brunelle^23^, Virginie Bubien^12^, Bruno Buecher^7,34^, Alexandre Buffet^5,7^, Adrien Buisson^6,38^, Lydie Burglen^5,7^, Cyril Burin Des Roziers^5,7^, Nelly Burnichon^5,7^, Tiffany Busa^11^, Mathilde Cabart^12^, Sara Cabet^4,6^, Charlotte Caille-Benigni^48^, Claire Caillot^4^, Christophe Calvin^25^, Anne Cambon-Thomsen^49,50^, Claude Cances^18^, Alexandre Cantan^3^, Liana Carausu^20^, Aurélia Carbasse^8^, Cédric Carbonneil^51^, Bertrand Cariou^33^, Olivier Caron^40^, Sylvain Carras^9^, Stéphanie Cartalat^4^, Kévin Cassinari^22^, Martin Castelle^5^, Laurent Castéra^32^, Frédéric Castinetti^11^, Julie Catteau^52^, Roseline Caumes^24^, Aurélien Caux^53^, Mathias Cavaillé^6,31^, Hélène Cavé^5,7^, Aurélie Caye-Eude^5,7^, Cécile Cazeneuve^4,6^, Tristan Celse^30^, Noémie Celton^54^, Camille Cenni^55^, Jasmin Cévost^56^, Rania Chaabna^57^, Brigitte Chabrol^11^, Ilyas Challet^5^, Clélia Chalumeau^1^, Pascal Chambon^22^, Albain Chansavang^5,7^, Jean-Baptiste Chanson^14^, Sébastien Chapelant^4,6^, Fabienne Charbit-Henrion^5,7^, Perrine Charles^5^, Sybil Charrière^4^, Philippe Charron^5^, Nicolas Chassaing^18^, Nicolas Chatron^4,6^, Boris Chaumette^5^, Catherine Chaussain^5^, Annabelle Chaussenot^29^, David Cheillan^4,6^, Olivier Chenavier^4,6^, Bertrand Chesneau^18^, Louise-Marie Chevalier^58^, Christine Chomienne^15^, Cécile Chougnet^5^, Sophie Christin-Maitre^5^, Marine Chuet^47^, Emmanuelle Clappier^5,7^, Johanna Clet^3^, Mélanie Cloteau^45^, Thomas Cluzeau^29^, Guillaume Cogan^5,7^, Benjamin Cogné^33^, Alicia Cohen^14^, Camille Cohen^59^, Odile Cohen-Haguenauer^5^, Martine Cohen-Solal^5^, Chrystelle Colas^48^, Estelle Colin^13^, Corinne Collet^5,7^, Delphine Collin-Chavagnac^4,6^, Éloïse Colliou^18^, Marie-Agnès Collonge-Rame^47^, Maxime Colmard^8^, Stéphanie Coopman^24^, Lucie Coppin^24^, Elodie Coquan^32^, Valérie Cormier-Daire^5^, Nadège Corradini^38,60^, Carole Corsini^8^, Mireille Cossée^8^, Thibault Coste^5,7^, Sophie Cotteret^61^, Rachel Cottet^62^, Christine Coubes^8^, Florence Coulet^5,7^, Nathalie Couque^5,7^, Philippe Couratier^52^, Marie Courbebaisse^5^, Olivier Courbette^29^, Cécile Courdier^3^, Juliette Coursimault^23^, Thomas Courtin^5^, Lucien Courtois^5,7^, Fabienne Coury^4^, Laure Coutos-Thévenot^12^, Charles Coutton^9,6^, Isabelle Creveaux^2,6^, Etienne Crickx^5^, Louise Crivelli^61^, Marc Cuggia^63^, Laurence Cuisset^5,7^, Hubert Curcio^32^, Aurore Curie^4^, Veronica Cusin^5^, Noémie Da Costa^57^, Lionel Da Cruz^64^, Éric Dahlen^47^, Antoine Dardenne^5^, Benjamin Dauriat^52^, Nell Dausse^46^, Alix De Becdelièvre^5,7^, Florence De Fraipont^6,9^, Élisa De La Cruz^8^, Thibault De la Motte Rouge^61^, Sandrine De Montgolfier^15,65^, Antoine De Pauw^34^, Aurélien De Reyniès^5,7^, Jean-Madeleine De Sainte Agathe^5,7^, Marie De Tayrac^63^, Anne-Sophie Defachelles^66^, Michaël Degaud^7,40^, Caroline Deiller^8^, Éric Delabesse^18^, Leslie Delachaux^5,7^, Andrée Delahaye-Duriez^5,7^, Jean-François Deleuze^21^, Hélène Delhomelle^34^, Christelle Delmas^15^, Capucine Delnatte^1^, Catherine Delorme^24^, Richard Delorme^5^, Bénédicte Demeer^53^, Caroline Demilly^67^, Philippe Denizeau^63^, Isabelle Denjoy^5^, Anne-Sophie Denommé-Pichon^26^, Christel Depienne^5,7^, Nicolas Derive^5,7^, Flora Dervillé^32^, Vincent Des Portes^4^, Isabelle Desguerre^5^, Béatrice Desnous^11^, Camille Desseignes^5^, Françoise Devillard^9^, Manjula Deville^5^, Nelly Dewulf-Pasz^18^, Claire-Marie Dhaenens^24^, Klaus Dietrich^9^, Anne Dieux^24^, Mody Diop^68^, Emmanuel Disse^4^, Samir Djaber^5^, Christine Do Cao^24^, Hélène Dollfus^14^, Louis Domenach^3^, Jean Donadieu^5^, Bruno Donadille^5^, Aurore Dougé^2^, Hélène Dreyfus^69^, Séverine Drunat^5,7^, Danièle Dubois-Laforgue^5^, Christèle Dubourg^63^, Charlotte Dubucs^18^, Jean-Christophe Dubus^11^, Matthieu Duchmann^5,7^, François Ducray^4^, Marion Ducrotverdun^5,7^, Florence Duffaud^11^, Yannis Duffourd^26^, William Dufour^4^, Gwénaëlle Duhil de Bénazé^29^, Yves Dulac^18^, Olivier Dunand^30^, Denis Dunoyer de Segonzac^4,6^, Célia Dupain^34^, Nicolas Duployez^24^, Anaïs Dupré^12^, Aurélien Dupré^38^, Sophie Dupuis-Girod^4^, Romain Duquet^5^, Alice Durand^4^, Benjamin Durand^14^, Isabelle Durand-Zaleski^5^, Xavier Durando^31^, Alexandra Durr^5^, Lauriane Eberst^70^, Patrick Edery^4^, Matthieu Egloff^35^, Salima El Chehadeh^14^, Laïla El Khattabi^5,7^, Camille Engel^47^, Mathilde Entresangle^5^, Hélène Espérou^15^, Florence Esselin^8^, Pascaline Etancelin^22^, Clémence Evrevin^5^, Claire Ewenczyk^5^, Alain Eychene^15^, Thomas Eychenne^56^, Andra Ezaru^29^, Vincent Fabry^18^, Laurence Faivre^26^, Marie Faoucher^63^, Clémentine Faure^4,6^, Julien Fauré^6,9^, Anne-Laure Fauret-Amsellem^5,7^, Eva Feigerlova^45^, François Feillet^45^, Laurène Fenwarth^24^, Claude Férec^20^, Patricia Fergelot^3^, Anthony Ferrari^6,37^, Carole Ferraro-Peyret^4,6^, Jean-Paul Feugeas^47^, Claire Fieschi^5^, Alice Fievet^7,39^, Marc Fila^8^, Rémi Fillatre^2^, Mathilde Filser^5,7^, Bertrand Fin^21^, Mathieu Fiore^3^, Nelly Firmin^71^, Pascale Flandrin-Gresta^6,72^, Aude Flechon^38^, Benjamin Fournier^5^, Cécile Fragny^64^, Marie-Céline Francois-Heude^8^, Bruno Francou^29^, Thierry Frébourg†^23^, Véronique Fressart^5,7^, Mathilde Frétigny^4,6^, Benoit Funalot^5,7^, Mathieu Fusaro^18^, Pauline Gaignard^5,7^, Estelle Gandjbakhch^5^, Benjamin Ganne^8^, Aurore Garde^26^, Vincent Gatinois^8^, Céline Gaucher^5,7^, Léa Gaudillat^26^, Philippe Gaulard^5,7^, Lucas Gauthier^4,6^, Mathilde Gay-Bellile^31^, Damien Geneste^12^, David Geneviève^8^, Emmanuelle Genin^73^, Sandrine Genoux^4,6^, Birgit Geoerger^40^, Véronique Geoffroy^73^, Mathieu Georget^5,7^, Bénédicte Gérard,^14^ Witold Gertych^4^, Souad Gherbi Halem^5^, Karima Ghorab^52^, Romane Gille^37^, Charlène Gillet^13^, Marion Gillibert-Yvert^54^, Olivier Gilly^55^, Anne-Paule Gimenez-Roqueplo^5,7^, Sophie Giraud^12^, Barbara Girerd^5^, François Girodon^26^, Olga Glazunova^11^, Delphine Gobert^5^, Cyril Goizet^3^, Zeynep Gokce-Samar^4^, Lisa Golmard^7,34^, Carlos Gomez-Roca^74^, Emmanuel Gonzales^5^, Magali Gorce^18^, Marie-Clémence Gorenstein^5^, Kévin Gorrichon^56^, Frédéric Gottrand^24^, Laetitia Gouas^2,6^, Stéphanie Gourdon^30^, Pierre Gourdy^18^, Aurélie Gouronc^14^, Claire Goursaud^4,6^, Gaëlle Gousse^72^, Evan Gouy^4^, Odile Goze-Martineau^24^, Diane Gozlan^15^, David Grabli^5^, Margaux Gras^56^, Maude Grelet^75^, Laetitia Gressin^27^, Nathalie Grivel^15^, Sarah Grotto^5^, Virginie Grouthier^3^, Solange Grunenwald^18^, Olivier Grunewald^24^, Paul Gueguen^54^, Cécile Guérin^5^, Anne-Marie Guerrot,^23^ Stéphanie Guey^5^, Nathalie Guffon^4^, Agnès Guichet^13^, Romain Guièze^2^, Marine Guillaud-Bataille^5,7^, Francis Guillemin^76^, Erell Guillerm^5,7^, Yann Guillermin^38^, Virginie Guillet-Pichon^13^, Isabelle Guillou^34^, Rosine Guimbaud^18^, Anne Guimier^5^, Claire Guissart^55^, Eric Guittet^77^, Nathalie Guy^2^, Alice Hadchouel^5^, Hamza Hadj Abdallah^5,7^, Smail Hadj-Rabia^5^, Samy Hadjadj^33^, Mehdi Hage-Sleiman^5,7^, Corinne Haioun^5^, Sara Halawi^29^, Abderaouf Hamza^7,34^, Perrine Hanau^46^, Nadine Hanna^5,7^, Radu Harbuz^6,9^, Gaëlle Hardy^6,9^, Carine Hauspie^24^, Sandrine Hayette^4^, Jean-Michel Heard^77^, Maël Heiblig^4^, Solveig Heide^5^, Laurence Heidet^5,7^, Marcia Henry^11^, Véronique Hentgen^21^, Bénédicte Héron^5^, Delphine Héron^5^, Dominique Hervé^5^, Anthony Herzig^73^, Pierre Hirsch^5,7^, Antoine Hommais^78^, Jérôme Honnorat^4^, Edgar Horta^47^, Claude Houdayer^22^, Pascal Houillier^5^, Sarah Huet^4,6^, Jean-Pierre Hugot^5^, Yoann Huguenin^3^, Marc Humbert^5^, Marie-Laure Humbert-Asensio^35^, Laure Huot^4^, Norbert Ifrah^78^, Frédéric Illouz^13^, Apolline Imbard^5,7^, Marion Imbert-Bouteille^71^, Nicolas Isambert^35^, Bertrand Isidor^33^, Antoine Italiano^12^, Raphaël Itzykson^5^, Sylvie Jaillard^63^, Yvan Jamilloux^4,6^, Alexandre Janin^4,6^, Louis Januel^4,6^, Cécile Javelot-Jacquelin^14^, Médéric Jeanne^54^, Guillaume Jedraszak^53^, Isabelle Jéru^5,7^, Xavier Jeunemaitre^5^, Éric Jeziorski^8^, Florence Jobic^53^, Philippe Joly^4,6^, Laurence Jonard^5,7^, Guillaume Jondeau^5^, Natalie Jones^12^, Jean-Marie Jouannic^5^, Anne Jouinot^5^, Pierre-Simon Jouk^9^, Yohann Jourdy^4,6^, Kévin Jousselin^5,7^, Anne Jouvenceau^15^, Charlotte Jubert^3^, Sophie Julia^18^, Anne-Laure Jurquet^11^, Aurélien Juven^2^, Maud Kamal^7,34^, Pascal Kantapareddy^4^, Elsa Kaphan^11^, Lucie Karayan-Tapon^35^, Edwige Kasper^22^, Lara Kerbellec^54^, Boris Keren^5,7^, Emmanuel Khalifa^56^, Philippe Khau Van Kien^55^, Sihem Kheddouci^6^, Caroline Kientz^72^, Rathana Kim^5,7^, Antjie Knapke^9^, Michel Koenig^8^, Isabelle Kone^5^, Marina Konyukh^5,7^, Raphaël Kormann^45^, Manoëlle Kossorotoff^5^, Paul Kuentz^47^, Florence Kyndt^33^, Anaïs L'Haridon^5,7^, Philippe Labrune^5^, Marilyn Lackmy^79^, Didier Lacombe^3^, Ludovic Lacroix^7,39^, Fanny Laffargue^2^, Ghizlene Lahlou^5^, Yec'han Laizet^12^, Laetitia Lambert^45^, Jérôme Lamoril^5,7^, Audrey Lamouroux^8^, Émilie Landais^46^, Samuel Landman^5,7^, Elise Landry^15^, Hélène Lapillonne^5^, Anne-Sophie Lapointe^64^, Lise Larcher^5,7^, Pierre Lardeux^4^, Laetitia Largeaud^18^, Etienne Larger^5^, Louis Larrouquere^38^, Hélène Lasolle^4^, Eulalie Lasseaux^12^, Xenia Latypova^5,7^, Tiphany Laurens^30^, Camille Laurent^18^, Pierre Laurent-Puig^5,7^, Géraldine Lautrette^52^, Thomas Lauvray^52^, Benoît Lavallart^77^, Cécile Lavenu-Bombled^5^, Noémie Laverdure^4^, Yannick Le Bris^33^, Catherine Le Chalony^77^, Nathalie Le Du^54^, Gaëlle Le Folgoc^73^, Gerald Le Gac^20^, Jessica Le Gall^7,34^, Edouard Le Guillou^5,7^, Xavier Le Guillou^35^, Gwenaël Le Guyader^35^, Maryannick Le Ray^11^, Olivia Le Saux^38^, Christophe Le Tourneau^34^, Benjamin Lebecque^2,6^, Loïc Lebellec^66^, Élise Lebigot^5,7^, Pierre Leblond^38,60^, Nicolas Leboulanger^5^, Laure Lebras^38^, Anne-Sophie Lebre^46^, Louis Lebreton^3^, François Lecoquierre^23^, Mathilde Lefebvre^80^, Marine Legendre^3^, Camille Leglise^53^, Clémentine Legrand^9^, Daphné Lehalle^5^, Catherine Lejeune^35^, Christine Lemaitre^15^, Raphaël Leman^32^, Mathis Lepage^31^, Alban Lermine^5,7^, Karen Leroy^5,7^, Gaëtan Lesca^4,6^, Marion Lesieur-Sebellin^5,7^, Mélanie Letexier^56^, Franck Lethimonnier^15^, Lucie Levaillant^13^, Jonathan Levy^5,7^, Yves Levy^15^, Pascale Lévy^81^, Ludovic Lhermitte^5,7^, Agnès Linglart^5^, Clément Lionnet^6,9^, Doriane Livon^10^, Laurence Lode^46^, Magalie Lodin^13^, Jonathan Lopez^4,6^, Maureen Lopez^5,7^, Alain Lortholary^82^, Malek Louha^5,7^, Camille Louvrier^5,7^, Thomas E. Ludwig^73^, Auriane Luvet^4,6^, Stanislas Lyonnet^5^, Caroline Makowski^9^, Valérie Malan^5,7^, Martial Mallaret^9^, Delphine Mallet^4,6^, Stéphanie Mallet^11^, Marion Malphettes^5^, Nathalie Manaud^25^, Pierre Mancini^12^, Sylvain Manfredi^26^, Sylvie Manouvrier^24^, Luke Mansard^8^, Sandrine Mansard^2^, Lamisse Mansour-Hendili^5,7^, Ludovic Mansuy^45^, Julien Maquet^18^, Ambroise Marçais^5^, Alice Marceau-Renaut^24^, Perrine Marec-Berard^38,60^, Cécilia Marelli^8^, Gaëlle Marenne^73^, Isabelle Marey^9^, Jennifer Margier^4^, Henri Margot^3^, Guillaume Marie^83^, Victor Marin^3^, Laetitia Marisa^5,7^, Sandrine Marlin^5^, Émeline Marquant^11^, Valentine Marquet^52^, Luisa Marsili^24^, Amaury Martin^34^, Laetitia Martinerie^5^, Anna Maruani^5^, Pauline Marzin^5^, Christophe Massard^40^, Emmanuelle Masson^20^, Flavie Mathieu^15^, Marion Mathieu^84,85^, Simone Mathoulin-Pelissier^57^, Flore Matthieu^4^, Mathilde Mauras^72^, Aurélien Maureille^38^, Benoit Mazel^26^, Mary Mazeres^86^, Anne Mc Leer^6,38^, Isabelle Melki^5^, Rita Menassa^4,6^, Aurélie Méneret^5^, Julie Menjard^20^, Anne Mercier^35^, Elodie Merieau^54^, Marie-Sophie Merlin^45^, Jean-Philippe Merlio^3^, Cécile Meslier^44^, Laurent Mesnard^5^, Sandrine Mestre-Godin^8^, Corinne Metay^5,7^, Sandrine Meunier^4^, Pierre Meyer^8^, Vincent Michaud^3^, Laurence Michel-Calemard^4,6^, Cyril Mignot^5^, Marguerite Miguet^87^, Gilles Millat^4,6^, Tristan Mirault^5^, Albane Miron de l'Espinay^64^, Clémence Molac^5^, Arnaud Molin^88^, Julie Mondet^6,9^, Faustine Monin^4^, Pauline Monin^4^, Audrey Monneur^10^, Sophie Monnot^5,7^, David Montani^5,7^, Elodie Morel^4^, Godelieve Morel^30^, Valérie Morel^27^, Jessica Moretta^10^, Fanny Morice-Picard^3^, Lucie Morillon^15^, Carole Morin^4^, Marie-Emmanuelle Morin-Meschin^58^, Philippe Morlat^64^, Despina Moshous^5^, Emmanuelle Mouret-Fourme^34^, Alice Moussy^56^, Sébastien Moutton^3^, Kévin Mouzat^55^, Romane Muletier^2^, Jean Muller^14^, Marie Muller^45^, Aleksandra Nadaj-Pakleza^14^, Sophie Nambot^26^, Nadia Nathan^5^, Caroline Nava^5,7^, Juliette Nectoux^5,7^, Jeanne Netter^5^, Florent Neumann^26^, Julien Neveu^29^, Zoé Nevière^32^, Laetitia Nguyen^5^, Tanguy Niclass^35^, Gaël Nicolas^23^, Laury Nicolas^2,6^, Massih Ningarhari^24^, Catherine Nogues^10^, Cécile Novello^4^, Frédérique Nowak^15^, Sylvie Odent^63^, Marie-Françoise Odou^24^, Robert Olaso^21^, Sarah Otmani^5,89^, Caroline Ovaert^11^, Laurence Pacot^5,7^, Mélanie Pages^7,34^, Catherine Paillard^14^, Aurélien Palmyre^5^, Eleni Panagiotakaki^4^, Myriam Pannard^90,91^, Anne Paoletti^77^, Maria T. Papadopoulou^4^, Matthildi Papathanasiou^4^, Véronique Paquis^77^, Béatrice Parfait^5,7^, Camille Paris^24^, Clara Paris^4^, Françoise Paris^8^, Éric Pasmant^5,7^, Marlène Pasquet^18^, Marie Passet^5,7^, Cédric Pastoret^63^, Olivier Patat^18^, Léa Patay^26^, Antoine Paul^5^, Céline Pebrel-Richard^2,6^, Cristina Peduto^5^, Regis Peffault de Latour^5^, Antoine Pegat^4^, Annick Pelletier^11^, Valérie Pelletier^14^, Fanny Pellisson^4,6^, Perrine Pennamen^3^, Victor Pereira^47^, Julie Pernin-Grandjean^4^, Julien Péron^4^, Alexandre Perrier^5,7^, Lionel Perrier^38,92^, Laurence Perrin^5^, Isabelle Perthus^2^, Arnaud Petit^5^, Audrey Petit^11^, Florence Petit^24^, François Petit^28^, Yuliya Petrov^55^, Hugo Peyre^8^, Christophe Philippe^93^, Juliette Piard^47^, Élise Pierre-Noël^7,34^, Gaëlle Pierron^7,34^, Clément Pimouguet^94^, Véronique Pingault^5,7^, Stéphane Pinson^4,6^, Emmanuelle Pion^8^, Julie Plaisancié^18^, Marc Planes^20^, Pauline Planté-Bordeneuve^24^, William Plas^5^, Morgane Plutino^29^, Ludivine Poignie^12^, Vianney Poinsignon^5,7^, Marilyne Poirée^29^, Nicolas Pons^4,6^, Bénédicte Pontier^2^, Valérie Porquet-Bordes^18^, Camille Porteret^35^, Delphine Potier^10^, Louis Potier^5^, Damien Pouessel^74^, Laura Poujade^8^, Marie Preau^90,91^, Claude Preudhomme^24^, Fabienne Prieur^72^, Vincent Probst^33^, Vincent Procaccio^13^, Caroline Prot-Bertoye^5^, Delphine Prunier^13^, Jacques Puechberty^8^, Mathilde Pujalte^4,6^, Leila Qebibo^5,7^, Sylvia Quemener-Redon^20^, Isabelle Quérée^8^, Susana Quijano-Roy^5^, Nicolas Quirin^95^, Caroline Racine^26^, Sandra Raimbault^66^, Judith Raimbourg^1^, Marine Rajaoba^5^, Thomas Rambaud^5,7^, Carole Ramirez^72^, Francis Ramond^6,72^, Kara Ranguin^79^, Antonio Rausell^5,7^, Jean-Marie Ravel^45^, Claudia Ravelli^5^, Gerald Raverot^4^, Isabelle Ray-Coquard^38^, Caroline Raynal^8^, Patricia Réant^3^, Vinciane Rebours^5^, Richard Redon^33^, Yves Réguerre^30^, Philippe Reix^4^, Cécile Renard^4,60^, Mathilde Renaud^45^, John Rendu^6,9^, Céline Renoux^4,6^, Romain Rey^67^, Rachel Reynaud^11^, Lucie Rhinan^45^, Florence Riant^5,7^, Florence Riccardi^75^, Pascale Richard^5,7^, Agathe Ricou^32^, Vincent Rigalleau^3^, Marlène Rio^5^, Axelle Rivière^45^, Patrick Robelin^4^, Marion Robert^30^, Thomas Robert^96^, Barbara Rohmer^4^, Pauline Romanet^11^, Arnaud Romoli^54^, Sophie Rondeau^5,7^, Caroline Rooryck^3^, Bertrand Roquelaure^11^, Jérémie Rosain^5,7^, Massimiliano Rossi^4^, Sylvie Rossignol^14^, Anya Rothenbuhler^5^, Nadège Rouel^2^, Marine Rouillon^56^, Anne-Françoise Roux^8^, Nathalie Roux-Buisson^9^, Cécile Rouzier^29^, Emmanuel Roze^5^, Lyse Ruaud^5^, Valentin Ruault^8^, Claire Ruysschaert^12^, Esma Saada^28^, Samira Saadi - Ait El Mkadem^29^, Thoueiba Saandi^4^, Niki Sabour^35^, Sabrina Sacconi^29^, Raphaël Saffroy^5,7^, Hana Safraou^26^, Virginie Saillour^5,7^, Aude Saint Pierre^73^, Cécile Saint-Martin^5,7^, Pierre Saintigny^6,38^, Gaëlle Salaun^2,6^, David Salgado^44^, Laurence Salle^52^, Didier Samuel^15^, Damien Sanlaville^4,6^, Laure Sapey-Triomphe^4,6^, Sabine Sarnacki^5^, Elisabeth Sarrazin^16^, Catherine Sarret^2^, Véronique Satre^6,9^, Pascale Saugier-Veber^23^, Paul Saultier^11^, Laure Saumet^8^, Élise Schaefer^14^, Nicolas Scheyer^45^, Isabelle Schiff^9^, Gudrun Schleiermacher^34^, Nicolas Schleinitz^11^, Caroline Schluth-Bolard^14^, Anouck Schneider^8^, Bertrand Schwartz^77^, Jean-Marc Sebaoun^27^, Margaux Serey-Gaut^5^, Hassan Serrier^4^, Aude Servais^5^, Marine Serveaux-Dancer^6,9^, Nicolas Sevenet^12^, Antoine Seyve^4^, Alicia Sibony-Cohen^14^, Flore Sicre De Fontbrune^5^, Sabine Sigaudy^11^, François Sigaux^78^, Fatoumata Simaga^34^, Victor Simmet^34^, Pauline Simon^47^, Sophie Simon^4^, François Sirveaux^45^, Thomas Smol^24^, Guilhem Solé^3^, Gwendoline Soler^2,6^, Pauline Solignac^97^, Marie-Hélène Soriani^29^, Isabelle Soubeyran^12^, Jean Soulier^5,7^, Laurent Spelle^5^, Brian Sperelakis-Beedham^5,7^, Marco Spinazzi^13^, Marta Spodenkiewicz^30^, Anne Spraul^5,7^, Barbara Squiban^98^, Arunya Srikaran^5^, Julie Steffann^5,7^, Anamaria Stetco^35^, Radka Stoeva^99^, Tanya Stojkovic^5^, Dominique Stoppa-Lyonnet^34^, Felipe Suarez^5^, Pierre Sujobert^4,6^, Juliette Svahn^4^, Minh-Chau Ta^5,7^, Anne-Claude Tabet^5,7^, Gaëlle Tachon^6,38^, Matthias Tallegas^54^, Anne Tallet^54^, Pierre-Edmond Tambourin^15^, Julie Tandonnet^3^, Véronique Tardy^4,6^, Emmanuelle Tavernier^72^, Dimitri Tchernitchko^5,7^, Marie-Hélène Teillon-Berranger^4,6^, Julie Tenenbaum^8^, Jordan Teoli^4,6^, Marine Tessarech^24^, Benoit Tessoulin^33^, Mylène Tharreau^8^, Christel Thauvin-Robinet^15,26^, Nathalie Theou-Anton^5,7^, Julien Thevenon^6,9^, Anne Thomas^4,6^, Laure Thomas^4^, Quentin Thomas^26^, Cécile Thomas-Teinturier^5^, Nathalie Tieulie^29^, Julie Tinat^3^, Camille Tlemsani^5,7^, Sylvie Tondeur^6,9^, Lucie Tosca^5,7^, Diego Tosi^71^, David Tougeron^35^, Philippe Touraine^5^, Elisabeth Tournier-Lasserve^5,7^, Annick Toutain^54^, Frédéric Tran Mau-Them^26^, Christine Tranchant^14^, Olivier Trédan^38^, Aurélien Trimouille^3^, Jean-Noël Trochu^33^, Vincent Tronel^100^, Cécile Trouba^4^, Marie-Élise Truchetet^3^, Nathalène Truffaux^12^, Amel Tsalamlal^38^, Edouard Turlotte^56^, Violette Turon^56^, Maud Tusseau^4,6^, Nancy Uhrhammer^6,31^, Christel Vaché^8^, Stéphanie Valence^5^, Thibaud Valentin^74^, René Valero^11^, Sophie Valleix^5,7^, Marion Vallet^18^, Hélène Vanacker^38^, Pierre Vande-Perre^74^, Yves Vandenbrouck^25^, Clémence Vanlerberghe^24^, Rosa Vargas-Poussou^5,7^, Camille Vatier^5^, Vincent Vauchel^64^, Dominique Vaur^32^, Lourdes Velo-Suarez^73^, Laurence Venat^52^, Gabriella Vera^23^, Camille Verebi^5,7^, Célia Verley^29^, Loïc Verlingue^37^, Christophe Verny^13^, Lauren Veronese^2,6^, Nelly Verotte^40^, Benjamin Verret^40^, Yoann Vial^5,7^, François Vialard^7,59^, Alain Viari^6,56^, Marie Vidailhet^5^, Dominique Vidaud^5,7^, Michel Vidaud^5,7^, Stéphane Vignes^101^, Clothilde Vigouroux^8^, Laurent Villard^11^, Laurent Villeneuve^4^, Patricia Villié^30^, Marie-Charlotte Villy^34^, Lara Vinauger^5,7^, Armelle Vinceneux^38^, Anne Vincenot^5,7^, Christine Vinciguerra^4,6^, Antonio Vitobello^26^, Patrick Vourc'h^54^, Aurore Vozy^5^, Marie-Laure Vuillaume-Winter^54^, Sandrine Vuillaumier-Barrot^5,7^, Karim Wahbi^5^, Cédrick Wallet^12^, Thomas Walter^4^, Ulrike Walther-Louvier^8^, Sarah Watson^34^, Anne-Christine Waymel^4,6^, Sara Weinhard^46^, Camille Wicker^14^, Marjolaine Willems^8^, Justine Wourms^45^, Antoine Wyrebski^9^, Kévin Yauy^8^, Mohamad Zaidan^5^, Ariane Zaloszyc^14^, Hélène Zattara^11^, Christophe Zawadzki^24^, Alban Ziegler^45^

**Affiliations**

^1^Institut de Cancérologie de l’Ouest, Saint-Herblain, ^2^CHU Clermont-Ferrand, ^3^CHU Bordeaux, ^4^HCL Lyon, ^5^APHP Paris, ^6^FMGlab AURAGEN, ^7^FMGlab SeqOIA, ^8^CHU Montpellier, ^9^CHU Grenoble, ^10^Institut Paoli-Calmettes, Marseille, ^11^APHM Marseille, ^12^Institut Bergonié, Bordeaux, ^13^CHU Angers, ^14^Hôpitaux Universitaires de Strasbourg, ^15^Institut national de la santé et de la recherche médicale (Inserm), ^16^CHU Martinique, ^17^CH Valence, ^18^CHU Toulouse, ^19^CHNO des Quinze-Vingts, Paris, ^20^CHU Brest, ^21^Centre National de Recherche en Génomique Humaine (CNRGH), CEA, ^22^CH Versailles, ^23^CHU Rouen, ^24^CHU Lille, ^25^Commissariat à l'Energie Atomique (CEA), ^26^CHU Dijon-Bourgogne, ^27^Fondation Jean Dausset-CEPH Paris, ^28^Centre Antoine Lacassagne, Nice, ^29^CHU Nice, ^30^CHU Réunion, ^31^Centre Jean Perrin, Clermont-Ferrand, ^32^Centre François-Baclesse, Caen, ^33^CHU Nantes, ^34^Institut Curie, Paris, ^35^CHU Poitiers, ^36^CIC-EC, Inserm, CHU Dijon, ^37^CH de l’estran Pontorson, ^38^Centre Léon Bérard, Lyon, ^39^CIC, Inserm, CHU Rennes, ^40^Gustave Roussy, Villejuif, ^41^Inserm 1018, ^42^Institut Godinot, Reims, ^43^Institut Pasteur, Paris, ^44^Collecteur Analyseur de Données (CAD), ^45^CHRU Nancy, ^46^CHU Reims, ^47^CHU Besançon, ^48^CH Troyes, ^49^Inserm 1295 , ^50^University Toulouse III Paul Sabatier, ^51^Haute Autorité de Santé, ^52^CHU Limoges, ^53^CHU Amiens, ^54^CHU Tours, ^55^CHU Nîmes, ^56^Centre de référence, d'innovation, d'expertise et de transfert (CRefIX), ^57^CIC-EC, Inserm, Institut Bergonié, Bordeaux, ^58^Institut de Cancérologie de l’Ouest, Angers, ^59^CHI Poissy, ^60^Institut d’Hématologie et d’Oncologie Pédiatrique, Lyon, ^61^Centre Eugène Marquis, Rennes, ^62^CH Savoie Chambéry, ^63^CHU Rennes, ^64^Health Ministry, ^65^Aix-Marseille Université, ^66^Centre Oscar Lambret, Lille, ^67^CH Le Vinatier, Bron, ^68^CH Cayenne, ^69^Sainte-Catherine - Institut du Cancer, Avignon, ^70^Institut de Cancérologie de Strasbourg, ^71^Institut du Cancer de Montpellier, ^72^CHU Saint-Etienne, ^73^Inserm 1078, ^74^Institut Universitaire du Cancer de Toulouse - Oncopole, ^75^CH Toulon, ^76^CIC-EC, Inserm, CHRU Nancy, ^77^Higher Education and Research Ministry, ^78^Institut National du Cancer (INCa), ^79^CHU Guadeloupe, ^80^CHU Orléans, ^81^Agence de la Biomédecine, ^82^Hôpital privé du confluent, Nantes, ^83^CH Boulogne, ^84^Espace de réflexion éthique PACA-Corse, ^85^Tous Chercheurs, ^86^Hôpital Américain de Paris, ^87^CHR Mulhouse, ^88^CHU Caen, ^89^Banque Nationale de Données Maladies Rares (BNDMR), ^90^Inserm 1296, ^91^Université Lumière, Lyon, ^92^GATE UMR 5824, ^93^CHR Metz-Thionville, ^94^Alliance Maladies Rares, ^95^CHT Gaston-Bourret, Nouméa, ^96^Hôpital Saint Joseph Marseille, ^97^Inserm Transfert, ^98^Inserm 1312, ^99^CH Le Mans, ^100^Amis FSH, ^101^Hôpital Cognacq-Jay, Paris

# *PFMG2025* deployment timeline

Various actions were progressively taken to develop infrastructures, standardize practices, inform patients, organize genomic healthcare pathways, international collaborations, training and the medico-economic study since the launch of PFMG2025 in 2016 leading to a total of 26,610 prescriptions (22,259 validated in MDM for RD/CGP and 4,351 validated in MTB for cancers).

Figure showing the *PFMG2025* deployment timeline (CAD: national facility for data storage and intensive calculation; CRefIX : reference center for innovation, assessment, and transfer; CSE: scientifical and ethical committee; ctDNA: circulating tumor DNA; ELSI: Ethical, Legal, and Social Issues; ERDERA: European Rare Diseases Research Alliance; ES: exome sequencing; FFPE: Formalin-fixation and paraffin-embedding; GDI: Genomic Data Infrastructure; GS: genome sequencing; GoE: Genome of Europe; MDM: multidisciplinary meeting; MoU: Memorandum of Understanding; MTB: multidisciplinary tumor board; 1+MG: 1 Million Genomes; RD/CGP: rare diseases and cancer genetic predisposition).

# The French national framework in the fields of rare diseases, cancer genetic predisposition and cancers

The *PFMG2025* initiative relies on a solid French national framework, structured for many years in the fields of rare diseases (RD), cancer genetic predisposition (CGP) and cancer. Indeed, France was the first country in Europe to implement successive national plans for RD (PNMR1-3 since 2004), jointly led by the Ministries of Health and of Research, in close collaboration with patient support organizations whose role has been decisive in the development of this public health policy in RD. After the setting up of centers of reference for RD (CRMR, “Centre de Référence Maladies Rares”) with primary mission of coordinating care, training and research in a defined area of RD, 23 thematic RD health networks (FSMR, “Filières de Santé Maladies Rares) have since been created in order to optimize the national organization. FSMRs gather and coordinate all the partners contributing to patient healthcare: CRMRs, diagnostic and research laboratories, social and medico-social structures, universities, associations, and any other partner or institution bringing complementary expertise to the field of RD. FSMRs coordinate the implementation of new diagnostic methods at the territorial level and, in particular, within the framework of the *PFMG2025*, the genomic healthcare pathways. Under the impulse of European patient organizations, notably EURORDIS, this organization into FSMRs was a model for the creation in 2017 of twenty-four thematic European reference networks for RD called European reference networks (ERN).

In parallel, the fight against cancer has also been structured since 2003 around 3 successive French national plans, supported at the highest level of the country by the French Presidents. These plans aimed to mobilize public health and research players around prevention, screening, organization of care, research, and support for patients and their families and the post-cancer period. Then, in 2019, the National Cancer Institute (INCa) was given the task of proposing a ten-year cancer-control strategy (2021-2030) encompassing different axes: (i) improving prevention; reducing side effects and improving quality of life; (ii) fighting cancers with poor prognosis; (iii) ensuring that everyone benefits from progress. In particular, a strong national organization has been structured for many years to ensure equal access to precision oncology throughout the territory, by providing access to both molecular profiling and innovative therapies targeting specific alterations. Two regional laboratory networks were set up to perform targeted molecular tumor and germline tests for all cancer patients in their region, mostly by panel-based sequencing. In parallel, INCa created and financially supports a network of certified early-phase centers (CLIP^2^) all over the country to improve patient access to unregistered molecular targeted agents. These centers design and conduct national and international early-phase clinical trials. Moreover, specific organizations have been adapted for the care of children with cancer, elderly patients as well as people with rare cancers.

# The *PFMG2025* research pilot projects

To implement genomic sequencing in clinical practices in France, *PFMG2025* initially addressed a number of technological, scientific, clinical and regulatory issues. The aim of the pilot projects was to test in research settings the *in situ* functionality and the coherence of the care pathway, including access to genome sequencing (GS) to experimental cohorts. The primary objective of these pilot projects was to identify bottlenecks and find practical solutions. One of the secondary objectives was to address medical and economic issues, in particular by analyzing the financial and social impact on families, as well as social information through human and social science studies. Four pilot projects were set up within the framework of the *PFMG2025*, involving the sequencing of specific cohorts of patients:

- In RD: DEFIDIAG trial (intellectual disability) (NCT04154891),
- In cancers: MULTIPLI project (MULTISARC trial: advanced/metastatic soft-tissue sarcoma),
- In common diseases: GLUCOGEN trial (diabetes),
- In the general population: POPGEN.

These ongoing studies are promoted by the Inserm (the French National Institute of Health and Medical Research).

DEFIDIAG (NCT04154891)

Intellectual disability (ID) affects between 1% and 3% of the general population. The main goal of DEFIDIAG is to compare the percentage of causal genetic diagnoses obtained by trio GS (including the patient and both parents) to the percentage obtained using the minimal reference strategy currently used in France (Fragile-X testing, chromosomal microarray analysis, and gene panel strategy including 44 ID genes) for patients with ID having their first clinical genetics consultation. Secondary objectives include detecting structural abnormalities, identifying other diagnoses and potentially new genes for transfer to research. Part of the study will focus on efficiency, i.e., the cost and effectiveness of the new strategy. The project also includes an economic, medical and psychosocial impact study to estimate the cost of potentially avoidable diagnostic errors using this technology and the impact of the technology on patient management. A qualitative study will look at how parents and patients perceive this analysis.^1-3^ From 01/01/2020 to 04/29/2022, 1,275 trio individuals with intellectual disability were enrolled (probands and both parents). The DEFIDIAG study was conducted in parallel to the selection of the pre-indication ‘Intellectual disability’ on 01/01/2020. To ensure patients’ inclusion in DEFIDIAG, it was collectively decided that patients were first included in the DEFIDIAG pilot project. The pre-indication ‘intellectual disability’ was so only opened more than two years later, after the last inclusion in the DEFIDIAG protocol. All patients benefited from GS. The global analysis of the study is ongoing and will be published soon independently.

MULTISARC (NCT03784014)

Soft-tissue sarcomas (STS) account for 1% of adult cancers. Despite well-managed locoregional treatment, 40% of patients relapse and develop metastases. Treatment options are limited, and the median survival rate for these patients remains low (between 12 and 18 months), with no improvement since the 1970s. Recent research has shown that genetic sequencing can identify an actionable genomic alteration in almost 50% of affected patients. MULTISARC is a non-blinded randomized multicentric trial assessing whether next-generation sequencing (NGS) can be conducted for a large proportion of metastatic STS participants within a reasonable timeframe. A key secondary objective is to determine whether an NGS-guided therapeutic strategy may improve the outcome of participants compared to a standard strategy involving the same treatment for all participants in terms of overall survival.^4^ From 10/16/2019 to 10/16/2023, 439 individuals with soft-tissue sarcoma were randomized and 10 targeted therapies were available for the clinical trial. Patients benefited from tumor exome sequencing and RNAseq following a phase of feasability.^5^

GLUCOGEN

Diabetes was chosen because its prevalence in the French population is 7.5% in the 20-79 age range, accounting for 22,953 diabetes-related deaths, notwithstanding an additional 8.25% with glucose intolerance who are at high risk of developing full diabetes. The primary objective of GLUCOGEN is to assess the contribution of GS coupled with a multidisciplinary meeting (MDM) to the diagnosis of atypical forms of diabetes compared to current diagnostic practices in a randomized trial. Secondary objectives are multiple: clinical, economic, psychosocial and exploratory. We plan to randomize 1,020 patients with atypical diabetes. They will benefit from genome sequencing. Applications for authorization are being submitted to the relevant authorities

POPGEN

The primary objective of POPGEN is to develop a reference panel of genome sequences of individuals, representative of the mainland French population, which could be used in genetic studies to highlight variants associated with diseases. The secondary objectives are (1) the imputation of genetic variants in the 6,000 genotyped individuals who will not be sequenced using the 4,000 GS to achieve a total sample size of 10,000 individuals, (2) the study of the population genetics mechanisms that have shaped the genomic diversity of the French population, and (3) the development of methods to select ancestry matched controls for association testing. From 03/31/2021 to 02/19/2022, 10,250 healthy volunteers from the CONSTANCES cohort were included. CONSTANCES is a large-scale French epidemiological study, that numbered around 220,000 volunteers, based on a representative sample of the French population aged 18-69 at the time of the invitation, and affiliated to the General Social Security Scheme. The CONSTANCES cohort systematically collects a variety of data to monitor the health status, morbidity, mortality, risk factors and socio-demographic characteristics of its participants. Genotyping was performed on all usable salivary DNA (9,772 individuals). Genome sequencing and data analysis of 4,000 individuals are in progress.

# Clinical ‘pre-indications’ selection, setting up of the genomic healthcare pathway

**The selection of clinical ‘pre-indications’**

The French health technology assessment agency (*Haute Autorité de Santé -* *HAS*), which regulates the transfer of innovations in the French healthcare system, has requested that the conditions for prescribing genome/exome/transcriptome sequencing meet well defined criteria for all patients. This required the definition of prescription criteria (clinical features, imaging, previous negative biological investigations, …) for each clinical indication and the setting up of an organization to ensure rigorous compliance with these criteria. These ones were defined in agreement with the national RD networks and the learned societies for cancer genetic predisposition (CGP) and cancer, and validated by the *HAS*. A working group led by *HAS* selected clinical 'pre-indications' through calls for applications for the national RD networks and the learned societies of oncology. So called pre-indications (i.e., clinical indication for the first years, which will undergo medi-economic analysis later) are progressively expanded. Three successive calls for applications to the RD health networks and the learned societies in CPG and oncology selected 70 pre-indications. In 2019, 2020, and 2022, 15, 47, and 8 indications have respectively been selected, with expected annual activity around 17,380 prescriptions in the field of RD/CPG and an estimated number of 12,300 cancer patients yearly. The expected annual activity was estimated by the pre-indication coordinators based on their knowledge of the prevalence and the number of affected patients in accordance with the national RD networks and the learned societies for cancer genetic predisposition (CGP) and cancer. Each pre-indication is coordinated by at least one clinician and one clinical biologist.

**The setting up of the genomic healthcare pathway**

The generic multidisciplinary genomic healthcare pathway comprised several successive stages : (1) a medical consultation to inform the patient, (2) an upstream multidisciplinary meeting (MDM) for RD/CGP or multidisciplinary tumor boards (MTBs) for cancers to ensure patient’s eligibility and validate the medical prescription, (3) a medical consultation to sign the consent and perform the electronic prescription,^6^ (4) blood sampling and samples destocking for tissues, (5) the genome, exome and RNA sequencing, (6) the clinical-biological interpretation by clinical biologists, (7) an optional clinical-biological interpretation meeting before writing the medical biology report, (8) a downstream MDM for treatment proposition, (9) a medical consultation to return genomic result to the patient and initiate management and more or less treatment depending on the result identified. The upstream MDMs for RD/CGP and MTBs for cancers were so made up of different doctors and biologists and coordinated by experts in these clinical pre-indications.

In cancers, 26 MTBs were created, including a network of 21 regional MTBs for adult patients with solid tumor and rare cancers. In the field of RD/CGP, 120 upstream thematic MDMs focusing on one or only a few clinical pre-indications were set up, including a network of 34 local thematic MDMs for 10/62 pre-indications with large active file, as well as 37 regional and 49 national thematic MDMs for the 52 others. For these 86 national and regional thematic MDMs, the prescriptions remained local in RD reference centers, but the validation of prescriptions was centralized. This organization quickly appeared too time-consuming for certain clinicians, such as clinical geneticists, who received patients daily with many different RD and therefore had to participate in many different MDMs to validate their prescriptions. Therefore, a nationwide network of 24 local non-thematic MDMs was established. A complementary two-tier organization was so set up: (1) the national network the national network of 24 local non-thematic MDMs coordinated by clinical geneticists to validate prescriptions for all pre-indications in simple clinical cases when patients met certain specific clinical criteria, defined in agreement with the national RD networks (for each clinical pre-indication, these criteria were available online for easy access by clinicians). (2) for complex cases, prescribers had to discuss and validate prescriptions during the thematic national or regional thematic MDMs.

To facilitate the genomic healthcare pathway, PFMG2025 created a new dedicated function, named “genomic pathway managers” and deployed a network of these 51 new health professionals. They were expected to have a master's degree and expertise as a genetic counsellor, clinical research associate (CRA), or engineer (master's degree or higher). Their missions consist of assisting and monitoring genomic prescriptions, training prescribers to use electronic prescription tools, managing tumor and/or blood sampling circuits and contributing to fix non-compliant files with *FMGlabs*.

Figure showing the complementary two-tier organization set up for MDMs in RD

This two-tier organization was rolled out nationwide in 2021 after a 6-month pilot phase in 8 different centers (https://pfmg2025.fr/professionnels/projet-pilote-rcp-fmg-mr-genomiques/). In 2022, 33% of the prescriptions without local thematic MDMs were validated by local non-thematic MDMs, reaching 48.3% in 2023. In total, 63% of prescriptions were validated by local thematic or non-thematic MDMs in 2022, reaching 71.4% in 2023.

# Ethical, Legal, and Social Issues

The implementation of genomic medicine in the French healthcare system involves taking into account the ethical and regulatory dimensions related to the provision of genetic tests and to the collection, storage and processing of patient data for healthcare provision as well as for secondary use for research purposes.

**The French regulatory framework for genomic medicine**

The *PFMG2025* falls within several regulatory frameworks. First of all, it must comply with the regulations related to the practice of clinical biology. As such, SeqOIA and AURAGEN have the status of clinical biology laboratories, which obliges them to meet regulatory requirements to ensure the quality of GS. Moreover, GS reports must be signed by a clinical biologist (*Article L. 6213-1 of the Public Health Code (PHC)*) or by a person who has obtained the authorization to practice clinical biology (*Article L. 6213-2 of the PHC*). In practice, a scientist who is neither a medical doctor or a pharmacist and who graduated after the promulgation of this law cannot exercise the functions of a clinical biologist and therefore sign GS reports.

In addition, due to its individual, familial, ethical and psychological consequences, clinical germline genetics is governed by an additional specific legal framework set by the French bioethics law revised in 2021. In the first place, the express consent of the person concerned, or of the persons exercising parental or legal authority, must always be obtained in writing before GS after the person has been informed. In parallel, as clinical GS providers, SeqOIA and AURAGEN must be authorized by their respective regional health agency, after advice from the Biomedicine Agency (*Article L .1131-2-1 of PHC*) and medical reports for RD/CGP pre-indications must be signed by a clinical molecular geneticist or biologist approved by the Biomedicine Agency according to specific conditions of training and experience (*Article L. 1131- 3 of PHC*). This approval is granted for a period of 5 years.

Finally, the storage and processing of patient data, in clinical as well as in research setting, is governed by the General Data Protection Regulation (GDPR).

**Consent forms and information sheets**

Specific consent models were drafted by a working group led by the French Ministry of Health and composed of health professionals, representatives of the ministries in charge of Health and Justice, health and research institutions (French National Cancer Institute, National Institute of Health and Medical Research, Biomedicine Agency) and patients. Their use for GS prescription within the framework of *PFMG2025* is mandatory. On the same document, the patient can also consent to the reanalysis of his data as part of the continuation of the diagnostic process, to different methods of informing his relatives, to the conservation of their samples and of their data for a secondary use for research. Moreover, cancer patients can consent to be informed in case of the identification of a germline cancer predisposition variant. Several consent models have been drafted in order to respond to different personal patient situations: for adults or minors, and in the case of RD or CPG pre-indications, for index cases, relatives, adults under guardianship, deceased minors and deceased fetuses.

The working group also drafted two information sheets for RD/CGP and cancer patients, which were translated into English, Arabic, Spanish and Turkish. These sheets were also simplified in order to adapt them to different levels of understanding of patients and to make them understandable by all. This was possible thanks to the setting up of a multidisciplinary working group, led by the Inserm College of Reviewers, bringing together health professionals, researchers, representatives of patient associations, lawyers, scientific mediators and graphic designers. This group reflected on how to address the person according to their level of understanding and developed both texts and illustrations. Thus, the initial information sheets were released in seven additional versions: two for adults (simplified and illustrated), three for children (classic, simplified and illustrated) and two for their parents (simplified and illustrated). These new sheets were translated into English and Arabic.

These information sheets are intended to evolve, more particularly to integrate the future reporting of incidental findings. A process for evaluating these sheets will also be set up, in order to take into account feedback from healthcare professionals or patients.

**Incidental findings**

The French bioethics laws in 2021 and the publication of the genetics decree at the end of 2023 provide for the reporting of incidental findings related to genetic testing. The Biomedicine Agency is currently drafting guidelines on the reporting of such data in order to harmonise medical practice. The *PFMG2025* will have to set up an organisation so that *FMGlabs* can return incidental findings in accordance with the recommendations soon to be issued by the Biomedicine Agency*.*

**Pharmacogenomics**

To date, French bioethics laws do not allow the search for secondary findings in genomic data. Only incidental findings can be returned to patients. It is therefore not currently permitted to actively search for pharmacogenetic variants that can be used preventively, in the absence of a complication linked to the use of a drug. However, this could be considered for pre-indications for which there are already known pharmacogenetic indications, such as epilepsy or certain cancers.

# CRefIX (Centre de Référence, d’Innovation, d’Expertise et de Transfert)

The implementation of a genomic medicine initiative integrated into the care pathway faces many technological challenges that must be tackled to ensure its successful deployment. In this context, *CRefIX* was created from the beginning of the *PFMG2025* initiative to identify and remove the main obstacles to its deployment. A second objective was to support the transfer in clinical practice of the most appropriate incremental or breakthrough developments in the highly evolving field of genomic sequencing, starting with genome, exome and transcriptomic analyses adapted. In the mid-term, *CRefIX*'s mission is to prepare for the inclusion of multi-omics technologies and analyses that will undoubtedly become the standard diagnostic approach in upcoming years. Indeed, some innovative technologies are being used commonly in research centres but are not yet ready for use in diagnostics, such as for long read sequencing for example. *CRefIX*’s goal is to ensure that patients will benefit as soon as possible from these technologies, without lowering diagnostic requirements such as data quality and repeatability. On the other hand, genomic and transcriptomic data generated through the initiative need to meet discovery research criteria, i.e., with as little bias as possible. Combining research and diagnostic requirements is a challenge that the *PFMG2025* is committed to overcoming, with help from the *CRefIX*. *CRefIX* is strategically located at the National Center for Human Genomics Research (CNRGH, Evry) in order to benefit from state-of-the-art expertise in genomic sequencing and to allow for the rapid transfer of knowledge and technology to the sequencing platforms. On 12/31/2023, in addition to the two co-directors, the *CRefIX* employed a total of 4 biologists, 3 bioinformaticians, and 2 operational and administrative assistants.

Beyond finding solutions to technological barriers, *CRefIX* has two other missions. The first is the development of biological standards to ensure the comparability of the results generated regardless of the *FMGlab* and regardless of the technical approaches (samples preparation methods, sequencer, bioinformatics pipelines, etc.). The second is, through its technological monitoring, to meet emerging companies developing products of potential interest for the program in order to identify relevant technologies and to contribute to the development of a medical genomics field in France. *CRefIX* is currently evaluating the best biological and bioinformatics strategies to optimize the use of DNA and RNA from paraffin-embedded biopsies to maximize the number of prescriptions for cancer patients, as although clinicians are strongly recommended to freeze samples, freezing is not systematically used across the country.^7^ Similarly, *CRefIX* is evaluating the usability of circulating tumor DNA in plasma as a tool not only for the discovery of driver mutations in patients in which tumor biopsy analysis may fail or tumor sample is not accessible. Regarding sequencers, *CRefIX* is currently evaluating new solutions combining a reduction of costs and an increase in productivity, including Illumina, MGI, Ultima, PAc bio and others, in order to advise *FMGlabs* on the best combination of investments while respecting their logistical and regulatory constraints, and ensuring a smooth transition with a minimum impact on the production flow. Finally, *CRefIX* establish contacts with other national genomic medicine initiatives and in particular, Genomics England to share experiences and promote data homogenization.

In practice, *CRefIX* began to evaluate the sequencing strategy that should be promoted for the first five years, proposed recommendations and common methods following international best practices, and offered technical support for the first two *FMGlabs*. These common protocols (library synthesis, sequencing reactions, bioinformatics analyses and genome interpretations) were evaluated by both *FMGlabs* before production and adapted for their use in production.

*CRefIX* and both *FMGlabs* agree on common protocols with a consensus reached to sequence paired-end short reads germline genome (depth 30-40X) and tumoral exome (depth 150-200X), genome (depth 60-80X) and transcriptome (64 million reads) in frozen tissues for oncology pre-indications, and to sequence paired-end short reads germline genome (depth 30-40X) in a trio or even in a duo approach as much as possible for RD.

# The first two *FMGlabs* (AURAGEN and SeqOIA)

The first two *FMGlabs* (clinical sequencing laboratories of the *PFMG2025*), SeqOIA and AURAGEN, were selected in 2017 as part of a call for applications launched by the French Ministry of Health, encouraging public-private partnerships.

Samples were collected by the same industrial subcontractor, from all mainland and overseas. Blood samples were sent to *FMGlabs* for RD/CGP, whereas frozen material of primary or metastatic tumors, as well as blood or bone marrow samples, were mainly sent for cancers.

SeqOIA, located in the *Ile-de-France* region, is a Health Cooperation Group (GCS) made up of one large university hospital (Assistance Publique – Hôpitaux de Paris) and two cancer centres (Institut Curie and Gustave Roussy). On 12/312023,, in addition to the clinical biologists, SeqOIA employed

1 biologist director, 1 bioinformatics director, 2 systems and network engineers, 1 software operation manager, 3 pipeline integration managers, 3 bioinformatics engineers, 1.7 datascientists, 4 IT developers, 2 genomic pathway managers,

7 technicians, and 1 quality manager. SeqOIA also used some subcontractors, details of which are available on request.

AURAGEN, located in the *Auvergne-Rhône-Alpes* region, is a GCS encompassing four university hospitals (Hospices Civils de Lyon, CHU de Grenoble, CHU de Saint-Etienne, CHU de Clermont-Ferrand), two cancer centres (Centre Lyon Bérard and centre Jean Perrin) and the Loire Cancer Institute. On 12/312023, in addition to the clinical molecular geneticists or biologists, AURAGEN employed : 0.3 biologist director ,1 bioinformatics director, 1,3 systems and network engineers, 0.5 pipeline integration managers, 3,5 bioinformatics engineers, 1 datascientist, 1,5 IT developers, 2,1 genomic pathway managers, 2 technical engineers, 5.5 technicians, 2.1 quality manager, 1 logistic officer , and 5 Administrative assistant AURAGEN also used some subcontractors, details of which are available on request.

Both *FMGlabs* have a generic activity, for RD/CGP and cancers, with an operational capacity of 6,000 index cases or 18,000 equivalent genomes per year and the objective of a target delivery time of 3-4 months for RD/CGP and 6-8 weeks for cancers. Until July 2023, there were no priorities based on disease type or patient. On this date, an urgent pathway was opened with a maximum time limit of 28 days for results for children with rare diseases in intensive care unit.

They have a status of clinical laboratories, with regulatory constraints and the requirement of authorizations from the competent authorities (regional health agency and biomedicine agency). Both *FMGlabs* have also entered into an ISO15189 accreditation process.

# Detailed common protocols used by both *FMGlabs*

This section details common protocols (library synthesis, sequencing reactions, bioinformatics analyses and genome interpretations) used by AURAGEN and SeqOIA in the fields of RD, cancer genetic predisposition (CPG) and cancers. Both *FMGlabs* performed germline genome sequencing for RD/CGP, and tumor and germline genome sequencing, and tumor exome and tumor RNAseq using frozen tissue samples for cancers. In tumors exome and genome sequencing are performed in parallel to enhance the sensitivity in detecting sub-clonal somatic variations where GS depth might be limitant compared to ES.

Data processing is performed on local high performance computing facilities using combinations of in-house and on-premise commercial software. French regulation requires certifications for health data management that limits cloud-based software and promotes the control of software and data. Health data management requires complete logging of data access, actions and decisions. Besides, each variant classification is accessible to the whole community of clinical biologists of each *FMGlab* for future reassessment and internal review, including consensual re-classification.

## Supplementary methods for RD/CGP

Prescription and sample management

After the informed consent is signed by the patients (and their relatives), clinical data were collected through the online electronic prescription tools using standardised clinical description based on HPO terms for each patient. Samples were collected by a unique industrial sub-contractor, including from overseas regions (following the territorial repartition) and brought to both *FMGlabs*. Most of the samples were fresh whole blood, with limited prescriptions relying on fetal frozen tissues or genomic DNA extracted by a third-party laboratory. Samples were transported at controlled temperature.

Nucleic acid extraction

Genomic DNA was extracted from whole blood using an automated QIAsymphony instrument (QIAGEN®, Germany), following the standard procedures. Genomic DNA was extracted from frozen tissues using a Maxwell RSC 48 instrument (Promega®), following the standard procedures. Quality was assessed using gel electrophoresis (Tapestation, Agilent or Fragment Analyzer System, Agilent) and quantity was measured by absorbance using a Tecan Infinite F200 Pro instrument. A genomic profile of each extracted DNA sample was defined using 48 or 96 genotyped common SNPs. Gender and intra-familial geno-coherence were assessed and the detected anomalies were solved before further processing of the samples.

Library preparation and sequencing

Standard procedures were applied. Briefly 4-5 μg of the DNA sample was fragmented using Covaris LE220 (Covaris Inc., Woburn, MA, USA) to obtain an average size of 450 bp DNA fragments. DNA samples were processed using the Illumina TruSeq DNA Polymerase Chain Reaction-Free Sample Preparation kit (Illumina Inc., San Diego, CA, USA) manually or on the Hamilton Microlab Star (Hamilton Robotics, Inc, Reno, NV, USA).

The final libraries were quantified using qPCR or low coverage sequencing. Sequencing experiments were performed on a NovaSeq6000 instrument (Illumina Inc., San Diego, CA, USA), with the 150 bp paired-end protocol using S1/S2/S4 flowcells. Sequencing libraries were pooled to target a median sequencing depth of 30-40 reads on the whole genome.

Bioinformatics analysis

Identity monitoring was performed using in-house scripts measuring a genotypic coherence matrix across individuals using common polymorphisms (including the previously determined SNPs). Cross-sample contamination was assessed using in-house scripts.

Quality assessment of the sequencing included (i) the sequencing by at least 20 reads of more than 90% of the autosomal primary diagnostic target, (ii) the controlled depth variability measured by a Variance/Median Ratio. It was determined that these criteria ensure high quality detection of SNVs, small indels, copy number variants, structural variants, short tandem repeats.

Raw BCL files were converted to FASTQ files using Bcl2fastq (v2.20.0) with standard parameters. FASTQ files were aligned to the GRCh38p13 reference genome using the BWA software (v0.7.17). Duplicates were marked using biobambam2/bammarkduplicates2 (v2.0.89). SNPs and indels were called using Haplotype Caller (GATK v4.2.0.0). Variants were annotated using the Gencode reference (Gencode 32, GCA_000001305.23), using combinations of ANNOVAR and VEP (v98.3 to 110.1), or SNPEff (the databases queried were SNPEff, 1000Genomes, gnomAD exomes, gnomAD genomes (v3.1), ClinVar (v201907 to v202401), COSMIC (coding and non-coding), dbscSNV, dbSNP, dbNSFP, and phastCons. Copy-number variations (CNV) and structural variants (SV) were detected using CNVnator (v0.4.1), Facet, WisecondorX, Lumpy and/or Manta (v1.6.0), then annotated by VEP (v98.3 to 110.1) or AnnotSV to which the following databases could have been added: Cytoband and COSMIC. Variants were presented to the interpreting practitioners through in-house web-interfaces (gLeaves, cutevariant,https://cutevariant.labsquare.org/).

French diagnostic regulation requires versioning of pipelines for medico-legal purposes of single exam traceability. Pipelines are routinely updated with a selected periodicity. Devoted procedures detail the tests are performed before the pipeline being production ready. Because of knowledge dynamics, annotations databases are updated more frequently than variant calling softwares.

Interpretation and reporting

Variants were reviewed and classified according to the ACMG-AMP guidelines.^8-11^ Variants considered as clinically relevant and related to the suspected disease were reported to the prescribers and subsequently to the families. If necessary, variants could be discussed by experienced multi-disciplinary teams in an optional clinical-biological interpretation meeting. The interpretation was not based on an in silico pre-defined target gene panel, but the analysis first focused on genes known to be involved in the pre-indication and/or phenotype, and then extended to the whole genome, both coding and non-coding regions.

When required, confirmatory analyses or further biochemical or functional confirmatory diagnostic tests were carried out in the already existing network of clinical laboratories in France.

## Supplementary methods for cancer

Prescription and sample management

After the informed consent is signed by the patients, the clinical cases were presented for approval in one of the initial molecular tumor boards (MTB). Samples were collected by a unique industrial sub-contractor, including from overseas regions (following the territorial repartition) and brought to both *FMGlabs*. Most of the samples were frozen material of the primary or metastatic tumors or nucleic acids extracted. Samples were transported at controlled temperature.

Nucleic acid extraction

DNA extraction: Constitutional genomic DNA was extracted from whole blood and purified on magnetic beads (Maxwell® RSC Whole Blood DNA Kit, PROMEGA) or using an automated QIAsymphony instrument (Qiagen®, Germany). Tumor genomic DNA was extracted and purified on magnetic beads (Maxwell® RSC Tissue DNA Kit, PROMEGA), either automatically (Maxwell® RSC System, PROMEGA) or manually (AllPrep DNA/RNA mini Kit, QIAGEN). Quality was assessed using gel electrophoresis (Tapestation®, Agilent® or Fragment Analyzer®, Agilent®) and quantity was measured by absorbance using a Tecan® Infinite F200 Pro instrument or the Quant-It™ INVITROGEN™ Spark™ TECAN® kit. A genomic barcode of each extracted DNA could have been defined using 48 or 96 genotyped common SNPs.

Tumor RNA was extracted from frozen material of the primary or metastatic tumors, and purified on magnetic beads (Maxwell® RSC Simply RNA tissue Kit, PROMEGA) either automatically (Maxwell® RSC System, PROMEGA) or manually (AllPrep DNA/RNA mini Kit, QIAGEN). Quantification and qualification of the nucleic acids could have been carried out on Fragment Analyzer^®^, Agilent^®^.

Library preparation and sequencing

Genome sequencing library preparation: DNA fragmentation was obtained by sonication (Covaris L220plus® COVARIS®). Library preparation was performed with or without amplification (respectively with the Nextera® DNA Flex Library Prep kit or the TruSeq® DNA PCR-Free kit, ILLUMINA®, or Ultra II End repair/A-tailing module and Ligation module (New England Biolabs®)) on a PLC (MICROLAB Star®, HAMILTON®) or manually. Size profiles are analyzed by capillary migration (TapeStation 4200®, Agilent®). The library was quantified by qPCR (Invitrogen™ Collibri™ Library Quantification Kit; QuantStudio™ 5 real Time PCR Systems, APPLIED BIOSYSTEMS).

Exome sequencing library preparation: Fragmentation was obtained by sonication (Covaris L220plus® COVARIS®). Size selection and subsequent purification steps were performed on magnetic beads (Sera-Mag magnetic beads; GE Healthcare). The preparation of the precapture library (NEBNext Ultra II End repair/A-tailing module & Ligation module; New England Biolabs) was amplified by PCR (KAPA Hifi HotStart ReadyMix; Roche) or using the SureSelect™ XT HS2 DNA System kit, AGILENT® on a PLC (MICROLAB Star®, HAMILTON®) or by single-plex hybridization (Twist Human Core Exome Kit +/- IntegraGen Custom v1; Twist BioScience). Size profiles could have been analyzed by capillary migration (TapeStation 4200®, AGILENT®). The final library was qualified by capillary migration (Fragment Analyzer, Agilent) and quantified by qPCR (NEBNext Custom 2X Library Quant Kit Master Mix, New England Biolabs; QuantStudio six Flex Real-Time PCR System, Life Technologies) or by spectrofluorometric assay (Quant-It™ Kit, INVITROGEN™, Spark™, TECAN®). Libraries were sequenced in ‘pair-end’ mode (2 series of 100 cycles) by SBS technology (Flow Cell S2; NovaSeq 6000; Illumina).

RNA sequencing library preparation: Stranded cDNA were generated by reverse transcription of messenger RNA after polyA capture (NEBNext® Poly(A) mRNA Magnetic Isolation Modules, NEBNext® Ultra II RNA First Strand Synthesis Module & Directional RNA Second Strand Module, New England Biolabs®).

The ribosomal RNA depletion step was performed onusing magnetic beads (Agencourt® RNAClean XP, BECKMAN COULTER), the other purification and size selection steps awere performed onusing magnetic beads (Agencourt® AMPure XP, BECKMAN COULTER or Sera-Mag magnetic beads; GE Healthcare). RNA-based libraries were prepared with amplification (TruSeq Stranded Total RNA H/M/R- Gold, ILLUMINA® or KAPA Hifi HotStart ReadyMix; Roche) on automated systems (MICROLAB Star®, Hamilton®) or manually. Size profiles were analyzed by capillary migration (TapeStation 4200®, Agilent®). The library was quantified by spectrofluorometric assay (Quant-It™ Kit, INVITROGEN™, Spark™, TECAN® or NEBNext Custom 2X Library Quant Kit Master Mix, New England Biolabs; QuantStudio six Flex Real-Time PCR System, Life Technologies).

Libraries sequencing: Libraries were pair-end sequenced (2 times 150 cycles) by SBS technology: "sequencing by synthesis" (NovaSeq® 6000, ILLUMINA®). Median depth target is: 40X for genome sequencing in normal tissue;70X for genome sequencing in tumoral tissue; 250X for exome sequencing in tumoral tissue. RNASeq target is 64 M. read pairs.

Bioinformatics analysis

Raw BCL files of the germline and tumor genome, tumor exome, tumor genome, and tumor transcriptome, whenever available, were converted to FASTQ by using Bcl2fastq (v2.20.0) with standard parameters. FASTQ files were further aligned to the GRCh38p13 reference genome using the BWA software (v0.7.17) for genome and exome and STAR (v2.6.1d) software for RNA-seq. Duplicates were marked by using biobambam2/bammarkduplicates2 (v2.0.89) or Picard MarkDuplicates (Picard Tools, 2.8.1). Somatic SNVs and short indels were called, on GS and ES, by using GATK4 (v4.2.0.0) Mutect2. Germline SNVs and short indels were called by using Platypus (v0.8.1) or GATK4 (v4.2.0.0). All variants were annotated by using VEP (v98.3 to 110.1) or SNPeff (4.3t) and SnpSift (4.3t) This could have included Gencode (v32), SNPEff (v4.3t), 1000Genomes (phase3, v2013-05-02), dbSNP (v152), gnomAD (r2.1), gnomAD exomes (v2.1.1), gnomAD genomes (v3), sift (v5.2.2), ClinVar (v201907 to v202401), dbscSNV (v1.1), dbSNP (v20180418), dbNSFP (v4.0), phastCons (v08-May-2015) and COSMIC (v89). Somatic variants were further used to compute Tumour Mutational Burden (TMB) and mutational signatures by using SigProfilerSingleSample (v0.0.0.27) with CosmicDB (v3.3). Germline and tumor genome alignment files were used to assess microsatellite instability (MSIsensor/msi (v0.6) using 2932 curated microsats and homopolymers sites from mSings tool. Structural variations (SV) were detected by using Manta (v1.6.0). CNV were detected by using Facets (v0.5.14) and in-house software Asdog (v2.5.0). Copy-number variations (CNV) could have been detected using, Facet (v0.5.14), WisecondorX (v1.1.5), then annotated by AnnotSV (v3.0.7) to which the following databases could have been added: Cytoband (Decembre 2013, USCS), COSMIC (v90). HRD score was computed (scarHRD_0.1.1). Arriba, STAR-Fusion, and FusionCatcher performed fusion calling independently.). CNV were further used to evaluate the Fraction of Genome Altered (FGA) as well as the number of Large Scale Transitions (LST) using in-house scripts. HRD scores were computed by using scarHRD (v0.1.1), CHORD (v2.02) and HRDetect (v2.4.1). RNAseq fusions were detected by using Arriba (v2.0.0), STAR-Fusion (v1.9.1) and FusionCatcher (v1.30). Transcripts expression were evaluated by using StringTie (v0.14.1) and Salmon (v2.0). All variants were further merged (Tars) and presented (Ascute/Expresso) to the interpreting practitioners through in-house web-interfaces.

French diagnostic regulation requires versioning of pipelines for medico-legal purposes of single exam traceability. Pipelines are routinely updated with a selected periodicity. Devoted procedures detail the tests are performed before the pipeline being production ready. Because of knowledge dynamics, annotations databases are updated more frequently than variant calling softwares.

Interpretation and reporting

Variants were reviewed and classified according to the ACMG-AMP guidelines.^7-10^ A first report summarizing data curation and variants clinically relevant was produced. It was discussed in regular multidisciplinary molecular board meetings according to the cancer pre-indication involving bioinformaticians, molecular biologists and pathologists who provided the expert interpretation and selected biologically relevant genomic alterations and/or fusion transcripts. The report was then discussed within one of the MTB for treatment recommendation and reported to the prescribers.

# Clinical pre-indications for genome sequencing in RD/CGP as part of the *PFMG2025* initiative

List of the 62 clinical pre-indications progressively selected in RD/CGP from 2019 to 2023 by *HAS*, with the date of selection, the place of genome sequencing in the genetic diagnostic strategy and the expected annual active file for each subgroup of pre-indications. From 04/01/2019 to 12/31/2023, are presented the number of prescriptions, the number and percentage of diagnostic reports returned to prescribers, the number of positive diagnoses, non-conclusive diagnoses with variants of unknown significance and negative diagnoses, and the diagnostic yield per subgroup of pre-indications in RD/CGP.

| **Clinical pre-indications in RD/CGP** | | | **French RD network or CPG society** | **Date of selection** | **GS place in the genetic diagnostic strategy** | **Number of complete prescriptions in 2023 / expected annual number of probands (%)** | **Total number of complete prescriptions from 04/01/2019 to 12/31/2023** | **Total number of diagnostic reports returned to the prescribers for the complete prescriptions from 04/01/2019 to 12/31/2023** | **Completeness rate for the complete prescriptions from 04/01/2019 to 12/31/2023**  **(%)** | **Positive diagnosis for the complete prescriptions from 04/01/2019 to 12/31/2023** | **Non-conclusive diagnosis with variants of unknown significance for the complete prescriptions from 04/01/2019 to 12/31/2023** | **Negative diagnosis for the complete prescriptions from 04/01/2019 to 12/31/2023** | **Diagnostic yield for the complete prescriptions from 04/01/2019 to 12/31/2023**  **(%)** |
| --- | --- | --- | --- | --- | --- | --- | --- | --- | --- | --- | --- | --- | --- |
| Malformations and neurodevelopmental disorders | | Developmental abnormalities, malformative syndromes and dysmorphic syndromes without intellectual disability | AnDDI-Rares | 01/01/2020 | +/- after TGA or/and array-CGH | 5,652/11,710  (48.3%) | 12,399 | 8,555 | 69% | 2,632 | 830 | 5,093 | 30.8% |
|  |  | Syndromic forms of RD with oral expression | TETECOU | 01/01/2020 | after panel sequencing |  |  |  |  |  |  |  |  |
|  |  | Ocular malformations | SENSGENE | 01/01/2020 | +/- after panel sequencing |  |  |  |  |  |  |  |  |
|  |  | Complex congenital heart defects | CARDIOGEN | 01/01/2020 | +/- after TGA or/and array-CGH |  |  |  |  |  |  |  |  |
|  |  | Congenital and very early malformations and diseases of the cerebellum and brainstem | DéfiScience | 01/01/2020 | +/- after TGA |  |  |  |  |  |  |  |  |
|  |  | Dysraphisms | AnDDI-Rares/ NEUROSPHINX | 01/01/2020 | first intention |  |  |  |  |  |  |  |  |
|  |  | Brain malformations | AnDDI-Rares/ DéfiScience | 01/01/2020 | +/- after panel sequencing |  |  |  |  |  |  |  |  |
|  |  | Intellectual disability | AnDDI-Rares/ DéfiScience | 01/01/2020 | +/- after TGA or/and array-CGH |  |  |  |  |  |  |  |  |
|  |  | Drug-resistant epilepsies | DéfiScience | 01/01/2020 | after panel sequencing |  |  |  |  |  |  |  |  |
|  |  | Syndromic schizophrenia | DéfiScience | 01/01/2020 | after array-CGH |  |  |  |  |  |  |  |  |
|  |  | Autism Spectrum and Developmental Disorders without intellectual disability | DéfiScience | 01/01/2020 | first intention |  |  |  |  |  |  |  |  |
| Central nervous system disorders | | Leukodystrophies | BRAIN-TEAM | 01/01/2019 | +/- after panel sequencing | 473/800 (59.1%) | 1,236 | 832 | 67,31% | 317 | 83 | 432 | 38,1% |
|  |  | Dystonia or rare abnormal movements in young people | BRAIN-TEAM | 01/01/2020 | first intention |  |  |  |  |  |  |  |  |
|  |  | Hereditary ataxias in young people | BRAIN-TEAM | 01/01/2020 | +/- after TGA |  |  |  |  |  |  |  |  |
|  |  | Hereditary spastic paraparesis in young people | BRAIN-TEAM | 01/01/2020 | after panel sequencing |  |  |  |  |  |  |  |  |
|  |  | Neurodegenerative cognitive diseases and disorders in young people and/or families | BRAIN-TEAM | 01/01/2020 | +/- after TGA |  |  |  |  |  |  |  |  |
|  |  | Neurodegeneration by intracerebral iron accumulation | BRAIN-TEAM | 01/01/2020 | after panel sequencing |  |  |  |  |  |  |  |  |
|  |  | Brain calcifications | BRAIN-TEAM | 04/01/2022 | +/- after panel sequencing |  |  |  |  |  |  |  |  |
|  |  | Rare cerebrovascular diseases | BRAIN-TEAM | 04/01/2022 | +/- after panel sequencing |  |  |  |  |  |  |  |  |
| Neuromuscular diseases | | Peripheral neonatal hypotonia suspected of neuromuscular disease | FILNEMUS | 01/01/2020 | after TGA | 265/230 (115.2%) | 472 | 213 | 45,1% | 68 | 33 | 112 | 31.9% |
|  |  | Myopathies | FILNEMUS | 01/01/2020 | after TGA and/or panel sequencing |  |  |  |  |  |  |  |  |
|  |  | Hereditary peripheral neuropathies | FILNEMUS | 04/01/2022 | after TGA and/or panel sequencing |  |  |  |  |  |  |  |  |
|  |  | Amyotrophic lateral sclerosis | FISLAN | 04/01/2022 | after TGA |  |  |  |  |  |  |  |  |
| Sensory disorders | | Early-onset deafness | SENSGENE | 01/01/2020 | +/- after TGA | 637/1,200 (53.1%) | 1,684 | 1,002 | 59.5% | 406 | 93 | 503 | 40.5% |
|  |  | Hereditary retinal dystrophies | SENSGENE | 01/01/2020 | first intention |  |  |  |  |  |  |  |  |
| Cardiac diseases | | Familial cardiomyopathies | CARDIOGEN | 01/01/2020 | after TGA | 58/200 (29%) | 169 | 134 | 79,3% | 14 | 21 | 99 | 10,4% |
|  |  | Hereditary rhythm disorders | CARDIOGEN | 01/01/2020 | after TGA |  |  |  |  |  |  |  |  |
| Vascular diseases | | Rendu-Osler disease | FAVA-Multi | 01/01/2020 | after panel sequencing | 61/440 (13.9%) | 135 | 93 | 68.9% | 10 | 7 | 76 | 10.8% |
|  |  | Superficial arteriovenous and CNS malformations with aggressive potential | FAVA-Multi | 01/01/2020 | after panel sequencing |  |  |  |  |  |  |  |  |
|  |  | Medium-calibre artery diseases | FAVA-Multi | 01/01/2020 | after panel sequencing |  |  |  |  |  |  |  |  |
|  |  | Marfan syndrome and related conditions, familial forms of thoracic aortic aneurysms | FAVA-Multi | 01/01/2020 | after panel sequencing |  |  |  |  |  |  |  |  |
|  |  | Primary lymphoedemas | FAVA-Multi | 04/01/2022 | +/- after panel sequencing |  |  |  |  |  |  |  |  |
| Rare lung diseases | | | RESPIFIL | 01/01/2020 | after panel sequencing | 25/35  (71.4%) | 45 | 17 | 37.8% | 3 | 7 | 7 | 17.6% |
| Diabetes | | Rare diabetes in young people and lipoatrophic diabetes | FIRENDO | 01/01/2020 | after panel sequencing | 4/35  (11.4%) | 20 | 19 | 95.0% | 0 | 2 | 17 | 0.0% |
|  |  | Neonatal diabetes | FIRENDO | 01/01/2020 | after panel sequencing |  |  |  |  |  |  |  |  |
| Endocrine disorders | | Severe abnormalities of sexual differentiation of gonadal and hypothalamic-pituitary origin | FIRENDO | 01/01/2019 | after TGA and/or panel sequencing | 65/220 (29.5%) | 204 | 83 | 40.7% | 20 | 13 | 50 | 24.1% |
|  |  | Combined pituitary deficiencies | FIRENDO | 01/01/2020 | after panel sequencing |  |  |  |  |  |  |  |  |
|  |  | Cushing's syndrome with bilateral nodular adrenal hyperplasia | FIRENDO | 01/01/2020 | after panel sequencing |  |  |  |  |  |  |  |  |
|  |  | Pituitary hormonal hypersecretions | FIRENDO | 01/01/2020 | after panel sequencing |  |  |  |  |  |  |  |  |
|  |  | Thyroid axis dysfunction | FIRENDO | 01/01/2020 | after panel sequencing |  |  |  |  |  |  |  |  |
| Metabolic diseases | | Inherited metabolic diseases | G2M | 01/01/2019 | after panel sequencing | 138/300 (46%) | 407 | 283 | 69,5% | 91 | 27 | 165 | 31.0% |
|  |  | Mitochondrial diseases | FILNEMUS | 01/01/2019 | after TGA and/or panel sequencing |  |  |  |  |  |  |  |  |
|  |  | Rare pathologies of phospho-calcium metabolism or dental mineralisation | OSCAR | 01/01/2020 | after panel sequencing |  |  |  |  |  |  |  |  |
| Fertility disorders | | Premature ovarian failure | FIRENDO | 01/01/2019 | after panel sequencing | 36/1,060 (3.4%) | 96 | 74 | 77.1% | 11 | 6 | 57 | 14.9% |
|  |  | Rare male infertilities | FIRENDO | 04/01/2022 | after TGA |  |  |  |  |  |  |  |  |
| Gastro-hepato-enteric diseases | | Congenital enteropathies in young children | FIMATHO | 01/01/2020 | +/- after panel sequencing | 43/110 (39.1%) | 86 | 46 | 53.5% | 9 | 8 | 29 | 19.6% |
|  |  | Severe paediatric liver diseases | FILFOIE | 01/01/2020 | +/- after panel sequencing |  |  |  |  |  |  |  |  |
|  |  | Genetic chronic pancreatitis | FIMATHO | 04/01/2022 | after panel sequencing |  |  |  |  |  |  |  |  |
| Chronic kidney disease | | | ORKID | 01/01/2019 | +/- after panel sequencing | 131/100 (131%) | 397 | 227 | 57.2% | 55 | 22 | 150 | 24.2% |
| Hematological diseases | | Bone marrow aplasias and hypoplasias | MaRIH | 01/01/2020 | after TGA and/or panel sequencing | 35/200 (17.5%) | 133 | 117 | 88% | 24 | 16 | 77 | 20.5% |
|  |  | Constitutional red blood cell diseases | MCGRE | 01/01/2020 | after TGA and/or panel sequencing |  |  |  |  |  |  |  |  |
|  |  | Severe chronic neutropenias | MaRIH | 01/01/2020 | after panel sequencing |  |  |  |  |  |  |  |  |
|  |  | Hemostasis diseases | MHEMO | 01/01/2020 | +/- after panel sequencing |  |  |  |  |  |  |  |  |
|  |  | Histiocytoses without BRAFV600E mutation | MaRIH | 01/01/2020 | first intention |  |  |  |  |  |  |  |  |
| Immunological and autoinflammatory diseases | | Autoimmune and autoinflammatory monogenic diseases | FAI2R | 01/01/2019 | +/- after TGA | 81/140 (57.9%) | 175 | 104 | 59.4% | 16 | 10 | 78 | 15.4% |
|  |  | Hereditary immune deficiencies | MaRIH | 01/01/2020 | after TGA and/or panel sequencing |  |  |  |  |  |  |  |  |
|  |  | Hereditary bradykinin angiodema | MaRIH | 04/01/2022 | after TGA and/or panel sequencing |  |  |  |  |  |  |  |  |
| Bone and joint diseases | | Constitutional bone diseases | OSCAR | 01/01/2019 | +/- after panel sequencing | 284/350 (81.1%) | 831 | 643 | 77.4% | 156 | 88 | 399 | 24.3% |
|  |  | Syndromes with major joint hyperlaxity, without intellectual disability* | OSCAR | 04/01/2022 | +/- after panel sequencing |  |  |  |  |  |  |  |  |
| Rare skin diseases | | | FIMARAD | 01/01/2020 | +/- after panel sequencing | 64/100 (64%) | 157 | 95 | 60.5% | 4 | 3 | 48 | 46.3% |
| Cancer genetic predisposition | | Cancers with a particularly severe family history | GGC | 01/01/2019 | after panel sequencing | 84/150 (56%) | 280 | 200 | 71.4% | 19 | 20 | 161 | 9.5% |
|  |  | Cancers with « extreme » tumor phenotype and no family history | GGC | 01/01/2019 | after panel sequencing |  |  |  |  |  |  |  |  |
| TOTAL | | - | - | - | - | 8,136/17,380 (46.8%) | 18,926 | 12,737 | 67,3% | 3,895 | 1,289 | 7,553 | 30.6% |
|  | GGC: *Groupe Génétique et Cancer*, TGA: targeted genetic analysis (unique or multiple single gene analysis) | | | | | | | | | | | | |

# Clinical pre-indications for GS/ES/RNAseq in cancers as part of the *PFMG2025* initiative

List of the 8 clinical pre-indications selected in cancers from 2019 to 2023 by *HAS*, with the total number of complete prescriptions in 2023 and the expected annual expected patients per pre-indications as well as the total number of complete prescriptions from 04/01/2019 to 12/31/2023.

| Clinical pre-indications in cancers | French Cancers societies | Date of labelling | Number of complete prescriptions in 2023  (expected annual number of patients) | Total number of complete prescriptions from 04/01/2019 to 12/31/2023 |
| --- | --- | --- | --- | --- |
| Advanced cancers with 1^st^ line treatment failure | GFCO/SCOPP | 01/01/2019 | 491/6,000  (8.2%) | 1,246 |
| Cancers of unknown primary | GFCO/SCOPP | 01/01/2020 | 55/2,000  (2.8%) | 117 |
| Pediatric cancers and leukemia at diagnosis | SFCE | 01/01/2020 | 127/1,250  (10.2%) | 193 |
| Pediatric cancers and leukemia with treatment failure | SFCE | 01/01/2019 | 201/500  (40.2%) | 631 |
| Rare cancers | GFCO/SCOPP | 01/01/2020 | 326/2,000  (16.3%) | 817 |
| Relapsed or refractory acute leukemia, eligible for a curative treatment | GBMHM | 01/01/2019 | 242/250  (96.8%) | 332 |
| Relapsed or refractory Diffuse Large B cell lymphoma (DLBCL) | GBMHM/LYSA | 01/01/2020 | 8/250  (3.2%) | 17 |
| Lymphoma with an uncertain diagnosis | GBMHM/LYSA | 01/01/2020 | 6/50  (12%) | 14 |
| TOTAL |  |  | 1,456/12,300  (11.8%) | 3,367 |

# Evolution of the number of prescribers and clinical biologists with geographical distribution of prescriptions and clinical biologists over time

Evolution of the number of prescribers (purple) and clinical biologists (mauve) between 12/31/2020 and 12/31/2023

(A)


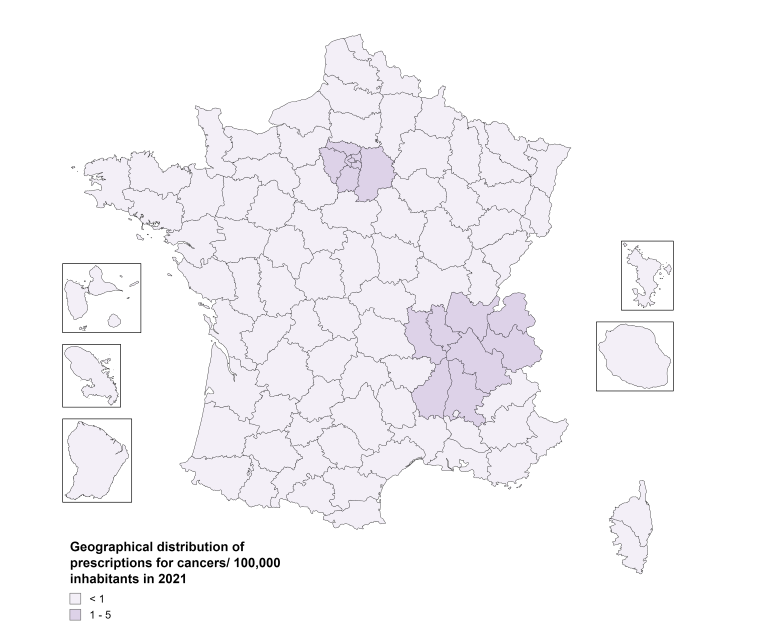

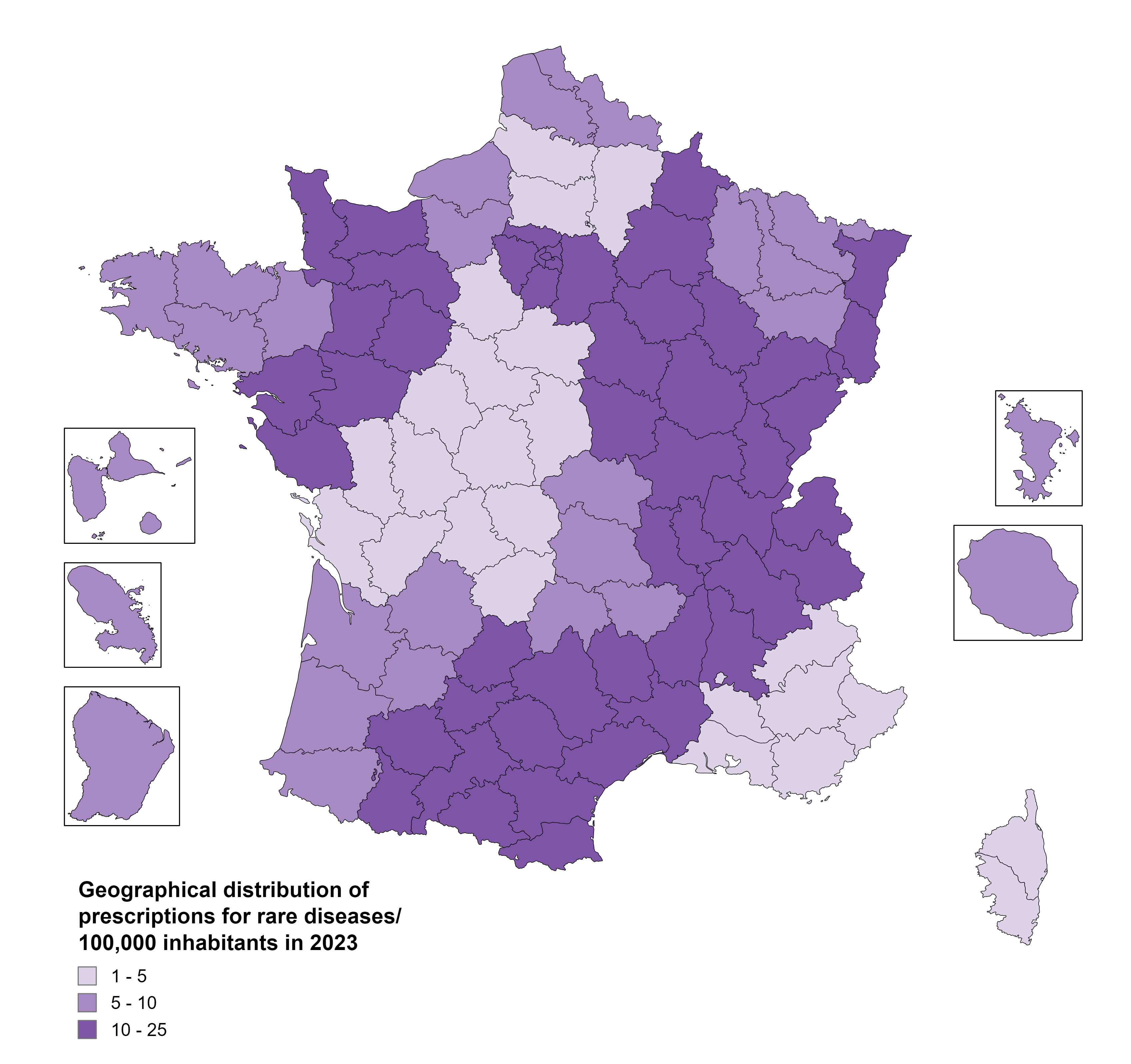

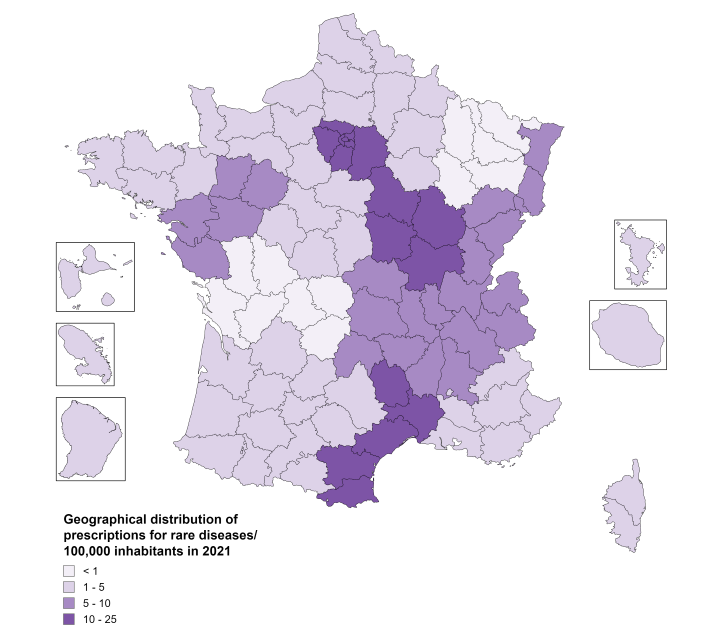

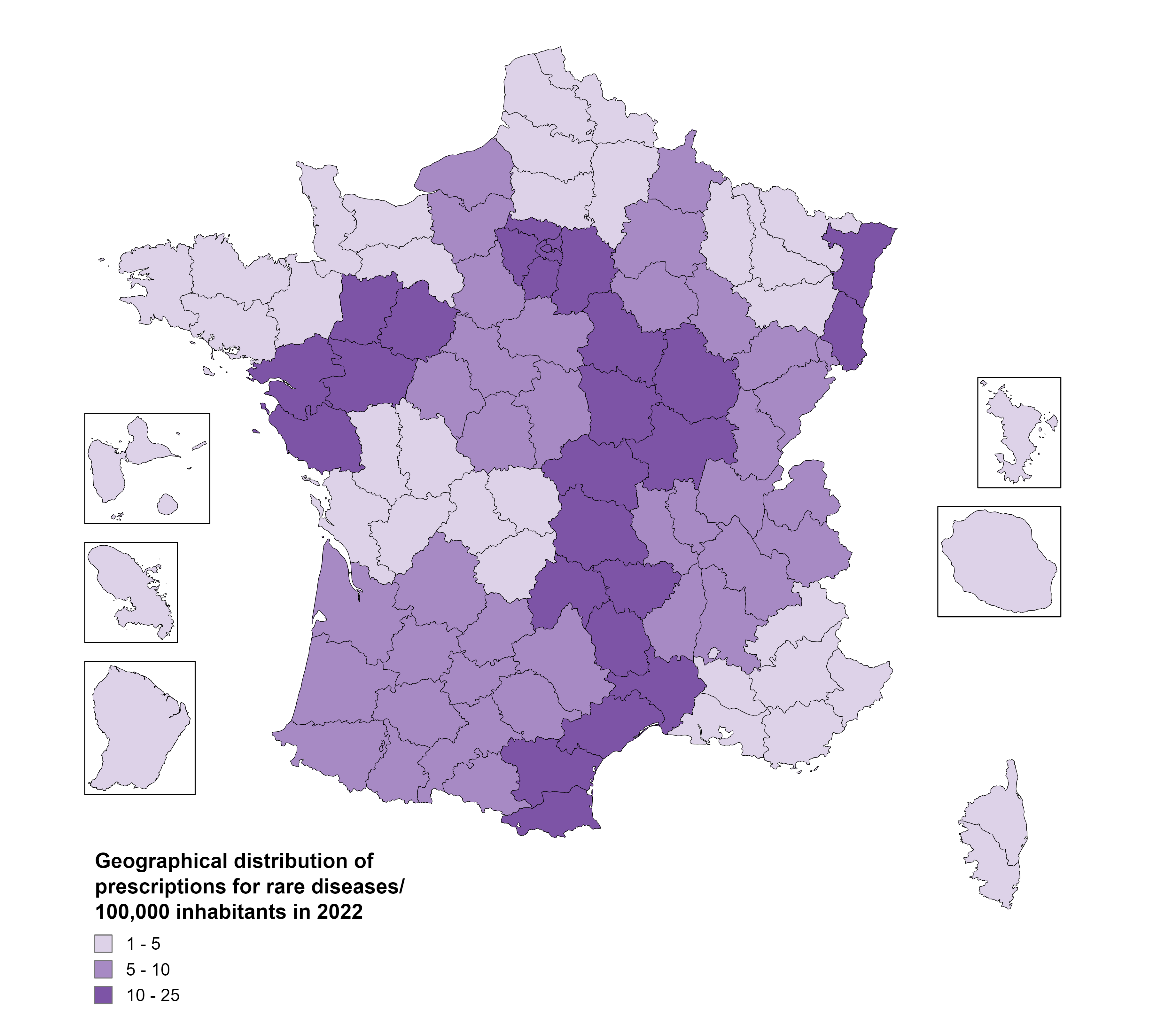

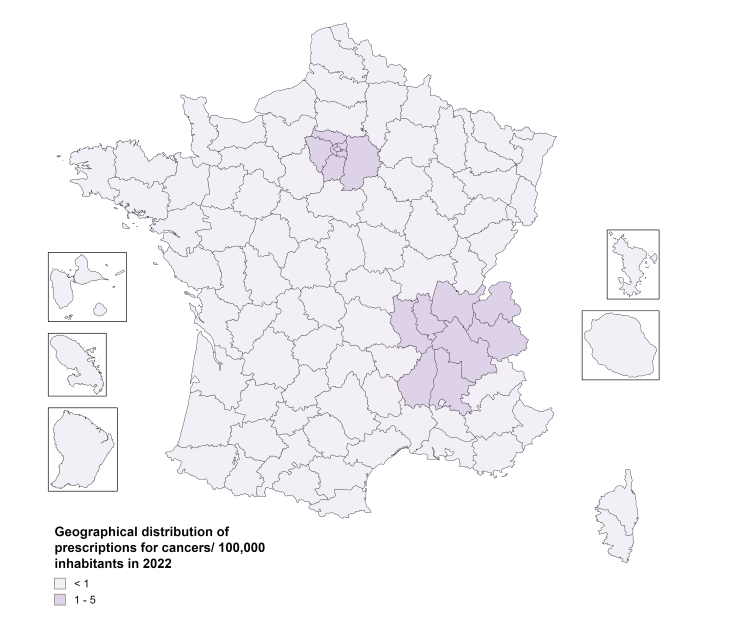

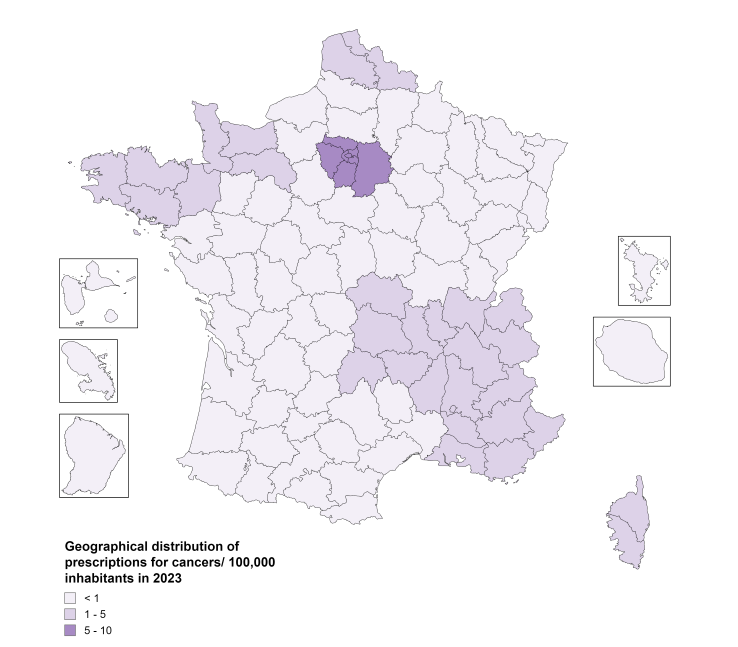

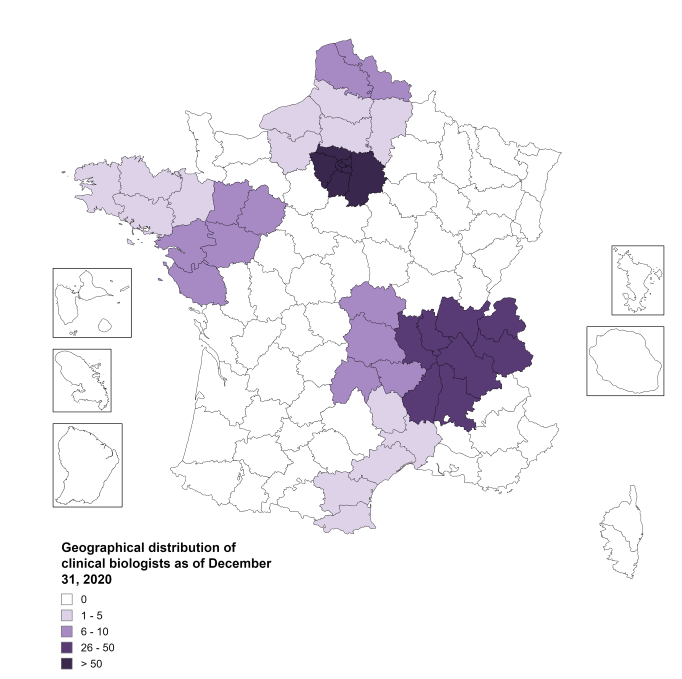

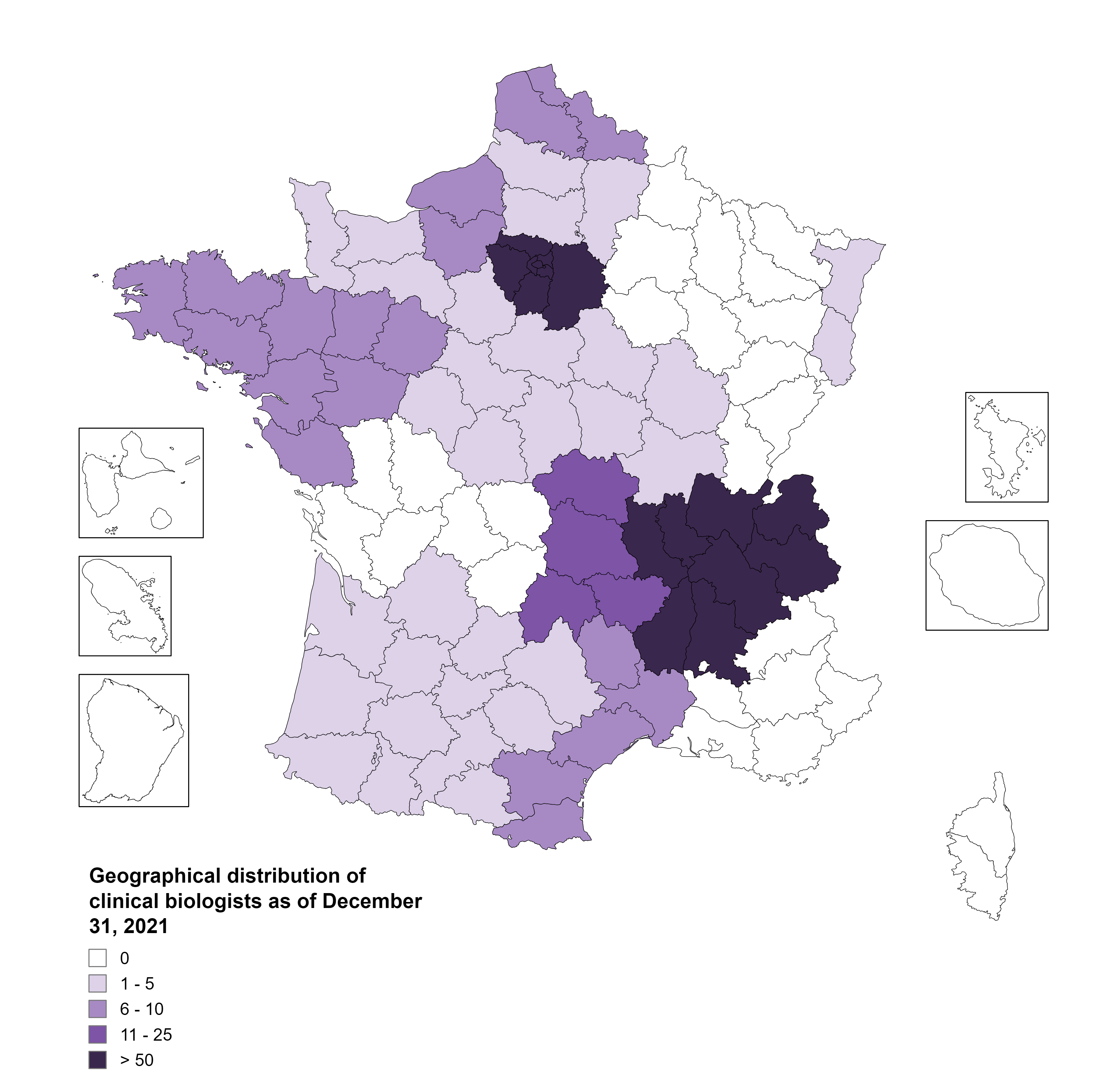

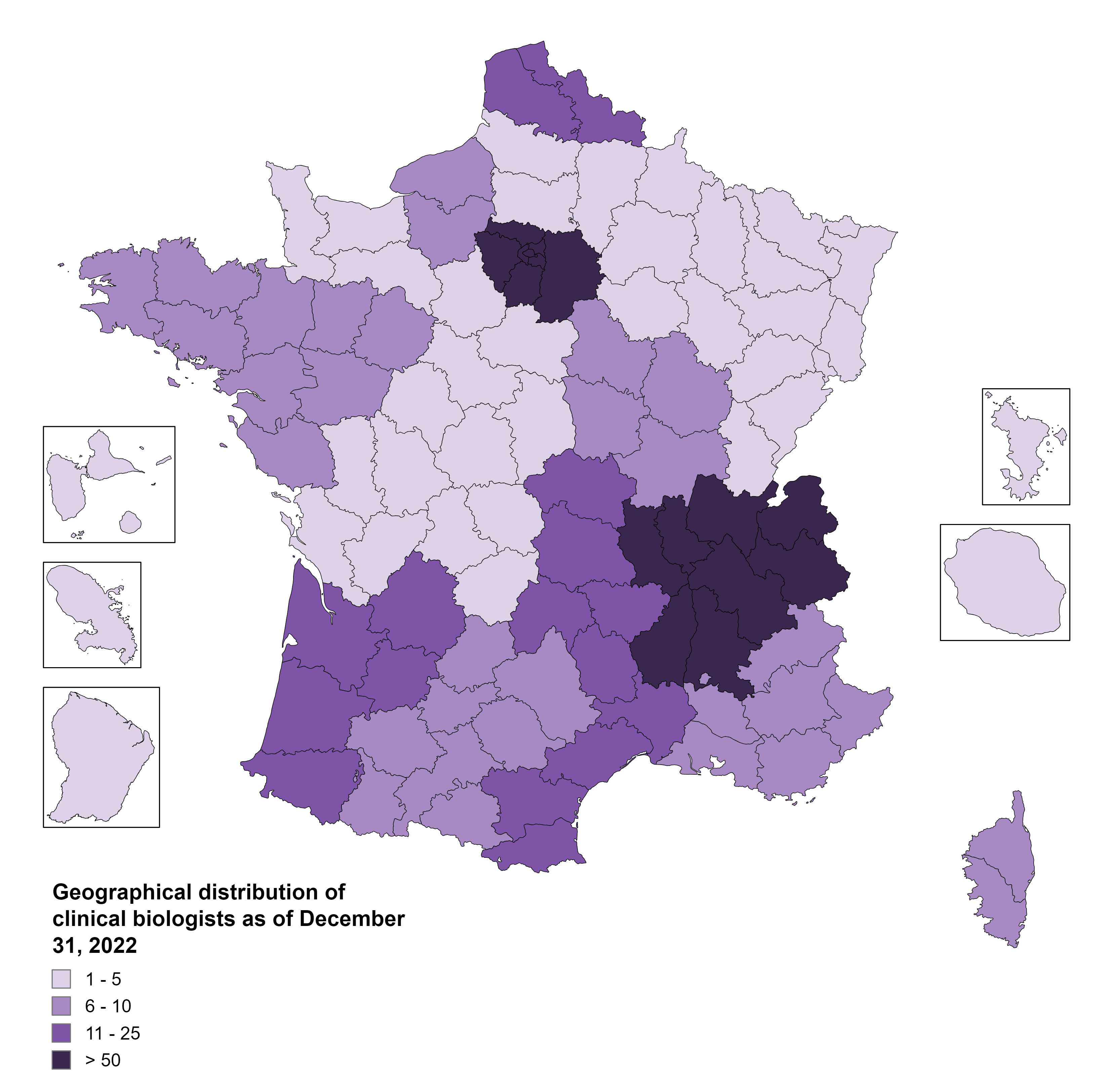


(B)

(C)

(A) Geographic distribution of prescriptions for RD/CGP / 100,000 inhabitants in 2021 (left), 2022 (middle) and 2023 (right), showing an increase in prescriptions in the majority of French regions; (B) Geographical distribution of prescriptions for cancer / 100,000 inhabitants in 2021 (left), 2022 (middle) and 2023 (right), showing a moderate increase in prescriptions in only a few French regions; (C) Geographical distribution of the clinical biologists for RD/CGP and cancers on 12/31/2020 (left), 2021 (middle) and 2023 (right), showing an increase in biologists in the majority of French regions, also outside the GCS AURAGEN and SeqOIA.

# Detailed results provided by both *FMGlabs*

## Supplementary results for RD/CGP

Number of complete prescriptions received in 2023 compared with the expected number of annual prescriptions by subgroup of RD/CGP pre-indications. (CNS: central ervous system, GHE: gastro-hepato-enteric diseases, IAI: immunological and autoinflammatory diseases, MND: malformations and/or neurodevelopmental disorders)

Repartition of the 18,926 complete prescriptions by subgroup of RD/CGP pre-indications from 04/01/2019 to 12/31/2023. (CNS: central nervous system, GHE: gastro-hepato-enteric diseases, IAI: immunological and autoinflammatory diseases, MND: malformations and/or neurodevelopmental disorders)

Delivery time (grey bars) between receiving the prescription at *FMGlabs* and returning the report to the prescribers of the 12,737 returned results from the 18,926 complete prescriptions per subgroup of RD/CGP pre-indications from 04/01/2019 to 12/31/2023 and completeness rate as of 12/31/2023 (purple lines), ranged from the smallest cohort (rare lung diseases, N= 17) to the largest cohort (MND disorders, N=8,555) (CNS: central nervous system, GHE: gastro-hepato-enteric diseases, IAI: immunological and autoinflammatory diseases, MND: malformations and/or neurodevelopmental disorders)

Completeness rate as of 12/31/2023 (grey bars) and diagnostic yield (purple lines), ranged from the smallest to the largest completeness rate (CNS: central nervous system, GHE: gastro-hepato-enteric diseases, IAI: immunological and autoinflammatory diseases, MND: malformations and/or neurodevelopmental disorders)

Moving towards GS as a first line diagnostic in several medical conditions including rare disorders is expected. To anticipate the diagnostic yield of this shift in the diagnostic strategy, we considered two periods for the major prescribed pre-indications. The first period, ranged from the start until 03/30/2021, with over 97% of retuned results and detailed elements of prescribed tests before GS. The second period ranged from the start until 12/31/2022, with at least 200 and 75% of returned results. We compared the diagnostic yield of published cohorts,^12-16^ although this might be limited by missing data on possible genetic tests performed before GS. Diagnostic yields were overall similar in *PFMG2025*.

|  | For complete prescriptions on 03/30/2021 | | | | | For complete prescriptions on 12/31/2022 | | | | |
| --- | --- | --- | --- | --- | --- | --- | --- | --- | --- | --- |
| Clinical pre-indications for RD | Complete prescriptions | Results returned to the prescribers | Positive diagnosis | Complete-ness rate | Diagnostic yied | Complete prescriptions | Results returned to the prescribers | Positive diagnosis | Complete-ness rate | Diagnostic yied |
| Developmental abnormalities, malformative syndromes and dysmorphic syndromes without intellectual disability | 1212 | 1205 | 326 | 99,4% | 27,1 % | 3233 | 2881 | 850 | 89,1% | 29,5 % |
| Intellectual disability | 181 | 179 | 77 | 98,9% | 43,0 % | 2264 | 1857 | 667 | 82,0% | 35,9 % |
| Constitutional bone diseases | 246 | 244 | 60 | 99,2% | 24,6 % | 543 | 531 | 126 | 97,8% | 23,7 % |
| Hereditary retinal dystrophies | 155 | 154 | 91 | 99,4% | 59,1 % | 561 | 423 | 257 | 75,4% | 60,8 % |
| Early-onset deafness | 188 | 183 | 45 | 97,3% | 24,6 % | 485 | 453 | 102 | 93,4% | 22,5 % |

Completeness rates et diagnostic yields for the 5 main pre-indications during the first period, ranged from the start until 06/30/2022 and the second period ranged from the start until 12/31/2022.

Clinical and biological data (presence or absence of previous genetic data) were retrospectively collected for the 2,734 consecutive prescriptions in RD/CGP performed from 04/01/2019 to 06/30/2021 (requiring the sequencing of 2,734 unrelated probands and 5,461 relatives). However, these results are representative of the initial period of setting-up of the healthcare pathways and the two first *FMGlabs*. Family compositions were heterogeneous, although trios mostly included affected probands and their unaffected parents (85.5%); duplex strategies (5.3%) were more diverse unaffected/affected parent – proband, concordant or discordant sib pairs and more distant relatives. Solo (1.4%) and families with 4 individuals and more (7.8%) were also present.

Overall, GS results were available in 2,683/2,734 (98.1% of completeness) with a causal diagnosis reached in 771/2,683 probands (28,7%) who carried disease causing variants explaining the phenotypic features. Of these diagnoses, 84.9% (655) were made on the basis of only SNVs or indels, including 123 intronic variants, 11.9% (92) on the basis of only SV (including CNV), 2.8% (22) on a combination of SNV and SV, and 0.25% (2) on STR affecting well-established disease genes. After excluding variant of unknown significance, a double diagnosis was identified in 17/771 probands (2.2%) including 1 double unknown inheritance, 1 *de novo* and unknown inheritance, 3 *de novo* and inherited (2 autosomal dominant and 1 autosomal recessive), 5 double *de novo*, 7 double inherited (1 double hemizygous, 2 double autosomal homozygous, 3 autosomal double heterozygous, and 1 hemizygous and heterozygous). Parental segregation was available in 673/754 unique causal diagnoses showing a large heterogeneity in inheritance with sporadic autosomal heterozygous variant largely predominant (49.9%).

| Inheritance in probands with unique causal diagnosis | | N=673 |
| --- | --- | --- |
| Sporadic : 371/673 (55.1%) | Autosomal heterozygous variant : 336/371 | 49.9% |
|  | X-linked heterozygous variant: 21/371 | 3.1% |
|  | Hemizygous variants : 14/371 | 2.1% |
| Autosomal recessive : 184/673 (27.3%) | Homozygous : 97/184 | 14.4% |
|  | Compound heterozygous variants : 87/184 | 12.9% |
| Parentally inherited : 117/673 (17.4%) | Autosomal dominant variant : 95/117 | 14.1% |
|  | X-linked hemizygous variant : 22/117 | 3.3% |
| Mitochondrial : 1/673 (0.1%) | - | 0.1% |

Prevalence of the different modes of inheritance in probands with unique causal diagnosis

## Supplementary results for cancers

Number of complete prescriptions received in 2023 compared with the expected number of annual prescriptions by cancers pre-indication

Delivery time between receiving the prescription at *FMGlabs* and returning the report to the prescribers of the 3,109 returned results from the 3,367 complete prescriptions by cancers pre-indications from 04/01/2019 to 12/31/2023

# Four clinical cases of interest in RD diagnosed by genome sequencing

Case 1 (APHP– SeqOIA – Dr C. Mignot, Dr B. Keren)

This boy was the third child born to healthy parents and had two healthy sisters. Pyelectasia was found by fetal US during the second trimester pregnancy. The child was born full term with a weight of 3310 g, length 48 cm, OFC 34 cm, and Apgar scores 10/10. Ungual aplasia of both halluces was noted at birth. Transient hypercalcemia with normal plasma alkaline phosphatase was found at birth. During the first year of life, the boy had choking episodes requiring the use of a feeding tube and severe constipation with a diagnosis of pediatric intestinal pseudo-obstruction. He had several life-threatening episodes of respiratory distress due to aspirations or viral infections and showed marked global developmental delay. The child developed epilepsy at 1 year old with a febrile status epilepticus. When examined in the genetics unit at age of 4 years old, weight, height and OFC were within normal range. The patient had profound intellectual disability, he was unable to speak, to hold his head and to sit up, had poor eye contact. Brain MRI did not show anomalies. The epilepsy remained pharmaco-resistant and multifocal epileptiform anomalies were seen on EEGs. Chromosomal micro-array was normal. Trio-exome sequencing (proband and parents) revealed the hemizygous ChrX(GRCh38):g.74741831G>A,ENST00000055682.12:c.2726C>T,p.(Ala909Val) missense variant of uncertain significance in NEXMIF (MIM*300524) and the single pathogenic heterozygous, paternally inherited, Chr18(GRCh38):g.62140447A>C, ENST00000640252.2:c.996T>G,p.(Ile332Met) missense variant in *PIGN* (MIM*606097) with autosomal recessive condition. The latter had been previously reported as pathogenic. Genome sequencing disclosed the heterozygous, maternally inherited, seq[GRCh38]del(18)(q21.33q21.33), chr18:g. 62152641_62157701del variant. The discovery of this intragenic *PIGN* deletion *in trans* of the previously *PIGN* missense variant led to the diagnosis of PIGN-related recessive encephalopathy (MIM#614080).

Case 2 (CHU Grenoble – AURAGEN – Pr J.Thevenon, Pr C.Coutton)

The proband was a male, third child of non-consanguineous healthy parents. Family history was unremarkable. Pregnancy was detected only at 22WG. At 25WG, ultrasonography identified polyhydramnios (HP:0001561), short femur length (HP:0011428), enlarged kidneys (HP:0000105) and micropenis (HP:0000054). Fetal MRI only identified aplasia of the olfactory bulbs (HP:0032466) and lateral ventricular asymmetry (HP:0100960). Fetal karyotype was normal (46,XY) and 15q11q13 locus methylation study was unremarkable. Chromosomal micro-array evidenced a 121-kb duplication including the exons 4, 5, 6 and 7 of the GPC3 gene (chrX:132,766,827-132,888,087, hg38) inherited from the mother. The predicted effect of this a priori intragenic duplication was uncertain and thus was classified as a variant of uncertain significance in this prenatal context. Birth was induced at 38WG. Birth weight was 3990g (96th centile), height (83th centile) and head circumference 36.5cm (94th centile), with thus macrosomia (HP:0001520). Clinical examination confirmed micropenis and also noticed macroglossia (HP:0000158), hepatosplenomegaly (HP:0001433) and mild axial hypotonia (HP:0008936). Morphological findings were: macrostomia (HP:0000154), broad nasal ridge (HP:0000431), anteverted nares (HP:0000463), furrowed tongue (HP:0000221), small and large hands (HP:0004279 ; HP:0001176) and supernumerary nipple (HP:0002558). Chest X-ray showed twelfth rib hypoplasia (HP:0006668). At 4 months he had recurrent respiratory infections and poor sucking due to macroglossia. At latest examination at 10 months development was satisfying, he could sit alone, crawl and babble. Height and weight were at the 25th centile and head circumference at mean. Alpha foetoprotein and abdominal ultrasonography were normal. Trio genome sequencing was performed with a specific focus on *GPC3* gene (MIM* 300037). Genome sequencing revealed a complex rearrangement in the Xq26.2q27.2 region inherited from the mother. This rearrangement included a large genomic paracentric inversion of 9.26 Mb between the regions Xq26.2 and Xq27.2 (chrX(GRCh38):g.133577393_142836500inv). The centromeric breakpoint disrupted the *GPC3* gene integrity. In addition, we found two inverted duplications inserted at both breakpoints. At the centromeric breakpoint (Xq26.2), we found an 184-kb inverted duplication (chrX:g.142652620_142836500) encompassing no known protein-coding genes. At the telomeric breakpoint (Xq27.2), an inverted duplication of 225 kb including the four exons of GPC3 (chrX(GRCh38):g.133577394_133802937) was inserted corresponding to the duplicated segment previously identified with array-CGH. Overall, this complex genomic rearrangement interrupted the GPC3 gene leading to its haploinsufficiency responsible for the Simpson-Golabi-Behmel syndrome (MIM#312870) compatible with most of clinical features described in the patient.

We also identified a small heterozygous deletion of 4.7 kb in the 4p14 region encompassing the two last exons of the *KLB* gene (chr4(GRCh38):g.39445122_39449668del), inherited from the father. Heterozygous loss-of-function *KLB* variants have been associated with the Kallmann syndrome (PMID: 28754744). This deletion may thus participate to explain the micropenis and the olfactory bulb aplasia reported in the patient.

Case 3 (CHU La Réunion – AURAGEN – Dr O. Dunand, Dr L. Michel)

This old boy was born to healthy parents originated from Comoro Islands/Mayotte and had one younger maternal half-brother with similar phenotype and additional lenticonus. He presented with bilateral hearing loss, macroscopic hematuria, and renal insufficiency. At age 8 years, the phenotype was consistent with Alport syndrome. Renal biopsy was inconclusive, but skin biopsy showed the absence of collagen IV alpha5 chain. Alport panel sequencing (COL4A3, COL4A4, COL4A5) was negative.

Quator-genome sequencing (proband, affected mother, affected half-brother and non-affected brother) disclosed the segregating deep intronic variant in COL4A5 (MIM*303630) intron 33, chrX(GRCh38):g.108623992A>G, ENST00000328300.11:c.2918-244A>G. *In silico* scores predicted the strengthening of a cryptic acceptor splice site (SpliceAI=Acceptor gain:0.60) with 2 preexisting strong donor sites downstream (SpliceAI=donor gain:0.49). Transcriptional analysis, performed on new skin biopsy in proband and RT-PCR on cultured fibroblasts compared with control fibroblasts, revealed the creation of an out of phase pseudo-exon, using the splice acceptor site enhanced by the variant and the strongest of the preexisting donor sites with a total effect on splicing. This evidenced that the deep intronic variant induced the introduction in the cDNA of an out of phase pseudo-exon allowing its classification as pathogenic (ACMG 5) and solved diagnosis in COL4A5-related X-linked Alport syndrome (MIM#301050).

The proband benefited from kidney transplantation at age 21 years.

During the same time, nephrotic proteinuria and hematuria were discovered incidentally during an infectious episode in a female child originated also from Comoro Islands/Mayotte and with familial history of Alport syndrome. Her father presented with deafness, ocular symptoms, and renal transplantation. Renal biopsy evidenced minimal-change disease consistent with the hypothesis of X-linked Alport even if alpha 2 and 5 chains of collagen IV were normally distributed. Alport/nephrotic syndrome panel sequencing was negative in proband and father.

Trio-genome sequencing (proband and parents) identified the same segregating deep intronic variant in intron 33, chrX(GRCh38):g.108623992A>G,ENST00000328300.11:c.2918-244A>G as described in the previous family.

Case 4 (CHU La Réunion – AURAGEN – Dr O. Dunand, Dr L. Michel)

This boy was the second child born to healthy parents originated from La Reunion Island and had one healthy sister. Enlarged hyperechoic kidneys with oligoamnios was found by fetal US at 27 weeks of gestation. The male newborn presented with severe neonatal respiratory distress, transient renal insufficiency, and severe hypertension. Abdominal ultrasound revealed also asymptomatic biliary microcysts. The phenotype was consistent with autosomal recessive polycystic kidney disease (ARPKD). Ciliopathy panel sequencing revealed a single heterozygous, paternally inherited, missense variant classified as variant of unknown significance, chr6(GRCh38):g.51755535T>C,ENST00000340994.4:c.7400T>C,p.(Leu2467Pro), in *PKHD1* (MIM*606702) with autosomal recessive condition. In a new pregnancy, fetal US revealed a recurrence of antenatal symptoms. The male newborn presented with less severe phenotype (transient respiratory distress).

Trio-genome sequencing (proband and parents) disclosed the deep heterozygous, maternally inherited, intronic variant in PKHD1 intron 55 chr6(GRCh38):g.51870590A>G,ENST00000340994.4:c.8643-597A>G *in trans* of the previously identified PKHD1 VUS. *In silico* scores predicted the creation of a strong acceptor splice site (SpliceAI Acceptor gain:0.99) with preexisting donor site downstream (SpliceAI donor gain:0.65). Familial Sanger sequencing segregation was consistent: affected younger brother was compound heterozygous for both variants and non-affected older sister only heterozygous for the missense variant. In vitro analysis using minigene confirmed the creation of an out-of-phase pseudo-exon using both predicted splice sites in intron 55 with a total effect on splicing. This allowed the classification or re-classification of both variants as pathogenic (ACMG5) and solved diagnosis in autosomal recessive PKHD1-related polycystic kidney disease (MIM#263200).

# The 78 causal diagnoses identified after negative exome sequencing among the 2,734 first consecutive prescriptions for RD/CGP

| genes | OMIM number | variant types | genominc variant | cDNA variant | protein variant | inheritance | Explanation of causal diagnosis identified by GS after negative ES |
| --- | --- | --- | --- | --- | --- | --- | --- |
| *ADCY5* | 600293 | exonic SNV | chr3:g.123352464G>A | ENST00000462833.6:c.1252C>T | ENSP00000419361.1:p.(Arg418Trp) | heterozygous, sporadic | Insufficient coverage or incapacity of interpreting gene/variant as pathogenic at the time of ES |
| *ARID1B* | 614556 | CNV | chr6:g.157040001_157133000del | NA | NA | heterozygous, sporadic | Structural variant not reported by ES and array-CGH |
| *ARL6IP1* | 607669 | exonic SNV | chr16:g.18798759G>A | ENST00000304414.12:c.112C>T | ENSP00000306788.7:p.(Arg38Ter) | homozygous | Insufficient coverage or incapacity of interpreting gene/variant as pathogenic at the time of ES |
| *ASPM* | 605481 | exonic SNV  exonic SNV | chr1:g.197143525G>A  chr1:g.197121989C>A | ENST00000367409.9:c.727C>T  ENST00000367409.9:c.3796G>T | ENSP00000356379.4:p.(Arg243Ter)  ENSP00000356379.4:p.(Glu1266Ter) | compound heterozygous | Insufficient coverage or incapacity of interpreting gene/variant as pathogenic at the time of ES |
| *ATAD3A* | 612316 | non-exonic SNV  CNV | chr1:g.1526536G>C chr1:g.1484701_1519450del | ENST00000378756.7:c.1337+5G>C  NA | NA  NA | compound heterozygous | Insufficient coverage or incapacity of interpreting gene/variant as pathogenic at the time of ES |
| *ATAD3A* | 612316 | inframe deletion  CNV | chr1:g.1534014_1534016del  chr1:g.1481801_1519200del;1497680_1502954del | ENST00000378756.8:c.1703_1705del  NA | ENSP00000368031.3:p.(Lys568del)  NA | compound heterozygous | Insufficient coverage or incapacity of interpreting gene/variant as pathogenic at the time of ES |
| *ATP1A3* | 182350 | exonic SNV | chr19:g.41968902C>T | ENST00000648268.1:c.2702G>A | ENSP00000498113.1:p.(Arg901Lys) | heterozygous, sporadic | Insufficient coverage or incapacity of interpreting gene/variant as pathogenic at the time of ES |
| *BMP4* | 112262 | exonic SNV | chr14:g.53950726A>C | ENST00000245451.9:c.533T>G | ENSP00000245451.4:p.(Ile178Arg) | heterozygous, inherited | Insufficient coverage or incapacity of interpreting gene/variant as pathogenic at the time of ES |
| *CASK* | 300172 | intragenic CNV | chrX:g.41609262_41616693del | ENST00000378163.7:c.1033+5924_1155+642del | NA | heterozygous, sporadic | Structural variant not reported by ES and array-CGH |
| *CCDC186*  *MYO1H* | 619249  614636 | non-exonic SNV  exonic SNV | chr10:g.114132185C>G  chr12:g.109415551C>T | ENST00000369287.8:c.1656-1G>C  ENST00000310903.10:c.1528C>T | NA  ENSP00000439182.2:p.(Arg510Ter) | homozygous  homozygous | At least one non-coding pathogenic variant not captured by classical ES capture kits |
| *CHD3* | 602120 | exonic SNV | chr17:g.7902972A>C | ENST00000330494.12:c.3406A>C | ENSP00000332628.7:p.(Thr1136Pro) | heterozygous, sporadic | Insufficient coverage or incapacity of interpreting gene/variant as pathogenic at the time of ES |
| *CHKB* | 612395 | exonic SNV | chr22:g.50581810C>G | ENST00000406938.3:c.386G>C | ENSP00000384400.3:p.(Arg129Pro) | homozygous | Insufficient coverage or incapacity of interpreting gene/variant as pathogenic at the time of ES |
| *CIROP* | 619703 | exonic SNV  exonic SNV | chr14:g.23101380T>C  chr14:g.23101322C>T | ENST00000637218.2:c.1661A>G  ENST00000637218.2:c.1719G>A | ENSP00000489869.1:p.(Tyr554Cys)  ENSP00000489869.1:p.(Trp573Ter) | compound heterozygous | Insufficient coverage or incapacity of interpreting gene/variant as pathogenic at the time of ES |
| *CPAMD8* | 608841 | non-exonic indel  intragenic CNV | chr19:g.16977366_16977369del  chr19.g.16974327_16975440del | ENST00000443236.7:c.1899+1_1899+4del  ENST00000443236.7:c.1909-171_2070+781del | NA  NA | compound heterozygous | Structural variant not reported by ES and array-CGH |
| *CRYBB2* | 123620 | exonic SNV | chr22:g.25231737T>G | ENST00000398215.3:c.583T>G | ENSP00000381273.2:p.(Trp195Gly) | heterozygous, inherited | Insufficient coverage or incapacity of interpreting gene/variant as pathogenic at the time of ES |
| *DPYSL5* | 608383 | exonic SNV | chr2:g.26940038C>G | ENST00000288699.11:c.955C>G | ENSP00000288699.6:p.(Leu319Val) | heterozygous, sporadic | Insufficient coverage or incapacity of interpreting gene/variant as pathogenic at the time of ES |
| *DYRK1A* | 600855 | exonic indel | chr21:37490311-37490313del | ENST00000647188.2:c.775_776del | ENSP00000339659.9:p.(Leu259fs) | heterozygous, sporadic | Insufficient coverage or incapacity of interpreting gene/variant as pathogenic at the time of ES |
| *EBF3* | 607407 | exonic SNV | chr10:g.129873547G>A | ENST00000440978.2:c.686C>T | ENSP00000387543.2:p.(Ser229Leu) | heterozygous, sporadic | Insufficient coverage or incapacity of interpreting gene/variant as pathogenic at the time of ES |
| *ECHS1* | 602292 | exonic SNV  exonic SNV | chr10:g.133370578C>T  chr10:g.133369929A>T | ENST00000368547.4:c.268G>A  ENST00000368547.4:c.389T>A | ENSP00000357535.3:p.(Gly90Arg)  ENSP00000357535.3:p.(Val130Asp) | compound heterozygous | Insufficient coverage or incapacity of interpreting gene/variant as pathogenic at the time of ES |
| *FANCM*  *CRIPT* | 609644  604594 | exonic SNV  exonic indel | chr14:g.45198718C>T  chr2:g.46619676del | ENST00000267430.10:c.5791C>T  ENST00000238892.4:c.132del | ENSP00000267430.5:p.(Arg1931Ter)  ENSP00000238892.3:p.(Ala45GlnfsTer86) | homozygous  homozygous | Insufficient coverage or incapacity of interpreting gene/variant as pathogenic at the time of ES |
| *FBXO11* | 607871 | exonic SNV | chr2:g.47839449T>C | ENST00000403359.8:c.412A>G | ENSP00000384823.4:p.(Arg138Gly) | heterozygous, sporadic | Insufficient coverage or incapacity of interpreting gene/variant as pathogenic at the time of ES |
| *FGF14* | 601515 | non-exonic STR | - | - | - | heterozygous, inherited | At least one non-coding pathogenic variant not captured by classical ES capture kits |
| *FOXP1* | 605515 | intragenic CNV | chr3:g.70986537_70990562del | ENST00000649528.3:c.1063-2485_1146+1457del | NA | heterozygous, sporadic | Structural variant not reported not by ES and reported as VUS by array-CGH |
| *GATA6* | 601656 | exonic SNV | chr18:g.22181517G>A | ENST00000269216.10:c.1367G>A | ENSP00000269216.3:p.(Arg456His) | heterozygous, sporadic | Insufficient coverage or incapacity of interpreting gene/variant as pathogenic at the time of ES |
| *GNB1* | 139380 | exonic SNV | chr1:g.1806504T>C | ENST00000378609.9:c.238A>G | ENSP00000367872.3:p.(Ile80Val) | heterozygous, sporadic | Insufficient coverage or incapacity of interpreting gene/variant as pathogenic at the time of ES |
| *GNPNAT1* | 616510 | exonic SNV | chr14:g.52781903C>T | ENST00000216410.8:c.226G>A | ENSP00000216410.3:p.(Glu76Lys) | homozygous | Insufficient coverage or incapacity of interpreting gene/variant as pathogenic at the time of ES |
| *HACE1* | 610876 | exonic indel  CNV | chr6:g.104744197_104744199del  chr6:g.104794375_104805190del | ENST00000262903.9:c.2475_2477del  ENST00000262903.9:c.617+6121_923+1204del | ENSP00000262903.4:p.(Glu827del)  NA | compound heterozygous | Structural variant not reported by ES and reported as VUS by array-CGH |
| *HEXA* | 606869 | exonic indel | chr15:g.72349153_72349155del | ENST00000268097.10:c.915_917del | ENSP00000268097.6:p.(Phe305del) | homozygous | Insufficient coverage or incapacity of interpreting gene/variant as pathogenic at the time of ES |
| *HK1* | 142600 | exonic SNV | chr10:g.69398629C>T | ENST00000359426.7:c.2410C>T | ENSP00000352398.6:p.(Leu804Phe) | heterozygous, sporadic | Insufficient coverage or incapacity of interpreting gene/variant as pathogenic at the time of ES |
| *HNRNPK* | 600712 | exonic indel | chr9:g.83972159_83972161del | ENST00000376263.8:c.676_678del | ENSP00000365439.3:p.(Asp226del) | heterozygous, sporadic | Insufficient coverage or incapacity of interpreting gene/variant as pathogenic at the time of ES |
| *HYCC1* | 610531 | non-exonic SNV  SV | chr7:g.22977340C>A  [GRCh38] 3q24-q25.1(149185892-149229277)x0 | ENST00000432176.7:c.414+1G>T  NA | NA  NA | heterozygous inherited  unknown | Non-coding pathogenic variant not captured by classical ES capture kits and structural variant not detected by ES (array-CGH not performed previously) |
| *IFIH1* | 606951 | exonic SNV | chr2:g.162272377C>T | ENST00000649979.2:c.2465G>A | ENSP00000497271.1:p.(Arg822Gln) | heterozygous, sporadic | Insufficient coverage or incapacity of interpreting gene/variant as pathogenic at the time of ES |
| *IFIH1* | 606951 | exonic SNV | chr2:g.162272377C>T | ENST00000649979.2:c.2465G>A | ENSP00000497271.1:p.(Arg822Gln) | heterozygous, inherited | Insufficient coverage or incapacity of interpreting gene/variant as pathogenic at the time of ES |
| *IKBKG* | 300248 | non-exonic SNV | chrX:g.154560384A>C | ENST00000594239.6:c.519-23A>C | NA | heterozygous, sporadic | At least one non-coding pathogenic variant not captured by classical ES capture kits |
| *ITPR2* | 600144 | Exonic SNV | chr12:g.26716227T>C | ENST00000381340.8:c.541A>G | ENSP00000370744.3:p.(Lys181Glu) | heterozygous, sporadic | Insufficient coverage or incapacity of interpreting gene/variant as pathogenic at the time of ES |
| *JAK2* | 147796 | exonic SNV | chr9:g.5073770G>T | ENST00000381652.4:c.1849G>T | ENSP00000371067.4:p.(Val617Phe) | heterozygous, sporadic | Insufficient coverage or incapacity of interpreting gene/variant as pathogenic at the time of ES |
| *KAT6B* | 605880 | exonic SNV | chr10:g.75022006G>A | ENST00000287239.10:c.3147G>A | ENSP00000287239.4:p.(Pro1049=) | heterozygous, sporadic | Insufficient coverage or incapacity of interpreting gene/variant as pathogenic at the time of ES |
| *KIF1A* | 601255 | non-exonic SNV | chr2:g.240741241G>A | ENST00000498729.7:c.3749+28G>A | NA | heterozygous, sporadic | At least one non-coding pathogenic variant not captured by classical ES capture kits |
| *KMT2D* | 602113 | exonic SNV | chr12:g.49024611C>G | ENST00000301067.12:c.16019G>C | ENSP00000301067.7:p.(Arg5340Pro) | heterozygous, sporadic | Insufficient coverage or incapacity of interpreting gene/variant as pathogenic at the time of ES |
| *LHCGR* | 152790 | CNV | chr2:g.48665776_48698069  chr2:g.48707059_48711185 | NA  NA | NA  NA | compound heterozygous | Structural variant not reported by ES and reported as VUS by array-CGH |
| *MAF* | 177075 | exonic SNV | chr16:g.79599026T>C | ENST00000326043.5:c.877A>G | ENSP00000327048.4:p.(Arg293Gly) | heterozygous, sporadic | Insufficient coverage or incapacity of interpreting gene/variant as pathogenic at the time of ES |
| *NF1* | 613113 | CNV | chr17:g.30652001_32068000del | NA | NA | heterozygous, sporadic | Structural variant not reported by ES and array-CGH |
| *NRXN1* | 600565 | CNV | chr2:g.49939001_49980000del | NA | NA | heterozygous, inherited | Structural variant not reported by ES (array-CGH not performed previously) |
| *NUP188* | 615587 | non exonic SNV  exonic indel | chr9:g.128998624G>A  chr9:g.128983347_128983348delinsG | ENST00000372577.2:c.3515+1G>A  ENST00000372577.2:c.1851_1852delinsG | NA  ENSP00000361658.2:p.(Cys617TrpfsTer2) | compound heterozygous | At least one non-coding pathogenic variant not captured by classical ES capture kits |
| *PCYT2* | 602679 | non exonic SNV | chr17:g.81905377C>T | ENST00000538936.7:c.969+5G>A | NA | homozygous | At least one non-coding pathogenic variant not captured by classical ES capture kits |
| *PHF8* | 300560 | non exonic SNV | chrX:g.53987945C>G | ENST00000338154.11:c.1731-1G>C | NA | hemizygous, inherited | At least one non-coding pathogenic variant not captured by classical ES capture kits |
| *PHOX2B* | 603851 | exonic indel | chr4:g.41746005_41746114del | ENST00000226382.4:c.651_760del | ENSP00000226382.2:p.(Pro218SerfsTer105) | heterozygous, sporadic | Insufficient coverage or incapacity of interpreting gene/variant as pathogenic at the time of ES |
| *PIGN* | 606097 | CNV  exonic SNV | chr18:g. 62152641_62157701del  chr18:g.62140447A>C | NA  ENST00000640252.2:c.996T>G | NA  ENSP00000492233.1:p.(Ile332Met) | compound heterozygous | Structural variant not reported by ES and array-CGH |
| *PKD1* | 601313 | exonic indel | chr16:g.2103784_2103790dup | ENST00000262304.9:c.8271_8277dup | ENSP00000262304.4:p.(Met2760ProfsTer64) | heterozygous, inherited | Insufficient coverage or incapacity of interpreting gene/variant as pathogenic at the time of ES |
| *PKD2* | 173910 | CNV | chr4:g.88043856_88050314del | NA | NA | heterozygous, sporadic | Structural variant not reported by ES and array-CGH |
| *POLR3A* | 614258 | non-exonic SNV  exonic SNV | chr10:g.78009682C>G  chr10:g.77993286A>C | ENST00000372371.8:c.1771-7C>G  ENST00000372371.8:c.2698T>G | NA  ENSP00000361446.3:p.(Phe900Val) | compound heterozygous | At least one non-coding pathogenic variant not captured by classical ES capture kits |
| *POU4F1* | 601632 | exonic indel | chr13:g.78602404_78602414del | ENST00000377208.7:c.264_274del | NA | heterozygous, sporadic | Insufficient coverage or incapacity of interpreting gene/variant as pathogenic at the time of ES |
| *PQBP1* | 300463 | CNV | chrX:g.48887895_48888770del | NA | NA | heterozygous, sporadic | Structural variant not reported by ES and array-CGH |
| *RAC1* | 602048 | exonic SNV | chr7:g.6392028C>T | ENST00000348035.9:c.212C>T | ENSP00000258737.7:p.(Ser71Phe) | heterozygous, sporadic | Insufficient coverage or incapacity of interpreting gene/variant as pathogenic at the time of ES |
| *RELN* | 600514 | exonic indel | chr7:g.103651748_103651765del | ENST00000428762.6:c.1790_1807del | ENSP00000392423.1:p.(His597_Ser602del) | heterozygous, sporadic | Insufficient coverage or incapacity of interpreting gene/variant as pathogenic at the time of ES |
| *RPL5* | 603634 | exonic indel | chr1:g.92837552dup | ENST00000370321.8:c.624dup | ENSP00000359345.2:p.(Arg209AlafsTer8) | heterozygous, sporadic | Insufficient coverage or incapacity of interpreting gene/variant as pathogenic at the time of ES |
| *SASS6* | 609321 | non-exonic SNV  exonic SNV | chr1:g.100110291G>A  chr1:g.100107501T>C | ENST00000287482.6:c.861+1G>A  ENST00000287482.6:c.1199A>G | NA  ENSP00000287482.5:p.(Lys400Arg) | compound heterozygous | At least one non-coding pathogenic variant not captured by classical ES capture kits |
| *SCN2A* | 182390 | exonic SNV | chr2:g.165388683G>A | ENST00000375437.7:c.4877G>A | ENSP00000364586.2:p.(Arg1626Gln) | heterozygous, sporadic | Insufficient coverage or incapacity of interpreting gene/variant as pathogenic at the time of ES |
| *SLC25A3* | 600370 | non-exonic SNV | chr12:g.98595424A>G | ENST00000228318.8:c.158-9A>G | NA | homozygous | At least one non-coding pathogenic variant not captured by classical ES capture kits |
| *SLC6A1* | 137165 | exonic SNV | chr3:g.11031181G>A | ENST00000287766.10:c.1328G>A | ENSP00000287766.4:p.(Gly443Asp) | heterozygous, sporadic | Insufficient coverage or incapacity of interpreting gene/variant as pathogenic at the time of ES |
| *SMARCC1* | 601732 | exonic indel | chr3:g.47676689dup | ENST00000254480.10:c.1668dup | ENSP00000254480.5:p.(Asn557Ter) | heterozygous, inherited | Insufficient coverage or incapacity of interpreting gene/variant as pathogenic at the time of ES |
| *SPG7* | 602783 | exonic SNV  exonic indel | chr16:g.89546737C>T  chr16:g.89553855del | ENST00000645818.2:c.1529C>T  ENST00000645818.2:c.1998del | ENSP00000495795.2:p.(Ala510Val)  ENSP00000495795.2:p.(Met667TrpfsTer32) | compound heterozygous | Insufficient coverage or incapacity of interpreting gene/variant as pathogenic at the time of ES |
| *SPG7* | 602783 | exonic SNV  exonic SNV | chr16:g.89510539T>A  chr16:g.89546737C>T | ENST00000645818.2:c.233T>A  ENST00000645818.2:c.1529C>T | ENSP00000495795.2:p.(Leu78Ter)  ENSP00000495795.2:p.(Ala510Val) | unknown | Insufficient coverage or incapacity of interpreting gene/variant as pathogenic at the time of ES |
| *STX3*  *GALE* | 600876  606953 | exonic SNV  exonic SNV | chr11:g.59755624C>T  chr1:g.23796234C>A | ENST00000337979.9:c.19C>T  ENST00000617979.5:c.905G>T | ENSP00000338562.4:p.(Gln7Ter)  ENSP00000483375.1:p.(Gly302Val) | homozygous  homozygous | Insufficient coverage or incapacity of interpreting gene/variant as pathogenic at the time of ES |
| *TBX1* | 602054 | CNV | chr22:g.18947001_21390000del | NA | NA | heterozygous, sporadic | Structural variant not reported by ES and array-CGH |
| *TBX15* | 604127 | non-exonic SNV | chr1:g.118914112T>A | ENST00000207157.7:c.608+3A>T | NA | homozygous | At least one non-coding pathogenic variant not captured by classical ES capture kits |
| *TMEM163* | 618978 | exonic SNV | chr2:g.134713295A>G | ENST00000281924.6:c.227T>C | ENSP00000281924.6:p.(Leu76Pro) | heterozygous, sporadic | Insufficient coverage or incapacity of interpreting gene/variant as pathogenic at the time of ES |
| *TSC1* | 605284 | non-exonic SNV | chr9:g.132921451T>C | ENST00000642745.1:c.664-15A>G | NA | heterozygous, inherited | At least one non-coding pathogenic variant not captured by classical ES capture kits |
| *UFM1* | 611481 | non-exonic indel | chr13:g.38349765_38349767del | ENST00000451826.2:n.322+171_322+173del | NA | homozygous | At least one non-coding pathogenic variant not captured by classical ES capture kits |
| *USP9X* | 300072 | exonic indel | chrX:g.41196324_41196344del | ENST00000378308.7:c.4051_4071del | ENSP00000367558.2:p.(Leu1351_Leu1357del) | heterozygous, sporadic | Insufficient coverage or incapacity of interpreting gene/variant as pathogenic at the time of ES |
| *VAC14* | 604632 | exonic SNV  exonic SNV | chr16:g.70698720T>C  chr16:g.70698834T>C | ENST00000261776.10:c.1753A>G  ENST00000261776.10:c.1662-23A>G | ENSP00000261776.5:p.(Met585Val)  ENSP00000261776.5:p.(?) | compound heterozygous | Insufficient coverage or incapacity of interpreting gene/variant as pathogenic at the time of ES |
| *VPS13D* | 608877 | exonic indel  exonic SNV | chr1:g.12276238dup  chr1:g.12456014C>T | ENST00000620676.6:c.2650dup  ENST00000620676.6:c.12350C>T | ENSP00000478104.1:p.(Ile884AsnfsTer4)  ENSP00000478104.1:p.(Ser4117Leu) | compound heterozygous | Insufficient coverage or incapacity of interpreting gene/variant as pathogenic at the time of ES |
| *WDR62* | 613583 | non-exonic SNV  exonic SNV | chr19:g.36071719A>G  chr19:g.36097074C>T | ENST00000401500.7:c.1043+3A>G  ENST00000401500.7:c.2515C>T | NA  ENSP00000384792.1:p.(Arg839Trp) | compound heterozygous | At least one non-coding pathogenic variant not captured by classical ES capture kits |
| *WNT10A* | 606268 | exonic SNV | chr2:g.218890234G>A | ENST00000258411.8:c.627G>A | ENSP00000258411.3:p.(Trp209Ter) | heterozygous, inherited | Insufficient coverage or incapacity of interpreting gene/variant as pathogenic at the time of ES |
| *ZBTB20* | 606025 | exonic SNV | chr3:g.114339418G>T | ENST00000675478.1:c.1813C>A | ENSP00000501561.1:p.(Pro605Thr) | heterozygous, sporadic | Insufficient coverage or incapacity of interpreting gene/variant as pathogenic at the time of ES |
| *ZMYM2* | 602221 | exonic SNV | chr13:g.20061141C>G | ENST00000610343.5:c.2828C>G | ENSP00000479904.1:p.(Ser943Ter) | heterozygous, sporadic | Insufficient coverage or incapacity of interpreting gene/variant as pathogenic at the time of ES |
| dup(16)(p12.2) | - | large CNV | chr16:g.23419258_23431747dup | NA | NA | heterozygous, inherited | Structural variant not reported by ES (array-CGH not performed previously) |
| del(X)(q23q23) | - | large CNV | chrX:g.110025622_110209539del | NA | NA | hemizygous, sporadic | Structural variant not reported by ES (array-CGH not performed previously) |

array comparative genomic hybridization : array-CGH; CNV: copy number variant; ES: exome sequencing; SNV: single nucleotide variant

# Tumor topography among the 1,940 complete first consecutive prescriptions for cancers

| **Tumoral topography CIMO-3** | TOTAL  (N=1,564)* | Frequency (%) |
| --- | --- | --- |
| Brain, eye | 383 | 24.5% |
| Digestive organs | 194 | 12.4% |
| Bone | 161 | 10.3% |
| Breast | 142 | 9.1% |
| Female genital organs | 112 | 7.2% |
| Haematopoietic and reticuloendothelial system | 99 | 6.3% |
| Respiratory system and intrathoracic organs | 98 | 6.3% |
| Thyroid and other endocrine glands | 82 | 5.2% |
| Urinary tract | 77 | 4.9% |
| Peritoneum and retroperitoneum | 67 | 4.3% |
| Connective, subcutaneous and other soft tissues | 49 | 3.1% |
| Lymph nodes | 40 | 2.6% |
| Skin | 31 | 2.0% |
| Lip, oral cavity and pharynx | 13 | 0.8% |
| Peripheral nerves and autonomic nervous system | 11 | 0.7% |
| Male genitalia | 5 | 0.3% |

* data available with information clearly indicated with CIMO-3 topographical codes in 1,564/1,940 patients.

# Tumor morphological characteristics among the 1,940 complete first consecutive prescriptions for cancers

| Tumoral morphology CIMO-3 | TOTAL  (N=1,723) | Frequency  (%) |
| --- | --- | --- |
| Adenocarcinoma | 570 | 33.1% |
| Other sarcoma | 211 | 12.2% |
| Leukemia | 95 | 5.5% |
| Glioblastoma | 88 | 5.5% |
| Other glioma | 92 | 5.3% |
| Melanoma | 66 | 3.8% |
| Neuroblastoma | 57 | 3.3% |
| Osteosarcoma | 57 | 3.3% |
| Corticosurrenaloma | 30 | 1.7% |
| Medulloblastoma | 29 | 1.7% |
| Anaplasic astrocytoma | 28 | 1.6% |
| Leiomyosarcoma | 26 | 1.5% |
| Mesothelioma | 23 | 1.3% |
| Astrocytoma | 22 | 1.3% |
| Nephroblastoma | 19 | 1.1% |
| Lymphoma | 18 | 1.0% |
| Adrenocortical | 16 | 0.9% |
| Chordoma | 9 | 0.5% |
| Paraganglioma / pheochromocytoma | 8 | 0.5% |
| Hepatoblastoma | 7 | 0.4% |
| GIST | 3 | 0.2% |
| Desmoisd tumor | 2 | 0.1% |
| Pancreatoblastoma | 2 | 0.1% |
| Other | 245 | 14.2% |

* data available with information clearly indicated with CIMO-3 morphological codes in 1,723/1,940 patients.

Of note, a parallel study (Multisarc NCT03784014) is ongoing in first-line metastatic sarcoma, with 346 patients enrolled on 12/31/2022.

# List of the 40 genes most frequently mutated with somatic SNVs/Indels of interest returned to MTB for discussing actionability and treatment proposition among the 1,940 complete first consecutive prescriptions for cancers.

| **Gene with SNVs & Indels** | **AURAGEN** | | **SeqOIA** | | **TOTAL** | |
| --- | --- | --- | --- | --- | --- | --- |
|  | **number of SNVs & Indels** | **frequence of SNVs & Indels  (%)** | **number of SNVs & Indels** | **frequence of SNVs & Indels  (%)** | **number of SNVs & Indels** | **frequence of SNVs & Indels  (%)** |
| ***TP53*** | **160** | **32,79%** | **360** | **33,87%** | **520** | **33,5%** |
| ***TERT*** | **83** | **17,01%** | **79** | **7,43%** | **162** | **10,4%** |
| ***PIK3CA*** | **36** | **7,38%** | **83** | **7,81%** | **119** | **7,7%** |
| ***KRAS*** | **32** | **6,56%** | **66** | **6,21%** | **98** | **6,3%** |
| ***ATRX*** | **54** | **11,07%** | **35** | **3,29%** | **89** | **5,7%** |
| ***NF1*** | **26** | **5,33%** | **63** | **5,93%** | **89** | **5,7%** |
| *PTEN* | 33 | 6,76% | 43 | 4,05% | 76 | 4,9% |
| *KMT2D* | 17 | 3,48% | 58 | 5,46% | 75 | 4,8% |
| *RB1* | 29 | 5,94% | 46 | 4,33% | 75 | 4,8% |
| *ARID1A* | 15 | 3,07% | 58 | 5,46% | 73 | 4,7% |
| *KMT2C* | 30 | 6,15% | 42 | 3,95% | 72 | 4,6% |
| *APC* | 17 | 3,48% | 54 | 5,08% | 71 | 4,6% |
| *ATM* | 10 | 2,05% | 54 | 5,08% | 64 | 4,1% |
| *BAP1* | 8 | 1,64% | 44 | 4,14% | 52 | 3,4% |
| *IDH1* | 40 | 8,20% | 12 | 1,13% | 52 | 3,4% |
| *BRCA2* | 7 | 1,43% | 42 | 3,95% | 49 | 3,2% |
| *SETD2* | 14 | 2,87% | 35 | 3,29% | 49 | 3,2% |
| *LRP1B* | 8 | 1,64% | 40 | 3,76% | 48 | 3,1% |
| *BRAF* | 21 | 4,30% | 25 | 2,35% | 46 | 3,0% |
| *NOTCH1* | 15 | 3,07% | 31 | 2,92% | 46 | 3,0% |
| *FAT4* | 11 | 2,25% | 33 | 3,10% | 44 | 2,8% |
| *CTNNB1* | 5 | 1,02% | 38 | 3,57% | 43 | 2,8% |
| *CSMD3* | 10 | 2,05% | 30 | 2,82% | 40 | 2,6% |
| *SMARCA4* | 10 | 2,05% | 29 | 2,73% | 39 | 2,5% |
| *NRAS* | 17 | 3,48% | 21 | 1,98% | 38 | 2,5% |
| *CDKN2A* | 10 | 2,05% | 27 | 2,54% | 37 | 2,4% |
| *EGFR* | 18 | 3,69% | 19 | 1,79% | 37 | 2,4% |
| *GNAS* | 7 | 1,43% | 29 | 2,73% | 36 | 2,3% |
| *PIK3R1* | 14 | 2,87% | 22 | 2,07% | 36 | 2,3% |
| *FAT3* | 14 | 2,87% | 20 | 1,88% | 34 | 2,2% |
| *NOTCH2* | 3 | 0,61% | 31 | 2,92% | 34 | 2,2% |
| *PBRM1* | 4 | 0,82% | 30 | 2,82% | 34 | 2,2% |
| *H3F3A* | 20 | 4,10% | 13 | 1,22% | 33 | 2,1% |
| *ALK* | 11 | 2,25% | 21 | 1,98% | 32 | 2,1% |
| *BCOR* | 13 | 2,66% | 19 | 1,79% | 32 | 2,1% |
| *CIC* | 18 | 3,69% | 14 | 1,32% | 32 | 2,1% |
| *CREBBP* | 8 | 1,64% | 24 | 2,26% | 32 | 2,1% |
| *NF2* | 9 | 1,84% | 23 | 2,16% | 32 | 2,1% |
| *SF3B1* | 4 | 0,82% | 28 | 2,63% | 32 | 2,1% |
| *TET2* | 10 | 2,05% | 22 | 2,07% | 32 | 2,1% |

# List of the 41 genes most frequently implicated in somatic CNVs of interest returned to the MTB for discussing actionability and treatment proposition among the 1,940 complete first consecutive prescriptions for cancers.

| **Gene with CNVs (amplifications, deletions)** | **AURAGEN** | | **SeqOIA** | | **TOTAL** | |
| --- | --- | --- | --- | --- | --- | --- |
|  | **number of CNV** | **frequence of CNV  (%)** | **number of CNV** | **frequence of CNV  (%)** | **number of CNV** | **frequence of CNV  (%)** |
| ***CDKN2A*** | **109** | **37,20%** | **143** | **18,43%** | **252** | **23,6%** |
| ***CDKN2B*** | **60** | **20,48%** | **93** | **11,98%** | **153** | **14,3%** |
| ***TP53*** | **73** | **24,91%** | **35** | **4,51%** | **108** | **10,1%** |
| ***MTAP*** | **17** | **5,80%** | **68** | **8,76%** | **85** | **8,0%** |
| ***RB1*** | **33** | **11,26%** | **37** | **4,77%** | **70** | **6,5%** |
| ***ATRX*** | **6** | **2,05%** | **55** | **7,09%** | **61** | **5,7%** |
| ***PTEN*** | **23** | **7,85%** | **34** | **4,38%** | **57** | **5,3%** |
| ***EGFR*** | **21** | **7,17%** | **35** | **4,51%** | **56** | **5,2%** |
| *AMER1* | 1 | 0,34% | 43 | 5,54% | 44 | 4,1% |
| *MYC* | 15 | 5,12% | 29 | 3,74% | 44 | 4,1% |
| *TERT* | 5 | 1,71% | 39 | 5,03% | 44 | 4,1% |
| *KDM5C* | 1 | 0,34% | 42 | 5,41% | 43 | 4,0% |
| *BCOR* | 0 | 0,00% | 40 | 5,15% | 40 | 3,7% |
| *DDX3X* | 0 | 0,00% | 39 | 5,03% | 39 | 3,6% |
| *CCND1* | 7 | 2,39% | 31 | 3,99% | 38 | 3,6% |
| *CDK4* | 18 | 6,14% | 20 | 2,58% | 38 | 3,6% |
| *MDM2* | 17 | 5,80% | 21 | 2,71% | 38 | 3,6% |
| *MED12* | 0 | 0,00% | 38 | 4,90% | 38 | 3,6% |
| *SMC1A* | 0 | 0,00% | 38 | 4,90% | 38 | 3,6% |
| *PHF6* | 0 | 0,00% | 37 | 4,77% | 37 | 3,5% |
| *RBM10* | 0 | 0,00% | 36 | 4,64% | 36 | 3,4% |
| *ZMYM3* | 0 | 0,00% | 35 | 4,51% | 35 | 3,3% |
| *FGFR1* | 8 | 2,73% | 26 | 3,35% | 34 | 3,2% |
| *STAG2* | 1 | 0,34% | 33 | 4,25% | 34 | 3,2% |
| *ZRSR2* | 0 | 0,00% | 33 | 4,25% | 33 | 3,1% |
| *PDGFRA* | 9 | 3,07% | 23 | 2,96% | 32 | 3,0% |
| *CCNE1* | 4 | 1,37% | 26 | 3,35% | 30 | 2,8% |
| *MAP2K4* | 4 | 1,37% | 26 | 3,35% | 30 | 2,8% |
| *NSD3* | 5 | 1,71% | 24 | 3,09% | 29 | 2,7% |
| *RPL10* | 1 | 0,34% | 28 | 3,61% | 29 | 2,7% |
| *KIT* | 7 | 2,39% | 21 | 2,71% | 28 | 2,6% |
| *PTPRD* | 5 | 1,71% | 23 | 2,96% | 28 | 2,6% |
| *ATP2B3* | 1 | 0,34% | 26 | 3,35% | 27 | 2,5% |
| *KDM6A* | 1 | 0,34% | 26 | 3,35% | 27 | 2,5% |
| *KRAS* | 9 | 3,07% | 18 | 2,32% | 27 | 2,5% |
| *MLLT3* | 8 | 2,73% | 18 | 2,32% | 26 | 2,4% |
| *FHIT* | 6 | 2,05% | 19 | 2,45% | 25 | 2,3% |
| *GATA1* | 1 | 0,34% | 24 | 3,09% | 25 | 2,3% |
| *MYCN* | 12 | 4,10% | 13 | 1,68% | 25 | 2,3% |
| *STK11* | 3 | 1,02% | 22 | 2,84% | 25 | 2,3% |
| *UBR5* | 7 | 2,39% | 18 | 2,32% | 25 | 2,3% |

# Secondary use of data for research and data sharing

Although findings from the *PFMG2025* initiative are being deployed in the French healthcare system, it was designed to provide a continuum between research and care, with the objective of sharing data both at a national and international level and to enable researchers to re-use data.

The massive secondary use of data for research will be possible within the national IT and data infrastructure under construction (*CAD*). Among other things, it will bring together clinical and genomic data from *FMGlabs* and from the 4 pilot projects of the *PFMG2025*. Ultimately, *CAD* will have intensive calculation and data storage capacities, which will gradually reach tens of petabytes.

Data reuse of the *PFMG2025* initiative for research is part of an open science dynamic. The access to data is intended to be as widespread as possible, while ensuring data security and respecting a certain number of scientific and ethical criteria. They were defined by a dedicated working group and validated by the governance of the *PFMG2025* (contribution of the project to the common good and relevance of the intended uses, feasibility of the project on the basis of the available data and technical expertise, suitability of the *PFMG2025* dataset for the research project, sensitivity of the results that will be downloaded from the *CAD* once the project is completed, compliance with the GDPR regulation and the regulatory framework). The multidisciplinary Scientific and Ethics Committee (CSE) of the *CAD* is responsible for ensuring that research projects having access to data respect these criteria. To date, ten research projects have received approval to access a *PFMG2025* dataset.

After validation by the CSE, the *PFMG2025* data necessary for the project will be made available to the research team within a dedicated secure area. Researchers will also have access to analysis tools and the necessary computing capacity. In addition, they will have the possibility of importing their own bioinformatics analysis tools and data into this same secure area. They will thus be able to carry out their research project in a dedicated environment, thus guaranteeing both data security and the confidentiality of their work. It will enable researchers to re-analyze data from cohorts of interest, either using new bioinformatics tools currently not used by *FMGlabs*, or by going beyond affecting well-established disease genes in RD, or by using innovative re-analysis strategies.

Data sharing is essential in RD in order to improve our knowledge, whether through the extension of already known phenotypes or the identification of new genes or molecular mechanisms in ultra-RD. Data from the *PFMG2025* initiative have already contributed to the publication of such scientific results.^17-54^ In cancer *PFMG2025* data have contributed to a pilot study which suggested that whole-genome/-exome and RNA sequencing could guide therapy in a small subset of patients with advanced hepatocellular carcinoma and hepato-cholangiocarcinoma (H-CCK) progressing under atezolizumab/bevacizumab^55^ and also identified germline actionable variants in osteosarcoma.^56^

In addition, *CAD* is part of a European dynamic, in particular through the French participation to the '1+ Million Genomes' (1+MG) initiative. *CAD* participates in the European Genomic Data Infrastructure project aimed at implementing the sharing of genomic data on a European scale. Thus, the *PFMG2025* genomic data will be made available on a European scale and French researchers will be able to access those produced in other countries.

# References

1. Binquet C, Lejeune C, Faivre L, et al. Genome Sequencing for Genetics Diagnosis of Patients With Intellectual Disability: The DEFIDIAG Study. *Front Genet* 2022; **12**: 766964.
2. Lejeune C, Robert-Viard C, Meunier-Beillard et al. The Economic, Medical and Psychosocial Consequences of Whole Genome Sequencing for the Genetic Diagnosis of Patients With Intellectual Disability: The DEFIDIAG Study Protocol. *Front Genet* 2022; **13**: 852472.
3. Lejeune C, Amado IF; DEFIDIAG study group, FHU Translad and Aviesan. Valuing genetic and genomic testing in France: current challenges and latest evidence. *J Community Genet* **2022**; 13: 477-85.
4. Italiano A, Dinart D, Soubeyran I et al. Molecular profiling of advanced soft-tissue sarcomas: the MULTISARC randomized trial. *BMC Cancer* 2021; **21**:1180.
5. FMG2025 Workflow study group (Alliance nationale des sciences de la Vie et de la santé); Auzanneau C, Bacq D, et al. Feasibility of high-throughput sequencing in clinical routine cancer care: lessons from the cancer pilot project of the FMG2025. *ESMO Open* 2020; **5**: e000744.
6. Froger-Lefebvre J, Lade Q, Vallier E, Bourgain C. E-prescription and invisible work in genomics in France. *Front Sociol* 2023; **8**: 1152364.
7. Steiert TA, Parra G, Gut M, et al. A critical spotlight on the paradigms of FFPE-DNA sequencing. Nucleic Acids Res 2023; **51**: 7143-62.
8. Richards S, Aziz N, Bale S, et al. Standards and guidelines for the interpretation of sequence variants: a joint consensus recommendation of the American College of Medical Genetics and Genomics and the Association for Molecular Pathology. *Genet Med* 2015; **17**: 405–24.
9. Austin-Tse CA, Jobanputra V, Perry DL, et al. Best practices for the interpretation and reporting of clinical whole genome sequencing. *NPJ Genom Med* 2022; **7**: 27.
10. Riggs ER, Andersen EF, Cherry AM, et al. Technical standards for the interpretation and reporting of constitutional copy-number variants: a joint consensus recommendation of the American College of Medical Genetics and Genomics (ACMG) and the Clinical Genome Resource (ClinGen). *Genet Med* 2020; **22**: 245-57.
11. Brandt T, Sack LM, Arjona D, et al. Adapting ACMG/AMP sequence variant classification guidelines for single-gene copy number variants. *Genet Med* 2020; **22**: 336-44.
12. Clark MM, Stark Z, Farnaes L, Tan TY, et al. Meta-analysis of the diagnostic and clinical utility of genome and exome sequencing and chromosomal microarray in children with suspected genetic diseases. *NPJ Genom Med* 2018; **3**: 16.
13. Sun Y, Peng J, Liang D, et al. Genome sequencing demonstrates high diagnostic yield in children with undiagnosed global developmental delay/intellectual disability: A prospective study. *Hum Mutat* 2022; **43**:568-81.
14. Lindstrand A, Ek M, Kvarnung M, et al. Genome sequencing is a sensitive first-line test to diagnose individuals with intellectual disability. *Genet Med* 2022; **24**: 2296-2307.
15. Weisschuh N, Mazzola P, Zuleger T, et al. Diagnostic genome sequencing improves diagnostic yield: a prospective single-centre study in 1000 patients with inherited eye diseases. *J Med Genet* 2024; **61**: 186-195.
16. 100,000 Genomes Project Pilot Investigators, Smedley D, Smith KR, Martin A, Thomas EA, McDonagh EM, et al. 100,000 genomes pilot on rare-disease diagnosis in health care - preliminary report. *N Engl J Med* 2021; **385**: 1868–80.
17. Rucheton B, Ewenczyk C, Gaignard P, et al. Adult Cerebellar Ataxia, Axonal Neuropathy, and Sensory Impairments Caused by Biallelic SCO2 Variants. *Neurol Genet* 2021; **7**: e630.
18. Riou MC, de La Dure-Molla M, Kerner S, et al. Oral Phenotype of Singleton-Merten Syndrome: A Systematic Review Illustrated With a Case Report. *Front Genet* 2022; **13**: 875490.
19. Guerrini R, Mei D, Kerti-Szigeti K, et al. Phenotypic and genetic spectrum of ATP6V1A encephalopathy: a disorder of lysosomal homeostasis. *Brain* 2022; **145**: 2687-703.
20. Christensen MB, Levy AM, Mohammadi NA, et al. Biallelic variants in ZNF142 lead to a syndromic neurodevelopmental disorder. *Clin Genet* 2022; **102**: 98-109.
21. Rajan DS, Kour S, Fortuna TR, et al. Autosomal Recessive Cerebellar Atrophy and Spastic Ataxia in Patients With Pathogenic Biallelic Variants in GEMIN5. *Front Cell Dev Biol* 2022; **10**: 783762.
22. Münch J, Engesser M, Schönauer R, et al. Biallelic pathogenic variants in roundabout guidance receptor 1 associate with syndromic congenital anomalies of the kidney and urinary tract. *Kidney Int* 2022; **101**: 1039-1053.
23. Kumble S, Levy AM, Punetha J, et al. The clinical and molecular spectrum of QRICH1 associated neurodevelopmental disorder. *Hum Mutat* 2022; 43: 266-82.
24. Lacombe D, Van-Gils J, Lebrun M, et al. Hemidystonia with polymicrogyria is part of ATP1A3-related disorders. *Brain Dev* 2022; **44**: 567-70.
25. Sabbagh Q, Alkar F, Patte K, et al. A second individual with rhizomelic spondyloepimetaphyseal dysplasia and homozygous variant in GNPNAT1. *Eur J Med Genet* 2022; **65**: 104495.
26. Zech M, Kumar KR, Reining S, et al. Biallelic AOPEP Loss-of-Function Variants Cause Progressive Dystonia with Prominent Limb Involvement. *Mov Disord* 2022; 37: 137-47.
27. von der Lippe C, Tveten K, Prescott TE, et al. Heterozygous variants in ZBTB7A cause a neurodevelopmental disorder associated with symptomatic overgrowth of pharyngeal lymphoid tissue, macrocephaly, and elevated fetal hemoglobin. *Am J Med Genet A* 2022; **188** :272-82.
28. Lunati A, Petit A, Lapillonne H, et al. VPS4A mutation in syndromic congenital hemolytic anemia without obvious signs of dyserythropoiesis. *Am J Hematol* 2021; **96**: E121-3.
29. Papadopoulos T, Gaignard P, Schiff M, el al. New description of an MRPS2 homozygous patient: Further features to help expend the phenotype. *Eur J Med Genet* 2023; **67**: 104889.
30. Durin Z, Raynor A, Fenaille F, et al. Efficacy of oral manganese and D-galactose therapy in a patient bearing a novel TMEM165 variant. *Transl Res* 2023; **S1931-5244**: 00185-8.
31. Li D, Wang Q, Bayat A, Battig MR, et al. Spliceosome malfunction causes neurodevelopmental disorders with overlapping features. *J Clin Invest* 2023; e171235.
32. Chevrollier A, Bonnard AA, Ruaud L, et al. Homozygous MFN2 variants causing severe antenatal encephalopathy with clumped mitochondria. *Brain* 2023; awad347.
33. Sperelakis-Beedham B, Ruaud L, Vial Y, et al. Expanding the phenotype of GTF2E2-associated trichothiodystrophy. *J Eur Acad Dermatol Venereol* 2023 Oct 4. doi: 10.1111/jdv.19545.
34. Poggio E, Barazzuol L, Salmaso A, et al. ATP2B2 de novo variants as a cause of variable neurodevelopmental disorders that feature dystonia, ataxia, intellectual disability, behavioral symptoms, and seizures. *Genet Med* 2023; **25**: 100971.
35. Parra A, Rabin R, Pappas J, et al. Clinical Heterogeneity and Different Phenotypes in Patients with SETD2 Variants: 18 New Patients and Review of the Literature*. Genes (Basel)* 2023; **14**: 1179.
36. Rive Le Gouard N, Nicolle R, Lefebvre M, et al. First reports of fetal SMARCC1 related hydrocephalus. *Eur J Med Genet* 2023; **66**: 104797.
37. Frost FG, Morimoto M, Sharma P, et al. Bi-allelic SNAPC4 variants dysregulate global alternative splicing and lead to neuroregression and progressive spastic paraparesis. *Am J Hum Genet* 2023; **110**: 663-80.
38. Khatri D, Putoux A, Cologne A, et al. Deficiency of the minor spliceosome component U4atac snRNA secondarily results in ciliary defects in human and zebrafish. *Proc Natl Acad Sci U S A* 2023; **120**: e2102569120.
39. Happ HC, Sadleir LG, Zemel M, et al. Neurodevelopmental and Epilepsy Phenotypes in Individuals With Missense Variants in the Voltage-Sensing and Pore Domains of KCNH5. *Neurology* 2023; **100**: e603-e615.
40. Pacot L, Pelletier V, Chansavang A, et al. Contribution of whole genome sequencing in the molecular diagnosis of mosaic partial deletion of the NF1 gene in neurofibromatosis type 1. *Hum Genet* 2023; **142**: 1-9.
41. Sabbagh Q, Haghshenas S, Piard J, et al. Clinico-biological refinement of BCL11B-related disorder and identification of an episignature: A series of 20 unreported individuals. *Genet Med* 2023; **26**: 101007.
42. Aubert-Mucca M, Janel C, Porquet-Bordes V, et al. Clinical heterogeneity of NADSYN1-associated VCRL syndrome. *Clin Genet* 2023; **104**:114-20.
43. Reis LM, Chassaing N, Bardakjian T, Thompson S, Schneider A, Semina EV. ARHGAP35 is a novel factor disrupted in human developmental eye phenotypes. *Eur J Hum Genet* 2023; **31**: 363-7.
44. Nicolle R, Boutaud L, Loeuillet L, et al. Expanding the phenotypic spectrum of LIG4 pathogenic variations: neuro-histopathological description of 4 fetuses with stenosis of the aqueduct. *Eur J Hum Genet* 2024; **32**:545-9.
45. Tusseau M, Eyries M, Chatron N, et al. Genome sequencing identify chromosome 9 inversions disrupting ENG in 2 unrelated HHT families. *Eur J Med Genet* 2024; **68**: 104919.
46. Szot JO, Cuny H, Martin EM, et al. A metabolic signature for NADSYN1-dependent congenital NAD deficiency disorder. *J Clin Invest* 2024; **134**: e174824.
47. Previdi A, Dubourg C, Cormier Daire V, et al. Novel variant in LRP6 associated with unusual and severe clinical presentation: Case report. *Clin Genet* 2024, **105**:666-70.
48. Dohrn MF, Bademci G, Rebelo AP, et al. Recurrent ATP1A1 variant Gly903Arg causes developmental delay, intellectual disability, and autism. *Ann Clin Transl Neurol* 2024, **11**:1075-9.
49. Plaisancié J, Chesneau B, Fares-Taie L, et al. Structural Variant Disrupting the Expression of the Remote FOXC1 Gene in a Patient with Syndromic Complex Microphthalmia. *Int J Mol Sci* 2024; **25**: 2669.
50. Billon C, Piccoli GB, de sainte Agathe JM, Genome-wide analysis identifies MYH11 compound heterozygous variants leading to visceral myopathy corresponding to late-onset form of megacystis-microcolon-intestinal hypoperistalsis syndrome. *Mol Genet Genomics* 2024, **299**: 44.
51. Chaussenot A, Ayrignac X, Chatron N, et al. Loss of heterozygosity in CCM2 cDNA revealing a structural variant causing multiple cerebral cavernous malformations. *Eur J Hum Genet* 2024 May 16. doi: 10.1038/s41431-024-01626-7.
52. Theuriet J, Marte S, Isapof A, et al. A previously unreported NARS1 variant causes dominant distal hereditary motor neuropathy in a French family. *J Peripher Nerv Syst* 2024 May 20. doi: 10.1111/jns.12635.
53. Watts LM, Bertoli M, Attie-Bitach T, et al. The phenotype of MEGF8-related Carpenter syndrome (CRPT2) is refined through the identification of eight new patients. *Eur J Hum Genet* 2024 May 17. doi: 10.1038/s41431-024-01624-9.
54. Layo-Carris DE, Lubin EE, Sangree AK, et al. Expanded phenotypic spectrum of neurodevelopmental and neurodegenerative disorder Bryant-Li-Bhoj syndrome with 38 additional individuals. *Eur J Hum Genet* 2024 Apr 27. doi: 10.1038/s41431-024-01610-1.
55. Limousin W, Laurent-Puig P, Ziol M, et al. Molecular-based targeted therapies in patients with hepatocellular carcinoma and hepato-cholangiocarcinoma refractory to atezolizumab/bevacizumab. *J Hepatol* 2023;**79**: 1450-8.
56. Mouren A, Chansavang A, Hamzaoui N, et al. A de novo germline pathogenic BRCA1 variant identified following an osteosarcoma pangenomic molecular analysis. *Fam Cancer* 2024 May 19. doi: 10.1007/s10689-024-00393-0.
